# Solution-processable and functionalizable ultra-high molecular weight polymers via topochemical synthesis

**DOI:** 10.1038/s41467-021-27090-1

**Published:** 2021-11-24

**Authors:** Christopher L. Anderson, He Li, Christopher G. Jones, Simon J. Teat, Nicholas S. Settineri, Eric A. Dailing, Jiatao Liang, Haiyan Mao, Chongqing Yang, Liana M. Klivansky, Xinle Li, Jeffrey A. Reimer, Hosea M. Nelson, Yi Liu

**Affiliations:** 1 The Molecular Foundry, Lawrence Berkeley National Laboratory, One Cyclotron Road, Berkeley, CA 94720 USA; 2 Department of Chemistry, University of California, Berkeley, Berkeley, CA 94720 USA; 3 Materials Sciences Division, Lawrence Berkeley National Laboratory, One Cyclotron Road, Berkeley, CA 94720 USA; 4 Department of Chemistry and Biochemistry, University of California, Los Angeles, Los Angeles, CA 90095 USA; 5 Advanced Light Source, Lawrence Berkeley National Laboratory, One Cyclotron Road, Berkeley, CA 94720 USA; 6 Department of Chemical and Biomolecular Engineering, University of California, Berkeley, Berkeley, CA 94720 USA

**Keywords:** Polymer synthesis, Polymer characterization

## Abstract

Topochemical polymerization reactions hold the promise of producing ultra-high molecular weight crystalline polymers. However, the totality of topochemical polymerization reactions has failed to produce ultra-high molecular weight polymers that are both soluble and display variable functionality, which are restrained by the crystal-packing and reactivity requirements on their respective monomers in the solid state. Herein, we demonstrate the topochemical polymerization reaction of a family of *para*-azaquinodimethane compounds that undergo facile visible light and thermally initiated polymerization in the solid state, allowing for the first determination of a topochemical polymer crystal structure resolved via the cryoelectron microscopy technique of microcrystal electron diffraction. The topochemical polymerization reaction also displays excellent functional group tolerance, accommodating both solubilizing side chains and reactive groups that allow for post-polymerization functionalization. The thus-produced soluble ultra-high molecular weight polymers display superior capacitive energy storage properties. This study overcomes several synthetic and characterization challenges amongst topochemical polymerization reactions, representing a critical step toward their broader application.

## Abstract (web summary)

Topochemical polymerization reactions produce ultra-high molecular weight crystalline polymers. Here the authors show a family of para-azaquinodimethane compounds that undergo facile visible light and thermally initiated polymerization in the solid state and the crystal structure has been resolved via cryoelectron microscopy.

## Introduction

Topochemical polymerizations (TCPs) are solid-state transformations, wherein monomers crystallize in an alignment such that some external stimulus—most often heat and/or light—causes them to polymerize^1–5^. TCP reactions are freed from many of the constraints of their solution-based counterparts and are capable of producing ultra-high molecular weight (UHMW) polymers—those with number-averaged molecular weights (Mn) above 10^6^ Dalton^6–8^, in a stereospecific, regioregular, solvent-free, and catalyst-free manner. TCP reactions are relatively rare, however, due to the fact that TCP monomers must crystallize such that the distances between their reactive sites (d_CC_s) are small enough to allow for their polymerization to proceed without significant movement or deformation in each of the monomers^1,3,4,9^. The vast majority of examples make use of two categories of reactions: cycloaddition reactions that involve polyolefins^10–13^, oligo(aza)anthracenes^14–17^, alkynes/azide^18–21^ and alkene/azides^22^; and addition reactions between reactive groups, such as diynes^3,6,23–25^, triynes^26–28^, dienes^29–32^, trienes^33^, *para*-quinodimethanes^9,34–41^ and bis(indanone)s^8,42,43^. In addition to linear polymers, TCP reactions have also been used to produce a number of intriguing extended materials, such as porous 2D^25,42,44–51^ and 3D polymer crystals^52^.

To date, the utility of TCP reactions as a polymerization technique has not been adequately demonstrated as the crystalline polymers so produced are often insoluble, or in the cases of soluble ones, their molecular weights are typically quite limited^12^. In addition, the functional groups cannot be varied without drastically affecting their TCP reactivity^6,9,42^. As hinted at by others previously, an alternative strategy to produce a wide variety of useful UHMW polymers from TCP reactions would be to make use of a monomeric structure that incorporates both the solubilizing sidechains and a reactive functional group that survives the TCP reaction, allowing for postpolymerization functionalization^3,23,53^. If successful, this method would represent one of the few routes to soluble UHMW polymers with functionality that is not significantly restrained by the polymerization conditions^54–57^.

In this work, we demonstrate that a family of *para*-azaquinodimethanes (AQMs) shows a tempered form of the reactivity displayed by their unsubstituted structural cousin, *para*-quinodimethane, undergoing robust light and thermally initiated single-crystal polymerization while accommodating both the reactive and solubilizing groups^58,59^. The bestowed solution processability enables the fabrication of dielectric film capacitors that show excellent capacitive energy storage properties.

## Results

### Monomer synthesis, topochemical polymerization, and characterization

**Fig. 1:**
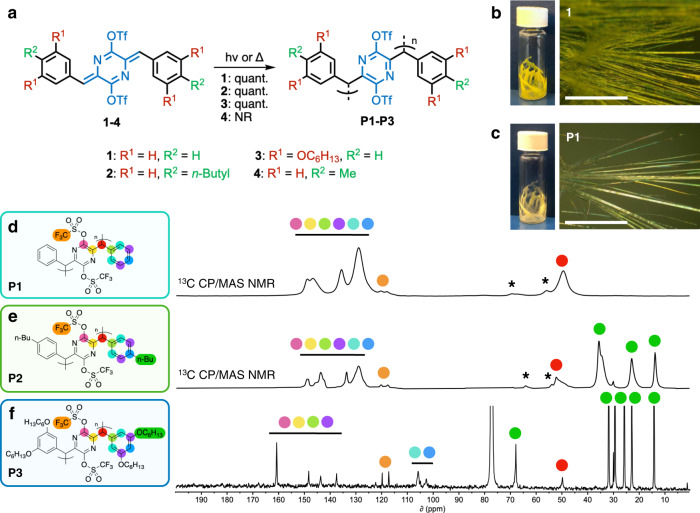
Single-crystal polymerization of a family of AQM ditriflates. **a** The structures of the AQM ditriflates, **1**–**4**, studied herein. Monomers **1**–**3** underwent topochemical polymerization under the effects of heat and/or light to produce polymers **P1**–**P3**. NR: no reaction. **b**–**c** Photographs and optical microscope images of vials containing crystals of (**b**) **1** and (**c**) **P1**, showing the typical morphology of crystals. Scale bar: 1 mm. **d**–**f** ^13^C-NMR spectra of polymers **P1–P3**. Cross-polarization/magic angle spinning (CP/MAS) solid-state ^13^C-NMR spectra of (**d**) **P1**, and (**e**) **P2** (asterisks denote spinning side bands). (**f**) Solution ^13^C-NMR spectrum of **P3** (solvent: CDCl_3_). All carbon resonances are annotated by colored circles. The resonances in d–f between 49–53 ppm (red) indicate the presence of the sp^3^ carbons generated during polymerization, and the CF_3_ multiplets (expected ratio of 1:3:3:1, but only the two highest peaks are observed) between 117 and 120 ppm (orange) show that the triflate groups remain intact.

The AQM ditriflates in this study (Fig. 1a) employ substituted phenyl or phenolic end groups, which are homologues of the previously reported thiophene-end capped AQMs^60,61^. The substitution of the aromatic end groups of these AQMs yielded an unexpected change in their solid-state reactivity. While the thienylidene AQMs are photochemically and thermally stable in the solid state, the new AQM series display remarkable solid-state reactivity that is suitable for TCP applications.

The synthesis of the AQM ditriflate monomers **1**–**4** was accomplished in high yield following a succinct synthetic route (Supplementary Fig. 2)^60–62^. The four monomers produced long-aspect ratio yellow needle or hair-like crystals under all of the attempted crystallization conditions (Fig. 1, Supplementary Fig. 3 and 4). Crystals of monomers **1**–**3** decolorized when exposed to ambient sunlight or heating above 80 °C, indicating that they undergo solid-state polymerization reactions to produce the nonconjugated poly-*p*-xylylene derivatives **P1**–**P3** (Fig. 1 and Supplementary Fig. 3, and Supplementary Movie 1)^9,35,41^. The solid-state reactivity of the phenyl-substituted AQM monomers **1**–**3** differs from the monomer **4**, which is not affected by light and/or heat. After polymerization, crystals of **P1** and **P2** become insoluble, while those of **P3** readily dissolve in many common mid-range polarity organic solvents. In contrast to previously reported TCP systems—including the closely related substituted *para*-quinodimethanes and quinone methides—the solid-state reactivity of diphenylidine-substituted AQMs were surprisingly forgiving to the inclusion of long alkyl groups^9,35,36,40,41^. Additionally, unlike many other methods of producing UHMW polymers, this method is insensitive to the presence of water and oxygen and requires no catalysts or auxiliaries^54–57^.

The structures of polymers **P1**–**P3** were confirmed by a variety of nuclear magnetic resonance (NMR) and infrared (IR) spectroscopic studies. The solubility of polymer **P3** allows for solution-phase ^1^H, ^13^C, and ^19^F NMR characterizations, which show broadened peaks typical of polymers along with chemical shifts and integrals consistent with the proposed structure (Fig. 1d–f and Supplementary Figs. 52–54). In particular, the characteristic resonance corresponding to the exocyclic methylene protons in the ^1^H NMR spectrum of **3** at *δ* = 6.82 ppm is absent in that of **P3** (Supplementary Fig. 59). Instead, a resonance corresponding to the xylyl protons at *δ* = 4.95 ppm is observed. The solution ^13^C-NMR spectrum of **P3**, along with cross-polarization/magic angle spinning (CP/MAS) solid-state ^13^C-NMR spectra of **P1** and **P2**, further corroborate the proposed polymer structures, showing the expected resonances corresponding to the xylyl carbons around 50 ppm and the absence of the exocyclic methylene carbon resonances of their respective monomers. As these NMR spectra are taken on unpurified polymer samples, it can be concluded that the conversions from **1**–**3** to **P1**–**P3** in the solid state proceed quantitatively. In addition, the features with a characteristic splitting at 117–121 ppm in the polymers’ ^13^C-NMR spectra can be attributed to the CF_3_ groups within the triflates appended to each repeat unit. This feature, together with a slightly broadened peak at –73.8 ppm in the ^19^F NMR spectrum of **P3**, confirms that the triflate groups appended to monomers **1**–**3** remain intact through the polymerization process (Supplementary Fig. 53).

The proposed TCP reaction was further supported by IR spectroscopic studies (Supplementary Figs. 5 and 6). The characteristic vibrational features at 1570–1635 cm^–1^ in the IR spectrum of **1** is not observed in the spectrum of **P1**^35,58^. With the help of computations, these features can be assigned to the stretching mode of the quinoidal AQM ring, supporting the conclusion that the quinoidal rings have been consumed during the polymerization reaction.

The solid-state polymerization of monomers **1** and **2** produces insoluble polymeric products, for which molecular weight information cannot be obtained experimentally. However, the solubility of **P3** allows for the evaluation of its molecular weight information via conventional size-exclusion chromatography (SEC). Monomer **3** could be crystallized via the slow-evaporation of its solutions in different common solvents, which, when polymerized, produce a variable range of molecular weights (Supplementary Fig. 7). The highest molecular weight polymer—produced from crystals of **3** grown via the slow evaporation of a toluene solution—yields a Mn = 1.9 × 10^6^, a Mw = 3.9 × 10^6^, and a polydispersity index of 1.99. The differences in molar mass and polydispersity are attributed to the variations of crystallite domain sizes in the monomer crystals, determined by defect sites in the parent crystal and fragmentation during the polymerization, which are highly solvent and process dependent^12^.

### Crystal structure determination by X-ray and electron diffractions

**Fig. 2:**
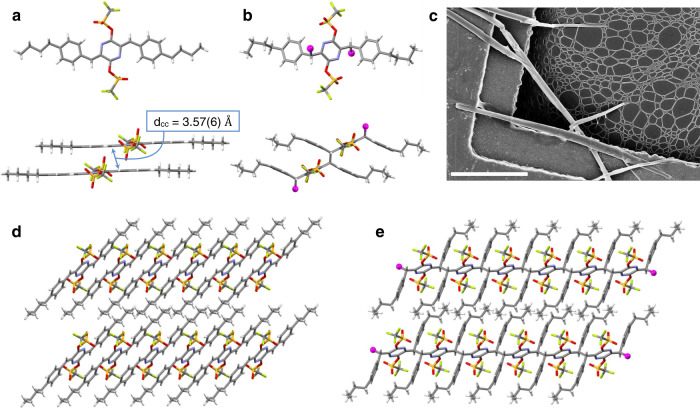
Solid-state structures of monomer 2 and polymer P2. **a** X-ray crystal structure of **2** showing molecular structure, and d_CC_—the distance between reactive sites of two neighboring molecules. **b** CryoEM structure of polymer **P2** showing the unit cell structure (top), and a dimeric unit (bottom). **c** Scanning electron microscopy image of crystals of **P2** on a TEM grid similar to those used to obtain its structure by cryoEM. Scale bar: 10 μm. **d** and **e** Analogous views of the columnar stacks of monomer **2** and the polymeric chains of **P2**. Atom color scheme: carbon = gray, nitrogen = blue, oxygen = red, sulfur = yellow, fluorine = green, hydrogen = white, magenta balls represent the truncated polymer chain in polymer **P2**.

Single-crystal X-ray diffraction studies of monomers **1**–**4** provide more insights into their solid-state packing and polymerization. In all four monomers, the central AQM unit displays a bond-length alternation pattern characteristic of a quinoidal ring and sits coplanar with its phenyl end groups (Fig. 2 and Supplementary Figs. 12–15). These π systems further stack into extended columns with close π-π interplanar distances in the range of 3.26–3.42 Å. Notably, the distances between the active methylenes on neighboring AQMs in the solid state (d_CC_s) in the three topochemically active monomers (**1**–**3**) are 3.62(6), 3.57(6), and 3.76(8) Å, respectively. Such small d_CC_ distances are a necessity in topochemical polymerizations, as they require minimal atomic movement or deformation of the unit cell in order to form the new bonds, and thus preserve macroscopic crystal integrity and produce high molecular weight polymers^4,8,9^. In contrast to monomers **1**–**3**, the reactive methylene carbons on each molecule of **4** are not close enough to either of its neighbors’ to allow for TCP. Large d_CC_ values of 4.98(3) and 5.13(8) Å—depending on which methylene carbons are presumed to react—are found in the solid-state of **4**, which are significantly larger than those in monomers **1**–**3** and other polymerizable examples in the *para*-quinodimethane family and explain its lack of topochemical reactivity^9,35,36,40,41^.

Powder X-ray diffraction (PXRD) studies of crystals of **1**–**3** and polymers **P1**–**P3** showed a retention of crystallinity upon polymerization but with apparent changes in the diffraction patterns in all three cases (Supplementary Fig. 11). In the case of **P2** and **P3**, the (100) diffraction peak position is nearly identical to that of the respective monomers, in accordance with very small lattice changes upon polymerization. Significant changes of the rest of the patterns, together with all other experimental evidences (DSC, NMR, IR, SEC, UV-vis, solubility), corroborate well with the complete monomer-to-polymer transformation. All attempts at obtaining a polymeric crystal structure using synchrotron X-ray crystallography proved unsatisfactory due to the loss of single-crystal quality after TCP reaction. As the polymerization propagates from the outer surface to the inner zone of the monomer crystal, the strain causes the crystals to break into smaller pieces. While further optimization of the TCP reaction at different temperatures and irradiation conditions may facilitate single-crystal X-ray studies, we have sought to a convenient alternative that requires minimal crystal growth effort. Through a facile dip-coating procedure, monomer **2** microcrystals were deposited on TEM grids, and polymerized in situ to provide **P2** microcrystals (Fig. 2, Supplementary Figs. 2, 20 and 21) that were structurally analyzed via the cryoelectron microscopy (cryoEM) technique of microcrystal electron diffraction (MicroED). This strategy obviates the challenges of obtaining TCP product crystal structures with X-ray diffraction and may prove useful in other contexts that were previously inaccessible through traditional structural characterization methods^63^. The successful crystallographic analysis of **P2**-coated grids yielded a 1 Å resolution structure of polymer **P2** in the solid state (Fig. 2, Supplementary Figs. 16 and 22). The structure of polymer **P2** shows the expected highly substituted poly-*p*-xylylene structure. Each repeat unit is joined in a single bond between two tetrahedral carbons with a regiospecificity analogous to the 1,6-addition polymerizations of *p*-QMs to form poly-*p*-xylylenes. The planar monomer **2** molecules deform upon polymerization, such that the pyrazine rings in each repeat unit of **P2** are coplanar with each other, and 66° out of plane with their appended 4-*n*-butylphenyl rings. The simulated PXRD based on the cryoEM structure of **P2** shows a similar pattern to the experimental room temperature PXRD of **P2** except a noticeable shift of all the peaks to higher angle (Supplementary Fig. 17). Such differences are ascribed to temperature-induced lattice expansion that is well observed in crystalline polymers^64,65^, since the former is based on structures obtained at cryogenic temperature while the latter is from room temperature (RT) measurements. While we are experimentally limited to conduct PXRD measurements at cryogenic temperatures, we have verified the thermal lattice expansion behavior at above-RT conditions. Variable temperature PXRD studies conducted between room temperature and 100 ^o^C clearly indicate a consistent lattice expansion upon increasing the temperature (Supplementary Figs. 18a and 19), corroborating with the trend of PXRD peak shifts shown in Supplementary Fig. 17. The successful crystal structure determination represents an unprecedented TCP product structure determined with atomic resolution by microED, which achieves a high degree of structural accuracy despite its slightly lower precision in bond length and angles. The results highlight the advantage of electron diffraction for polymer structural determination in comparison to X-ray analysis, which requires single crystals several orders of magnitude larger, as well as single-particle analysis, which requires monodisperse but rotationally-nonuniform high molecular weight compounds^63^.

### Optical and thermal characterization of the polymerization

**Fig. 3:**
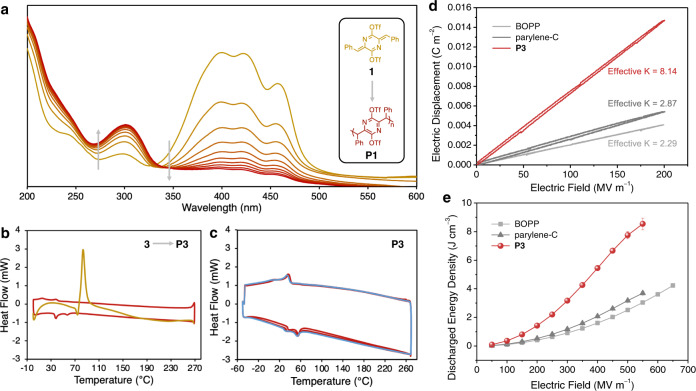
Optical and thermal interrogation of the topochemical polymerization of AQMs and capacitive energy storage properties of polymer P3. **a** UV-Vis scanning kinetic curves of a film of **1** (gold) as it polymerizes to form **P1** (ruby) over the course of roughly eight hours. **b** and **c** DSC studies of monomer **3** and the in-situ thermally polymerized **P3**. **b** the first heating (gold), and the subsequent cycle (ruby) of **3** as it polymerizes to **P3**. **c** multiple heating and cooling cycles (ruby = first four cycles, blue = final cycle) of **P3**. **d** Comparative electric displacement-electric field (*D*-*E*) loops at 200 MV m^–1^ showing the effective *K* values derived therefrom. **e** discharged energy density plots as a function of the electric field, showing the superior energy storage properties of **P3** against parylene-C and BOPP (BOPP = biaxially oriented polypropylene; error bars represent standard deviations obtained from at least three measurements using different samples).

The polymerizations of **1**–**3** are not limited to single crystals but readily occur in polycrystalline powders and thin films as well. Films of monomers **1**–**3** could be easily obtained via spin-coating and polymerize within minutes when exposed to ambient light and overnight in the dark at room temperature to yield films of **P1**–**P3**—a transformation that can be directly observed by taking UV-Vis scans at regular intervals (Fig. 3a and Supplementary Fig. 25). Visible-light triggered TCP reactions such as these are rare but preferable over higher-frequencies as visible light sources are comparatively safe and accessible^8,42^. The conversions of **1**–**3** to **P1**–**P3** are accompanied by marked shifts in their absorption and fluorescence behavior (Supplementary Figs. 23 and 24). As typified by a film of monomer **1**, upon polymerization, the three visible region absorbance subpeaks recede and one new ultraviolet absorbance appears. This shift in absorbance is consistent with the shortening of the chromophoric conjugated unit upon polymerization. During the polymerization process, no intermediate absorbances appear and then recede; instead, an isosbestic point is observed at λ = 334 nm, indicating that the polymerization occurs via a mechanism with no major intermediates or side-products. Similar features are seen in the polymerizations of **2** and **3**.

Differential scanning calorimetry (DSC) studies performed on **1**–**3** between 0 and 270 °C all show large exothermic features with their maxima centered around 100 °C on the first heating sweeps, corresponding to the thermally induced, exothermic polymerization reaction (Fig. 3b and Supplementary Fig. 8). After the first heating, the samples were fully converted to their polymeric forms. Subsequent heating and cooling cycles give featureless traces for **P1** and **P2** (Supplementary Figs. 8 and 9), showing the lack of thermal transitions under the degradation temperature (~300 °C as determined by thermogravimetric analysis, Supplementary Fig. 10). In contrast, **P3** shows two small repeatable peaks in both the heating and cooling sweeps of its DSC (Fig. 3c), although variable temperature PXRD did not show significant changes of diffraction patterns during these transitions (Supplementary Fig. 18b). It is postulated that these transitions may correspond to thermally induced reorganization of side chains though the exact reason remains undetermined.

### Dielectric capacitive energy storage properties

Polymer **P3** was anticipated to show a high dielectric constant (*K*) and other desirable properties as a polymer dielectric due to the rotatable and highly polar triflate groups on each of its repeat units^66,67^. The facile solution processability of **P3** allows for it to be recast into thin films for dielectric capacitor devices. An effective *K* of 8.14 is obtained for **P3**, which is notably higher than that of the structurally related poly-*p*-xylylene, parylene-C (*K* = 2.87), as well as the widely applied biaxially oriented polypropylene (BOPP) (*K* = 2.29) (Fig. 3d and Supplementary Fig. 27). Together with a high breakdown strength of 568 MV m^–1^ (Fig. 3e and Supplementary Fig. 28) and high charge–discharge efficiencies (>97% at 200 MV m^–1^) (Supplementary Fig. 29), the **P3**-based film capacitor delivers a high discharged energy density of 8.54 J cm^–3^. Additionally, the energy density of **P3**-based capacitors shows less than 2% variation over 50,000 consecutive charge–discharge cycles, indicating that this polymer is highly stable under high-voltage conditions (Supplementary Fig. 30). It is widely understood in the field of dielectrics that there is a fundamental tradeoff between the charge–discharge efficiency and discharged energy density of a dielectric material^66,67^. Despite this, polymer **P3**-based capacitors showed the best combination of these two metrics reported thus far under the benchmark condition of 200 MV m^–1^ (Supplementary Fig. 31).

### Postpolymerization modification

**Fig. 4:**
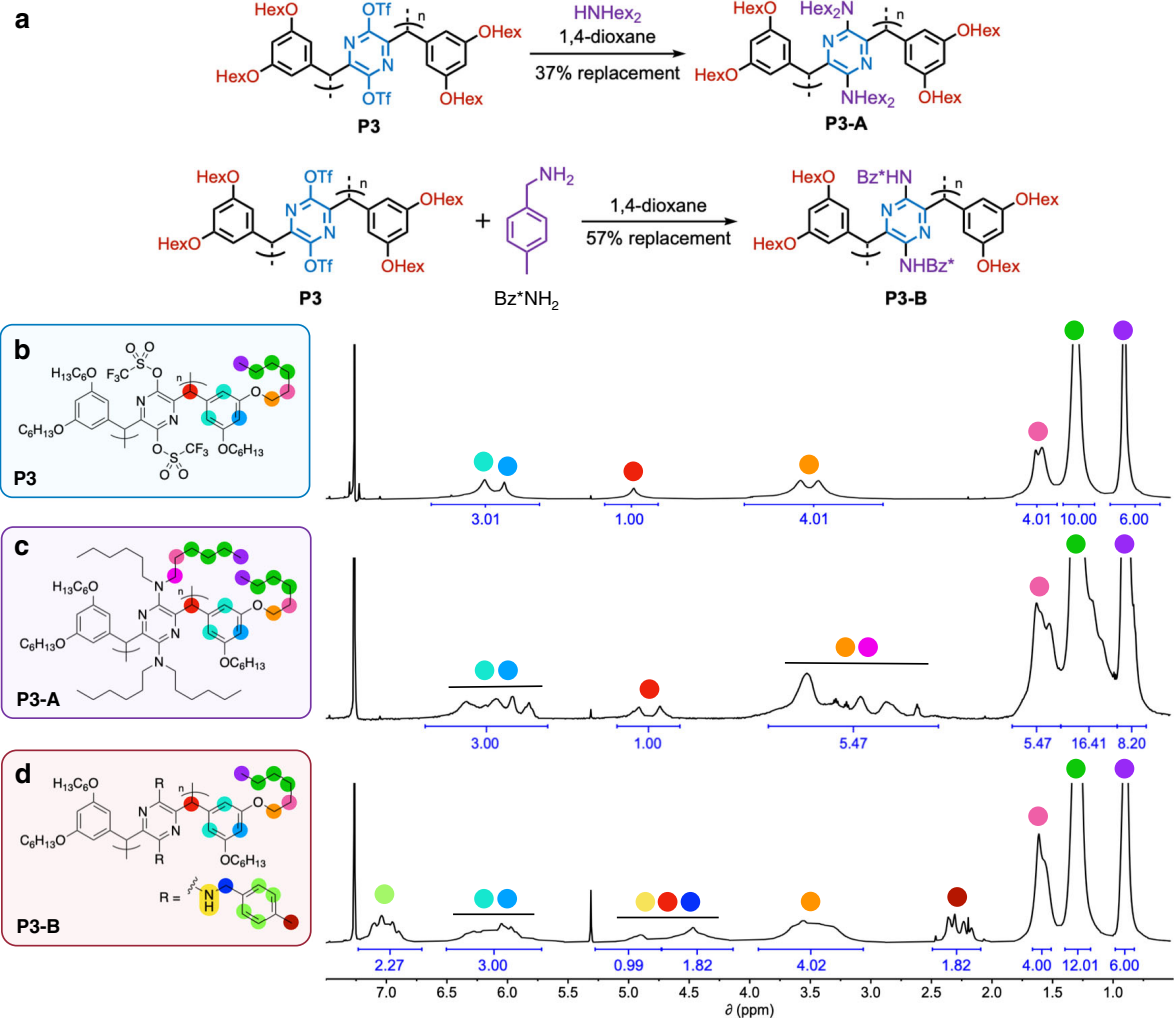
Postpolymerization functionalization of polymer P3 with amines. **a** The synthesis of two amine-functionalized polymers from reacting polymer **P3** with primary and secondary amine nucleophiles. **b–d** ^1^H NMR spectra of (**b**) polymer **P3** and its postpolymerization functionalization products, (**c**) **P3-A** and (**d**) **P3-B**, showing 37%, and 57% triflate replacement, respectively. All proton resonances are annotated by colored circles.

The triflate substituents on monomers **1**–**3** remain intact through the polymerization process, allowing for the tuning of the resultant polymeric material’s properties via postpolymerization functionalization. Due to its facile solution characterization, **P3** was chosen as a model polymer to explore the substitution reaction with the amine nucleophiles dihexylamine and 4-methylbenzylamine. The substitution reactions occurred readily to produce **P3-A** and **P3-B**, respectively, as verified by solution NMR spectroscopy (Fig. 4 and Supplementary Figs. 55–58). The ^1^H NMR spectra of **P3-A** and **P3-B** showed that 37 and 57% of the triflate groups in **P3** were displaced by dihexylamine and 4-methylbenzylamine, respectively, with their ^19^F NMR spectra showing the presence of the remaining intact triflates (Fig. 4, Supplementary Figs. 56 and 58).

## Discussion

AQM-based TCP reactions were shown to generate solution-processable UHMW polymers with excellent dielectric properties and controllable functionality. This method of synthesizing functionalized UHMW polymers exhibits notable advantages that overcome the constraints of previously reported TCP reactions, rendering it more amenable to practical use. In addition, the use of cryoEM for the structural determination of a TCP product crystal was demonstrated, obviating the need for large X-ray-quality polymer single crystals. While there is inherit restraint with regard to data collection under the employed TEM conditions, which results in relatively lower data/parameter ratio and precision for microED structures than for the X-ray structures, quantitative analysis in terms of root mean square (RMS) deviation between the electron and X-ray structures has shown a high degree of accuracy^68^ despite the experimental limitations of electron diffraction in its current state^69^.

The family of AQM ditriflates that undergo TCP is likely to be much larger than the few detailed here. Together with the demonstrated ability to undergo postpolymerization reactions, this reaction motif provides a wide access to diverse structures and functions, which facilitates future optimization of their capacitance properties. These advances allow TCP reactions to function as another practical tool in the toolbox of polymer chemists, opening up applications that were inaccessible before.

## Methods

Monomers **1**–**4** were generated analogously to a previously published two-step synthetic route^60,61^ and the synthetic details were described in detail in Supplementary Information. X-ray quality single crystals of monomers **1**–**4** were obtained via the slow evaporation of their solutions in toluene, CH_2_Cl_2_, CHCl_3_, or tetrahydrofuran in the dark. Crystals of monomers **1**–**3** were polymerized over roughly three days on a windowsill that receives regular sunlight. TEM grids were decorated with microcrystals of polymer **P2** for cryoEM by dipping them in 10 mg mL^–1^ solutions of **2**, allowing them to air dry, and then polymerizing in an analogous way to the larger crystals. Polymer **P3** was functionalized with primary and secondary amines by stirring a solution of the polymer in 1,4-dioxane to 100 °C for 5 days with 10 equivalents of the amine per repeat unit of the polymer. The functionalized polymers **P3-A** and **P3-B** were isolated via precipitation with methanol, followed by filtration and purified by washing the precipitate with different solvents. Polymer film capacitors were generated using ~3 μm-thick films of polymer **P3** drop-cast on ITO-coated glass substrates from a 6.7 mg mL^–1^ solution in tetrahydrofuran. After thermally treating the films at 105 °C for 24 h, 30 nm gold electrodes were deposited on the top of the films. The dielectric and capacitive properties were measured using a PK-CPE1801 high-voltage test system containing a Trek 610D amplifier (PolyK Technologies, LLC, USA).

## Supplementary information

Supplementary Information

Description of Additional Supplementary Files

Supplementary Movie 1

## Data Availability

The data that support the findings of this study are available from the corresponding author upon request. The crystallographic data of monomers **1–4** and **P2** generated in this study have been deposited to Cambridge Structural Database under accession code 2102275, 2102276, 2102277, 2102278, and 2102279, respectively.
