# Supplementary material for: Solution-processable and functionalizable ultra-high molecular weight polymers via topochemical synthesis
Source: Nat Commun. 2021 Nov 24;12:6818. doi: 10.1038/s41467-021-27090-1 (PMC8613210; doi:10.1038/s41467-021-27090-1)
Supplement: Supplementary file 1 — Supplementary Information [file 41467_2021_27090_MOESM1_ESM.pdf]

Supplementary Information

for

Solution-Processable and Functionalizable Ultra-high Molecular Weight Polymers via Topochemical Polymerization

Christopher L. Anderson[1,2], He Li[1,3†], Christopher G. Jones[4†], Simon J. Teat[5], Nicholas S. Settineri[2,5], Eric A. Dailing[1], Jiatao Liang[1,2], Haiyan Mao[6], Chongqing Yang[1], Liana M. Klivansky[1], Xinle Li[1], Jeffrey A. Reimer[6], Hosea M. Nelson*[4], Yi Liu*[1,3]

[1]The Molecular Foundry, Lawrence Berkeley National Laboratory, One Cyclotron Road, Berkeley, California 94720, United States

[2]Department of Chemistry, University of California, Berkeley, Berkeley, California 94720, United States

[3]Materials Sciences Division, Lawrence Berkeley National Laboratory, One Cyclotron Road, Berkeley, California 94720, United States

[4]Department of Chemistry and Biochemistry, University of California, Los Angeles, Los Angeles, California 90095, United States

[5]Advanced Light Source, Lawrence Berkeley National Laboratory, One Cyclotron Road, Berkeley, California 94720, United States

[6]Department of Chemical and Biomolecular Engineering, University of California, Berkeley, Berkeley, California 94720, United States

[†]These authors contributed equally

Corresponding Author: Yi Liu, E-mail: yliu@lbl.gov
The Molecular Foundry, One Cyclotron Rd, Lawrence Berkeley National Laboratory, Berkeley, CA 94720

Corresponding Author: Hosea M. Nelson, E-mail: hosea@chem.ucla.edu
Department of Chemistry and Biochemistry, University of California, Los Angeles, Los Angeles, California 90095, United States

Page	Contents
3 - 16	Supplementary Methods: General, X-ray Crystallography, Dielectric Measurements, Synthesis
5	Figure 1: Schematic Representation of the Preparation of Polymer **P2** Microcrystals for CryoEM
8	Figure 2: The Synthesis of Monomers **1**-**4** and Polymers **P1**-**P3**
17	Figure 3: Optical Microscope Images of Monomers **1**-**4** and Polymers **P1**-**P3**
17 - 18	Figure 4: Optical Microscope Images of Monomer **1** Crystallized from Different Solvents
19	Figure 5: Infrared Spectroscopy Fingerprint Regions of **1** and **P1** along with a Computed Vibrational Mode of **1**
20	Figure 6: Infrared Spectroscopy of Monomers **1**-**3** and Polymers **P1**-**P3**
21	Figure 7: Size-exclusion Chromatography Data on Polymer **P1** Generated from Different Solvents
22	Figure 8: Differential Scanning Calorimetry of Polymers **P1**-**P3**
23	Figure 9: Differential Scanning Calorimetry of **P3-A** and **P3-B**
23	Figure 10: Thermogravimetric Analysis of Monomers **1**-**3** and Polymers **P1**-**P3**
24	Figure 11: Powder X-ray Diffraction (PXRD) of Monomers **1**-**3** and Polymers **P1**-**P3**
24	Figure 12: X-ray Crystallographic Structures of Monomer **1**
25	Figure 13: X-ray Crystallographic Structures of Monomer **2**
25	Figure 14: X-ray Crystallographic Structures of Monomer **3**
26	Figure 15: X-ray Crystallographic Structures of Monomer **4**
26	Figure 16: X-ray Crystallographic Structure of Monomer **2** and CryoEM Structure of Polymer **P2**
27	Figure 17: Experimental and Predicted PXRD patterns of Monomer **2** and Polymer **P2**
28	Figure 18: Variable Temperature PXRD of polymers **P2** and **P3** between 25 and 100 ºC
28	Figure 19: Correlation between 100 peak maximum value and temperature in polymer **P2**
29	Table 1: Numerical Data from X-ray Crystallography of Monomers **1**-**4** and CryoEM of Polymer **P2**
30	Figure 20: Scanning Electron Microscopy (SEM) Images of Polymer **P2** Crystals on TEM grids
31	Figure 21: Tunnelling Electron Microscopy (TEM) Images and Electron Diffraction Patterns of Polymer **P2** Crystals
32	Figure 22: CryoEM Structure of Polymer **P2** Showing 3D Electron Density
33	Figure 23: UV-Visible Spectroscopy of Monomers **1**-**3** and Polymers **P1**-**P3**
34	Figure 24: UV-Visible and Fluorescence Spectroscopy of Monomers **1**-**3** and Polymers **P1**-**P3**
35	Figure 25: UV-Visible Scanning Kinetics Traces of Monomers **1**-**3** Converting into Polymers **P1**-**P3**
36	Figure 26: Infrared Spectroscopy of Functionalized Polymers **P3-A** and **P3-B**

Page	Contents
37	Figure 27: Electric Displacement–Electric Field (*D*–*E*) Loops of Polymer **P3** and Other Dielectric Polymers
38	Figure 28: Weibull Plots of Dielectric Breakdown Strength of Polymer **P3** and Other Dielectric Polymers
39	Figure 29: Discharged Energy Density and Charge–Discharge Efficiency Plots of Polymer **P3** and Other Polymers
40	Figure 30: Cyclability Studies on Polymer **P3**-based Thin Film Capacitors
42	Figure 31: Comparison of Polymer **P3** with Other Dielectric Polymers
43 - 59	Figures 32 – 59: Nuclear Magnetic Resonance (NMR) Spectra of All Herein Reported Compounds
60	Supplementary References

Supplementary Methods

General

All reactions were carried out in oven-dried glassware sealed with rubber septa under an atmosphere of nitrogen unless otherwise noted and were stirred using Teflon-coated magnetic stir bars. Large volumes of volatile solvents were removed using rotary evaporation, and small volumes of volatile solvents were removed using nitrogen gas flow. All commercially available chemicals were purchased from Alfa Aesar, Spectrum Chemicals, Acros Organics, TCI America, or Sigma-Aldrich, and were used without further purification. Deuterated solvents were purchased from Cambridge Isotope Laboratories and used as received. Solid-state ^{13}C NMR spectra were carried out at 500.12 MHz (11.7 T) on a Bruker Avance spectrometer with a Bruker 4 mm narrow bore H/C/N magic angle spinning probe. Solid state ^{13}C NMR spectra were in general acquired by cross-polarization from ^{1}H with a contact time of 5 ms at a spinning rate of 10 kHz. All solution NMR spectra were recorded at 298 K on a Bruker 500 MHz Avance instrument unless otherwise specified. All chemical shifts are quoted using the δ scale, and all coupling constants (J) are expressed in Hertz (Hz). Solution ^{1}H and ^{13}C NMR spectra chemical shifts are reported relative to the residual solvent signal (^{1}H NMR: $CDCl_3$ δ = 7.26 ppm, acetone-d_6 δ = 2.05 ppm, THF-d_8 δ = 1.72/3.58 ppm, and DMSO-d_6 δ = 2.50 ppm; ^{13}C NMR: $CDCl_3$ δ = 77.16 ppm, acetone-d_6 δ = 29.84/206.26 ppm, THF-d_8 δ = 67.21/25.31 ppm, and DMSO-d_6 δ = 39.52 ppm). NMR data are reported as follows: chemical shift (multiplicity, coupling constants where applicable, number of hydrogens where applicable). Splitting is reported with the following symbols: s = singlet, d = doublet, t = triplet, dd = doublet of doublets, td = triplet of doublets, ddd = doublet of doublet of doublets, sept = septet, m = multiplet, bs = broad singlet. MALDI-TOF mass spectrometry experiments were performed on an Applied Biosystems 4800 MALDI TOF/TOF instrument in reflector mode using super-DHB (purchased from Sigma-Aldrich) as matrix. UV-Vis-NIR

spectra were recorded using a Cary 5000 UV-Vis-NIR spectrometer. Films for UV-Vis spectroscopy were prepared via spin-coating 100 µL of 10 mg mL^{-1} solutions on substrates spinning at 1000 rpm for 40 seconds protected from significant exposure to light. Solution UV-Vis spectra were taken in dilute solutions in dichloromethane or in films on quartz substrates, and optical band gaps were determined based on the onset of the highest-wavelength absorbance feature. Scanning electron microscope (SEM) images were obtained using a ZEISS Ultra-55 field emission scanning electron microscope (FESEM). Size exclusion chromatography (SEC) was performed on a Malvern OMNISEC equipped with refractive index, light scattering, and intrinsic viscosity detectors calibrated with a single poly(styrene) standard. Analysis was performed in tetrahydrofuran running at 1 mL min^{-1} and 35 ºC. Computations were run on the Etna partition of the Lawrencium Supercluster at Lawrence Berkeley National Labs using Qchem 5.1.[1] Molecular geometries were optimized starting from structures generated in the IQmol software and relaxed using the UFF molecular mechanics force field.[2] Molecular geometries were then optimized and single-point energies were determined at the 6-311++G** level using the B3LYP functional. Orbital density maps were visualized using the IQmol software with an isovalue of 0.02.

X-ray Crystallography

Monomers **1**, **2**, and **4** were crystallized via slow evaporation from toluene, and monomer **3** was crystallized via slow evaporation from chloroform (see crystallization section below for details). After 1-3 weeks undisturbed, all of the monomers had formed yellow needle-like crystals roughly 5 mm in size of suitable quality to obtain x-ray structures. Single crystals of **1**-**4** were selected and mounted on Mitegen® loops with Paratone oil, and data were collected on beamline 12.2.1 at the Advanced Light Source with λ = 0.7749 Å at 100 K using an Oxford Cryosystems Cryostream 800 plus. The x-ray structures of monomers **1**-**4** were collected on a Bruker D8 diffractometer with a Bruker PHOTON100 CMOS detector. Data reduction was performed and corrected for Lorentz and polarization effects using SAINT[3] v8.38a and were corrected for absorption effects using SADABS v2016/2.[4] Structures were solved using SHELXT[4] and were refined by least-square refinement against F^2 by SHELXL.[5]

Electron Cryomicroscopy (CryoEM)

Microcrystals of polymer **P2** were deposited on TEM grids by preparing 10 mg mL^{-1} solutions of monomer **2** in chloroform, tetrahydrofuran, and dichloromethane. TEM grids were dipped into the solutions, removed, and allowed to air dry for a few seconds (Supplementary Figure 1). Afterwards, the TEM grids were placed in clean vials, and the vials were placed on a windowsill that receives direct sunlight. After three days on the windowsill, the polymerization was complete.

Coated TEM grids were loaded in a Gatan 626 single-tilt cryo holder and cooled with liquid nitrogen. Screening and data collection was performed using a Thermo-Fisher F200C transmission

electron microscope operating at 200 kV and equipped with a Thermo-Fisher Ceta-D detector. Screening was done in low-dose mode and diffraction was identified through condensing of the electron beam. After selection of a crystal on the grid, the crystal was centered, the eucentric height adjusted by tilting the crystal through the desired rotation range, and the selected area aperture and beam stop were inserted.[6]

Images were collected in a movie format as crystals were continuously rotated in the electron beam. Typical data collection was performed using a constant tilt rate of 0.3°/s between the minimum and maximum tilt ranges of -72° to +72°, respectively. During continuous rotation, the camera integrated frames continuously at a rate of 3 seconds per frame. The dose rate was calibrated to <0.03 e-/Å^2 s. Crystals selected for data collection were isolated by a selected area aperture to reduce the background noise contributions and calibrated to eucentric height to stay in the aperture over the entire tilt range. All diffraction data were processed using the XDS suite of programs.[7] The resulting crystal structure was solved ab initio using direct methods in SHELXT[4] and refined with SHELXL[8] using ShelXle.[9] Structure refinement was performed using electron scattering factors reported by Peng.[10] Thermal parameters were refined anisotropically for all non-hydrogen atoms.

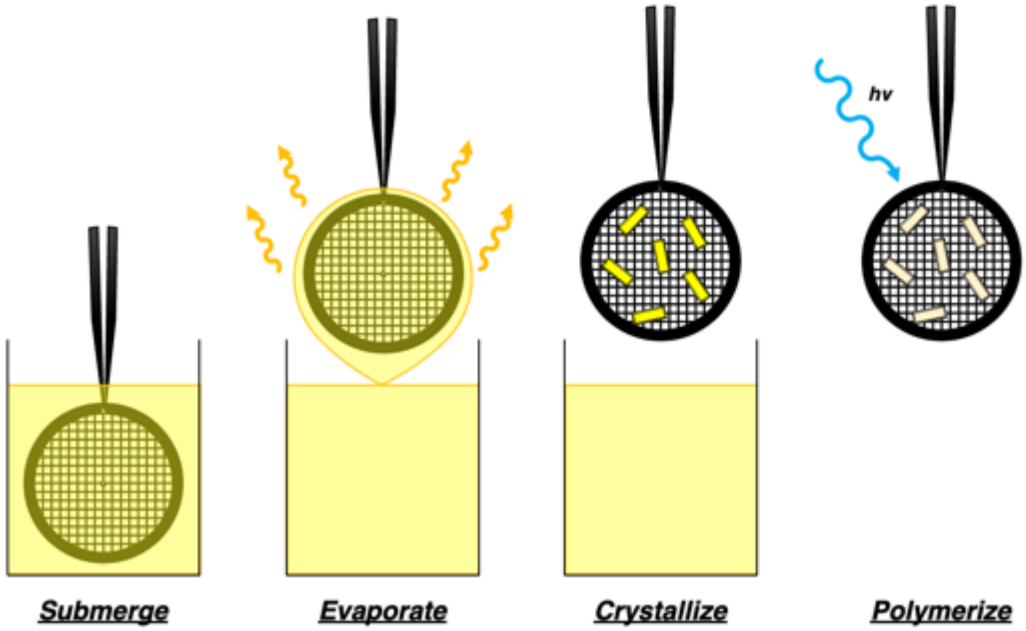

Supplementary Figure 1. A schematic representation of the preparation of polymer **P2** microcrystals on TEM grids for cryoEM.

Dielectric Measurements

Indium tin oxide (ITO) coated glass substrates (2-3 Ω sq^{-1}, Thin Film Devices Inc., USA) were pre-cleaned using soapy water, deionized (DI) water, acetone, and isopropanol, sequentially. The substrates were then heated at 100 ºC for at least four hours, followed by ultraviolet/ozone (UV/O_3) treatment for 20 min prior to use.

Parylene-C Films

A ~1.5 µm thick layer of Parylene-C was deposited on ITO-coated glass substrates using chemical vapor deposition (PDS 2010 Labcoter, SCS) as follows. Parylene-C Dimer (2 g) was placed in the sublimation chamber, which was heated to 175 ºC under high vacuum. The dimer vapor was cleaved to monomer vapor at 690 ºC and flowed into the unheated deposition chamber, which contained the ITO coated glass substrates. The thickness was checked using a Dektak 3030ST profilometer to be between 1.4 µm and 1.6 µm. A gold electrode (4 mm^2 area with 30 nm thickness) was deposited on the top surface of the polymer film using a thermal evaporator (MBRAUN, Germany) for dielectric characterization.

Polymer P3 Films

Polymer **P3** (20 mg) was dissolved in 3 mL tetrahydrofuran (THF) and magnetically stirred for 24 h to yield a transparent solution. Afterwards the solution was drop-cast on ITO substrates and they were placed in a covered petri dish to slow down the solvent evaporation process. The coated substrates were then thermally treated at 105 ºC for 24 h in a vacuum oven to remove solvent residual. The thickness was checked using a Dektak 150 profilometer to be between 2.6 µm and 4.4 µm. A gold electrode (4 mm^2 area with 30 nm thickness) was deposited on the top surface of the polymer film using a thermal evaporator (MBRAUN, Germany) for dielectric characterization.

Other Dielectric Polymer Films

Free-standing polymer films of biaxially-oriented polypropylene (BOPP, capacitor grade, ~3.8 µm) ethylene tetrafluoroethylene (ETFE, ~12 µm), polycarbonate (PC, ~10 µm), poly(ethylene 2,6-naphthalate (PEN, ~8 µm), polyimide (PI, Upilex-S, ~8.8 µm), poly(ether imide) (PEI, ~12 µm), poly(vinylidene fluoride) (PVDF, 8 µm), and Poly(vinylidene fluoride-*co*-hexafluoropropylene) (PVDF-HFP, 10 wt% HFP, 5 µm) were obtained from PolyK Technologies, LLC, USA. Gold electrodes (4 mm^2 area with 30 nm thickness) were deposited on both sides of the dielectric polymer films using a thermal evaporator (MBRAUN, Germany) for dielectric characterization.

Polystyrene (PS, Sigma Aldrich, 280,000 molecular weight (SEC)) and poly(vinylidene fluoride-*co*-chlorotrifluoroethylene) (PVDF-CTFE, 15 wt% CTFE, PolyK Technologies) polymer powders (60 mg) were each dissolved in 3 mL dimethyl formamide (DMF), and magnetically stirred for 24 h to yield

clear solutions. Afterwards the solution was drop-cast onto ITO substrates in an air-circulating oven at 65 °C, followed by a thermal treatment step at 105 °C for 24 h in a vacuum oven to remove residual solvent. The thickness of the cast films was controlled between 2.5 μm and 5 μm. A gold electrode (4 mm^2 area with 30 nm thickness) was deposited on the top surface of the polymer film using a thermal evaporator (MBRAUN, Germany) for dielectric characterization.

To avoid creeping discharge, the samples for all of the dielectric measurements were immersed in a Galden HT-270 PFPE fluorinated fluid. Electric displacement–electric field (*D*–*E*) loops were collected under varied applied electric fields using a PK-CPE1801 high-voltage test system containing a high voltage amplifier (Trek 610D, PolyK Technologies, LLC, USA). The voltages with a unipolar triangular waveform were applied at a frequency of 100 Hz. The electric field of 200 MV m^{-1} was chosen as it is the field of interest for power inverters in hybrid electric vehicles.[11,12] The charge–discharge cyclic tests were performed under a repeatedly applied electric field of 200 MV m^{-1} using on the same high-voltage test system using the Fatigue mode. Discharged energy density (U_d) and charge–discharge efficiency (η) were calculated from the *D*–*E* loops using the method illustrated in Supplementary Figure 27. Dielectric breakdown strength measurements were performed using the same Trek 610D high voltage amplifier using the electrostatic pull-down method, where a DC voltage ramp of 200 V s^{-1} was applied to the polymer film samples until dielectric failure.

Synthesis

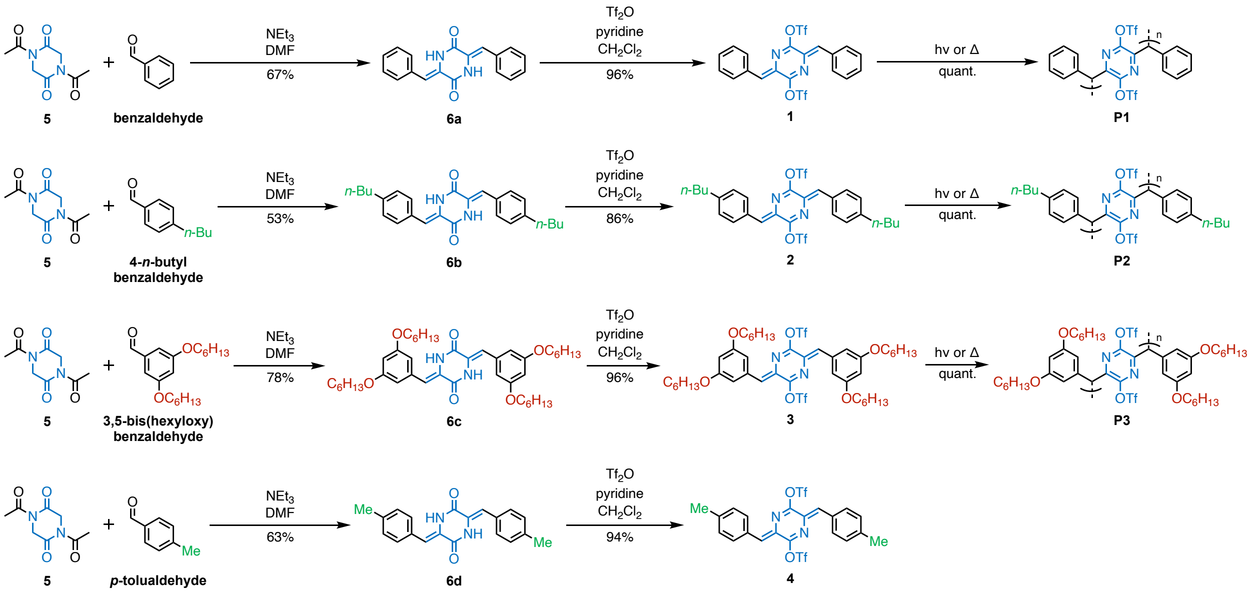

Supplementary Figure 2. The syntheses of monomers **1-4** and polymers **P1**-**P3**.

1,4-Diacetylpiperazine-2,5-ione (**5**)

1,4-Diacetylpiperazine-2,5-dione was synthesized from glycine anhydride according to a previously reported procedure.[13]

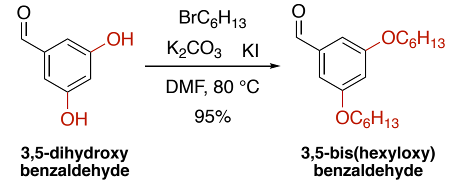

3,5-bis(hexyloxy)benzaldehyde

A 250 mL round-bottom flask with a stir bar and air-condenser was charged with 3,5-dihydroxybenzaldehyde (1.00 g, 7.24 mmol) and dry DMF (100 mL). Potassium carbonate (2.50 g, 18.10 mmol), potassium iodide (120.19 mg, 724.00 mmol), and 1-bromohexane (2.24 mL, 15.93 mmol) were added. The mixture was heated to 80 °C and continually stirred for 15 hrs at this temperature. After cooling to room temperature, the solvent was removed via rotary evaporation. The mixture was subjected to column chromatography (hexanes to ethyl acetate gradient) to yield analytically pure 3,5-bis(hexyloxy)benzaldehyde as a very faintly brown liquid (95% yield). ^{1}H NMR (500 MHz, $CDCl_3$) δ 9.88 (s, 1H), 6.98 (d, J = 2.4 Hz, 2H), 6.69 (t, J = 2.3 Hz, 1H), 3.97 (t, J = 6.6 Hz, 4H), 1.80 (dt, J = 15.3, 6.7 Hz, 4H), 1.49 – 1.42 (m, 4H), 1.36 – 1.32 (m, 8H), 0.91 (t, J = 6.9 Hz, 6H). ^{13}C NMR (126

MHz, $CDCl_3$) δ 192.08, 160.70, 138.25, 107.88, 107.48, 68.35, 31.57, 29.10, 25.70, 22.67, 14.07. MS (ESI) for $C_{19}H_{30}O_3$ $[M+1]^+$: calcd., 307.2195; found, 307.2338.

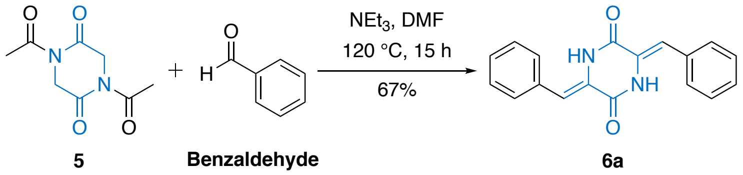

Compound **6a**

1,4-Diacetylpiperazine-2,5-dione (**5**) (5.00 g, 25.23 mmol), triethylamine (14.08 mL, 100.92 mmol), and benzaldehyde (5.46 mL, 63.08 mmol) were dissolved in DMF (50 mL), and the resulting solution was stirred in a 120 °C sand bath for 15 h. The reaction mixture was then cooled to room temperature and diluted with water (40 mL). The precipitated solid was filtered and washed with roughly 10 mL each of water, ethyl acetate, and finally methanol to afford **6a** as a light-yellow powder (67%). ^1H NMR (500 MHz, DMSO-d_6) δ 10.31 (s, 1H), 7.55 (d, J = 7.3 Hz, 2H), 7.42 (t, J = 7.6 Hz, 2H), 7.33 (t, J = 7.4 Hz, 1H), 6.78 (s, 1H). ^{13}C NMR (126 MHz, DMSO-d_6) δ 158.04, 133.11, 129.37, 128.72, 128.23, 126.39, 115.06. MS (MALDI-TOF) for $C_{18}H_{14}N_2O_2$ $[M]^+$: calcd., 290.1055; found, 290.1117.

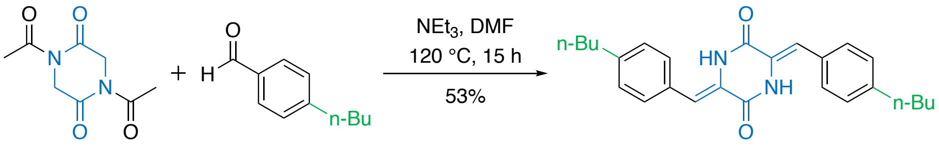

Compound **6b**

1,4-Diacetylpiperazine-2,5-dione (**5**) (596.00 mg, 3.01 mmol), triethylamine (1.68 mL, 12.03 mmol), and 4-*n*-butyl-benzaldehyde (1.03 mL, 6.16 mmol) were dissolved in DMF (6 mL), and the resulting solution was stirred in a 120 °C sand bath for 15 h. The reaction mixture was then cooled to room temperature and diluted with water (10 mL). The precipitated solid was filtered and washed with roughly 10 mL each of water, ethyl acetate, and finally methanol to afford **6b** as a light-yellow powder (53%). ^{1}H NMR (500 MHz, DMSO-d_6) δ 9.64 (s, 1H), 7.46 (d, J = 7.9 Hz, 2H), 7.27 (d, J = 7.8 Hz, 2H), 6.79 (s, 1H), 2.64 (t, J = 7.6 Hz, 2H), 1.62 (p, J = 7.2 Hz, 2H), 1.37 (h, J = 7.3 Hz, 2H), 0.94 (t, J = 7.3 Hz, 4H). ^{13}C NMR (126 MHz, DMSO-d_6) δ 158.16, 143.29, 131.03, 129.56, 129.18, 126.38, 115.48, 35.09, 33.12, 22.07, 13.97. MS (ESI) for $C_{26}H_{30}N_2O_2$ [M+1]$^+$: calcd., 403.2307; found, 403.2433.

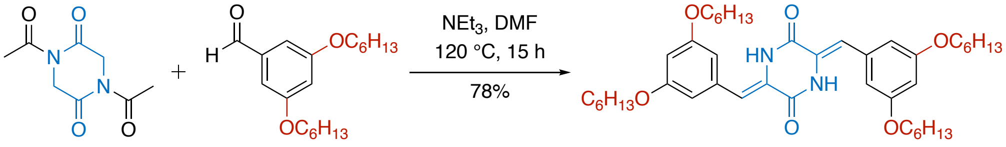

Compound **6c**

1,4-Diacetylpiperazine-2,5-dione (**5**) (100.00 mg, 504.60 µmol), triethylamine (281.53 µL, 2.02 mmol), and 3,5-bis(hexyloxy)benzaldehyde (324.73 mg, 1.06 mmol) were dissolved in DMF (1 mL), and the resulting solution was stirred in a 120 °C sand bath for 15 h. The reaction mixture was then cooled to room temperature and diluted with water (5 mL). The precipitated solid was filtered and washed with roughly 10 mL each of water and methanol (Note: do not wash with ethyl acetate) to afford **6c** as a light-yellow powder (78%). ^{1}H NMR (500 MHz, $CDCl_3$) δ 8.28 (s, 1H), 6.96 (s, 1H), 6.49 (dd, J = 2.3, 0.6 Hz, 2H), 6.46 (t, J = 2.2 Hz, 1H), 3.96 (t, J = 6.6 Hz, 4H), 1.80 (dt, J = 15.3, 6.6 Hz, 4H), 1.53 – 1.43 (m, 4H), 1.43 – 1.31 (m, 8H), 0.94 (t, J = 7.0 Hz, 6H). ^{13}C NMR (126 MHz, $CDCl_3$) δ 161.10, 156.81, 125.76, 116.97, 106.62, 101.53, 68.24, 31.60, 29.17, 25.74, 22.64, 14.11. MS (ESI) for $C_{42}H_{62}N_2O_6$ [M+1]$^+$: calcd., 691.4608; found, 691.4719.

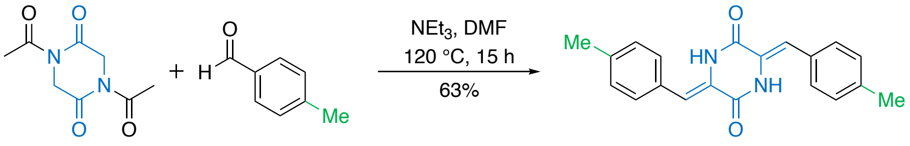

5 *p*-**tolualdehyde** **6d**

Compound **6d**

1,4-Diacetylpiperazine-2,5-dione (**5**) (804.59 mg, 4.06 mmol), triethylamine (2.27 mL, 16.24 mmol), and *p*-tolualdehyde (981.35 µL, 8.32 mmol) were dissolved in DMF (8 mL), and the resulting solution was stirred in a 120 °C sand bath for 15 h. The reaction mixture was then cooled to room temperature and diluted with water (10 mL). The precipitated solid was filtered and washed with roughly 10 mL each of water, ethyl acetate, and finally methanol to afford **6d** as a light-yellow powder (63%). ^{1}H NMR (500 MHz, DMSO-d$_6$) δ 9.67 (s, 1H), 7.44 (d, *J* = 8.2 Hz, 2H), 7.25 (d, *J* = 7.8 Hz, 2H), 6.77 (s, 1H), 2.35 (s, 3H). ^{13}C NMR (126 MHz, DMSO-d$_6$) δ 157.24, 137.43, 129.85, 128.93, 128.61, 125.42, 114.54, 20.32. MS (ESI) for $C_{20}H_{18}N_2O_2$ [M+1]$^+$: calcd., 319.1368; found, 319.1506.

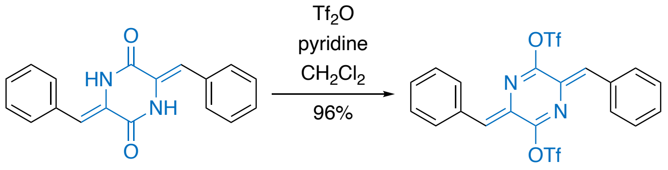

6a **1**

Monomer **1**

Trifluoromethanesulfonic anhydride (607.4 µL, 3.62 mmol) was added dropwise to a solution of **6a** (500.00 mg, 1.72 mmol) and pyridine (3.62 mmol, 291.36 µL) in dichloromethane (10 mL) at -78 °C (dry ice/acetone bath). The reaction was stirred at -78 °C for 30 mins and then slowly warmed to room temperature and stirred over 16 hrs. Hexanes was added to the crude reaction mixture, the precipitate was filtered, and the solid was washed with roughly 10 mL each of hexanes, water, and finally methanol to give analytically pure **1** as yellow needles (96%). Note: the product should be protected from ambient light and heating above 40 °C as much as possible. It is advisable to store the product in a refrigerator or freezer within aluminum-foil wrapped and/or darkened glass vessels. ^{1}H NMR (500 MHz, $CDCl_3$) δ 8.06 – 7.99 (m, 2H), 7.47 – 7.42 (m, 3H), 6.98 (s, 1H). ^{13}C NMR (126 MHz, $CDCl_3$) δ 151.51, 133.10, 132.69, 131.62, 131.58, 128.98, 126.22, 119.80, 117.24. MS (ESI) for $C_{20}H_{12}F_6N_2O_6S_2$ [M+1]$^+$: calcd., 555.0041; found, 555.0188.

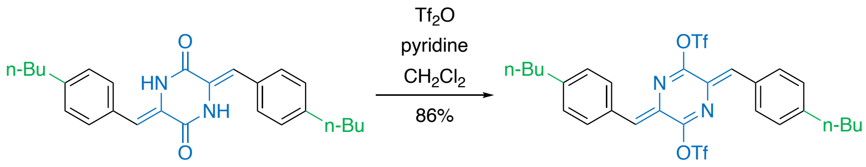

6b **2**

Monomer **2**

Trifluoromethanesulfonic anhydride (299.87 µL, 1.78 mmol) was added dropwise to a solution of **6b** (350.00 mg, 869.48 µmol) and pyridine (147.10 µL, 1.83 mmol) in dichloromethane (14 mL) at -78 °C (dry ice/acetone bath). The reaction was stirred at -78 °C for 30 mins and then slowly warmed to room temperature and stirred over 16 h. Hexanes was added to the crude reaction mixture, the precipitate was filtered, and the solid was washed with roughly 10 mL each of hexanes, water, and finally methanol to give analytically pure **2** as yellow needles (86%). Note: the product should be protected from ambient light and heating above 40 °C as much as possible. It is advisable to store the product in a refrigerator or freezer within aluminum-foil wrapped and/or darkened glass vessels. ^{1}H NMR (500 MHz, $CDCl_3$) δ 7.98 (d, *J* = 8.4 Hz, 2H), 7.30 (d, *J* = 8.5 Hz, 2H), 6.95 (s, 1H), 2.68 (t, *J* = 7.7 Hz, 2H), 1.70 – 1.60 (m, 2H), 1.39 (h, *J* = 7.4 Hz, 2H), 0.96 (t, *J* = 7.3 Hz, 3H). ^{13}C NMR (126 MHz, $CDCl_3$) δ 151.16, 147.35, 132.58, 130.89, 129.65, 129.04, 125.48, 119.65, 117.10, 35.84, 33.25, 22.41, 13.96. MS (MALDI-TOF) for $C_{28}H_{28}F_6N_2O_6S_2$ $[M]^+$: calcd., 666.1293; found, 666.1204.

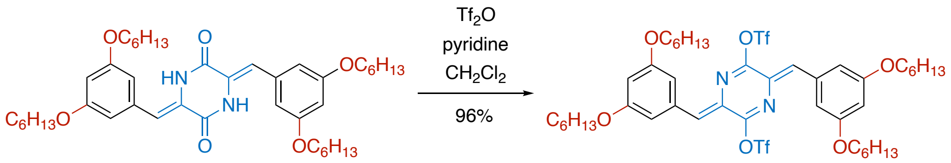

6c **3**

Monomer **3**

1M Trifluoromethanesulfonic anhydride solution in DCM (2.97 mL, 2.97 mmol) was added dropwise to a solution of **6c** (1.00 g, 1.45 mmol) and pyridine (244.84 µL, 3.04 mmol) in dichloromethane (50 mL) at -78 °C (dry ice/acetone bath). The reaction was stirred at -78 °C for 30 mins and then slowly warmed to room temperature and stirred over 16 h. Hexanes was added to the crude reaction mixture, the precipitate was filtered, and the solid was washed with roughly 20 mL each of hexanes, water, and finally methanol to give analytically pure **3** as yellow needles (96%). Note: the product should be protected from ambient light and heating above 40 °C as much as possible. It is advisable to store the product in a refrigerator or freezer within aluminum-foil wrapped and/or brown glass vessels. ^{1}H NMR (500 MHz, $CDCl_3$) δ 7.17 (d, *J* = 2.1 Hz, 2H), 6.85 (s, 1H), 6.59 (t, *J* = 2.2 Hz, 1H), 4.01 (t, *J* = 6.6 Hz, 4H), 1.80 (p, *J* = 6.9 Hz, 4H), 1.52 – 1.45 (m, 4H), 1.41 – 1.33 (m, 8H), 0.93

(t, J = 7.0 Hz, 6H). ^{13}C NMR (126 MHz, $CDCl_3$) δ 160.35, 151.12, 134.41, 131.65, 125.67, 110.62, 105.84, 68.42, 31.60, 29.24, 25.64, 22.64, 14.07. MS (ESI) for $C_{44}H_{60}F_6N_2O_{10}S_2$ $[M+1]^+$: calcd., 955.3594; found, 955.3640.

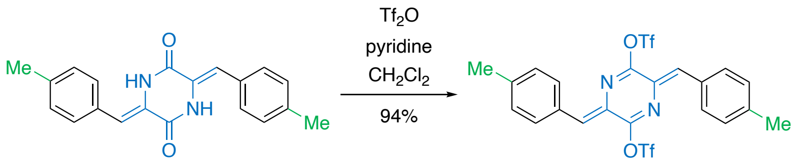

6d **4**

Monomer **4**

Trifluoromethanesulfonic anhydride (neat, 711.69 µL, 4.23 mmol) was added dropwise to a solution of **6d** (657.00 mg, 2.06 mmol) and pyridine (349.11 µL, 4.33 mmol) in dichloromethane (30 mL) at -78 °C (dry ice/acetone bath). The reaction was stirred at -78 °C for 30 mins and then slowly warmed to room temperature and stirred over 16 h. Hexanes was added to the crude reaction mixture, the precipitate was filtered, and the solid was washed with roughly 10 mL each of hexanes, water, and finally methanol to give analytically pure **4** as yellow needles (94%). ^1H NMR (500 MHz, $CDCl_3$) δ 7.93 (d, J = 8.1 Hz, 2H), 7.23 (d, J = 11.7 Hz, 2H), 6.92 (s, 1H), 1.25 (s, 3H). ^{13}C NMR (126 MHz, $CDCl_3$) δ 151.29, 142.49, 132.65, 131.02, 130.59, 129.82, 125.60, 21.92. MS (ESI) for $C_{22}H_{16}F_6N_2O_6S_2$ $[M+1]^+$: calcd., 583.0354; found, 583.0500.

Crystallizations of Monomers **1-4**

Nearly all crystallization conditions attempted on monomers **1-4** presented herein formed similar long aspect-ratio needle or hair-like crystals (Figure 1, Supplementary Figures 3 and 4). This consistency presents a trade-off, in that it both allows for flexibility in the chosen method of crystallization, while also restricting the morphology of the obtained crystal to a single type. A typical slow evaporation crystallization is detailed as follows. A small amount of the monomer compound was dissolved in a solvent (most commonly CH_2Cl_2, $CHCl_3$, tetrahydrofuran, or toluene) or combination thereof and filtered through a syringe filter into an aluminum foil-wrapped vial. The vial cap was slightly unscrewed and the solvent was allowed to evaporate over the course of days to weeks in the dark and protected from excessive vibrations. In general, number-averaged molecular weights and crystallinity in the polymer crystals produced from these monomer crystals both increased with higher boiling-point solvents.

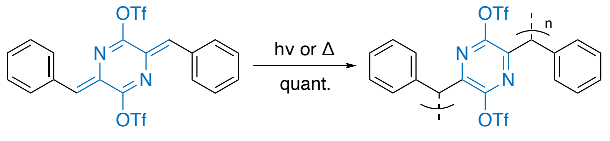

1 **P1**

Polymer **P1**

After generating crystals of **1** via the method detailed above, the vial of crystals was sparged with N_2, recapped, the aluminum foil wrapping was removed, and the vial was placed on a sunny windowsill or under a controlled light source. After roughly three days, the crystals had converted from yellow to transparent-white, indicating the conversion to **P1**. Solid-state (CP-MAS) ^{13}C NMR (126 MHz) δ 149.02, 146.45, 135.61, 128.95, 120.35, 117.81, 49.53.

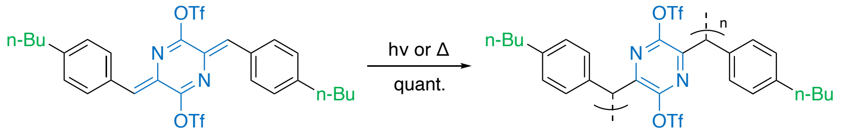

2 **P2**

Polymer **P2**

After generating crystals of **2** via the method detailed above, the vial of crystals was sparged with N_2, recapped, the aluminum foil wrapping was removed, and the vial was placed on a sunny windowsill or under a controlled light source. After the formation of crystals, the vial was sealed, the aluminum foil was removed, and the vial was placed on a sunny windowsill. After roughly three days, the crystals had converted from yellow to transparent-white, indicating the conversion to **P2**. Solid-state (CP-MAS) ^{13}C NMR (126 MHz) δ 148.82, 143.63, 133.64, 129.04, 120.16, 117.63, 52.16, 35.49, 22.74, 13.76.

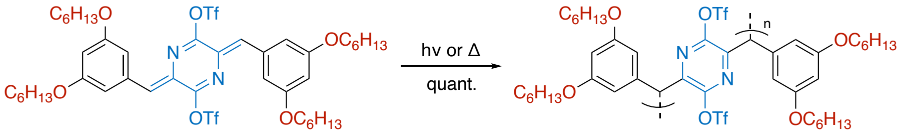

3 **P3**

Polymer **P3**

After generating crystals of **3** via the method detailed above, the vial of crystals was sparged with N_2, recappd, the aluminum foil wrapping was removed, and the vial was placed on a sunny windowsill or under a controlled light source. After roughly three days, the crystals had converted from yellow to transparent-white, indicating the conversion to **P3**. The highest molecular weight polymer crystals of **P3** were obtained via slow evaporation from toluene of **3**, with lower molecular weight distributions produced by slow evaporation from tetrahydrofuran, chloroform, and CH_2Cl_2 (Supplementary Figure

7). In order to provide a more accurate comparison to the molecular weight distributions of **P3-A** and **P3-B**, a small amount of poly(4c) was stirred in 1,4-dioxane at 100 °C for 5 days. Afterwards, the solvent was evaporated and the polymeric product was resuspended in acetone and precipitated with water. The precipitated solution was filtered, and washed with water, methanol, hexanes, diethyl ether, and acetone before being washed through with dichloromethane (78% recovery). ^{1}H NMR (500 MHz, $CDCl_3$) δ 6.60 – 5.80 (m, 3H), 4.95 (s, 1H), 3.94 – 2.99 (m, 4H), 1.78 – 1.50 (m, 4H), 1.28 (s, 12H), 0.88 (s, 6H). ^{13}C NMR (126 MHz, $CDCl_3$) δ 160.65, 148.24, 143.55, 137.39, 119.65, 117.10, 105.64, 102.64, 67.74, 49.63, 31.70, 29.28, 25.63, 22.74, 14.10.

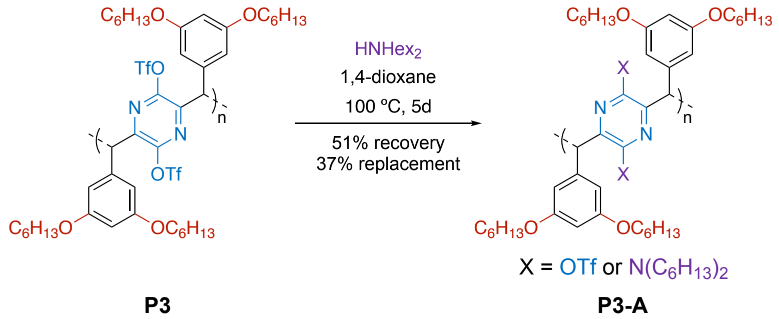

Polymer **P3-A**

Polymer **P3** (39.82 mg, 39.39 µmol) and dihexylamine (422.74 µL, 1.58 mmol) were stirred in 1,4-dioxane (5 mL) n a 1-dram vial at 100 °C for 5 days. Afterwards, the solvent was evaporated and the polymeric product was resuspended in acetone and precipitated with water. The precipitated solution was filtered, and washed with water, methanol, hexanes, diethyl ether, and acetone before being washed through with dichloromethane (51% recovery). ^{1}H NMR (500 MHz, $CDCl_3$) δ 6.44 – 5.73 (m), 4.82 (m), 3.92 – 2.55 (m), 1.55 (m), 1.40 – 0.96 (m), 0.95 – 0.69 (m).

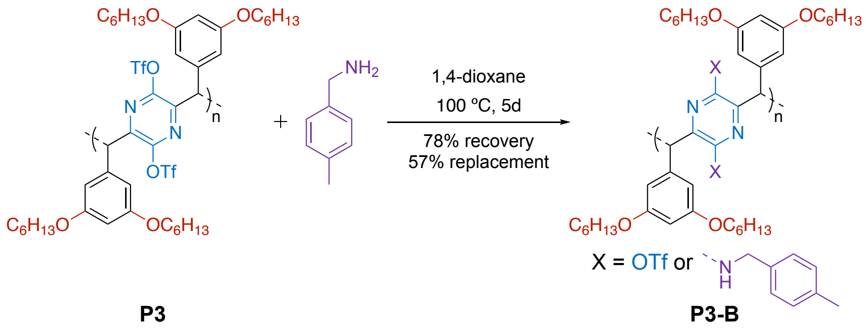

Polymer **P3-B**

Polymer **P3** (41.89 mg, 41.43 µmol) and 4-methylbenzylamine (210.97 µL, 1.66 mmol) were stirred in 1,4-dioxane (5 mL) in a 1-dram vial at 100 °C for 5 days. Afterwards, the solvent was

evaporated and the polymeric product was resuspended in acetone and precipitated with water. The precipitated solution was filtered, and washed with water, methanol, hexanes, diethyl ether, and acetone before being washed through with dichloromethane (78% recovery). ^{1}H NMR (500 MHz, $CDCl_3$) δ 7.02 (m), 6.41 – 5.88 (m), 4.70 (m), 3.51 (m), 2.45 – 2.13 (m), 1.59 (m), 1.29 (m), 1.02 – 0.74 (m).

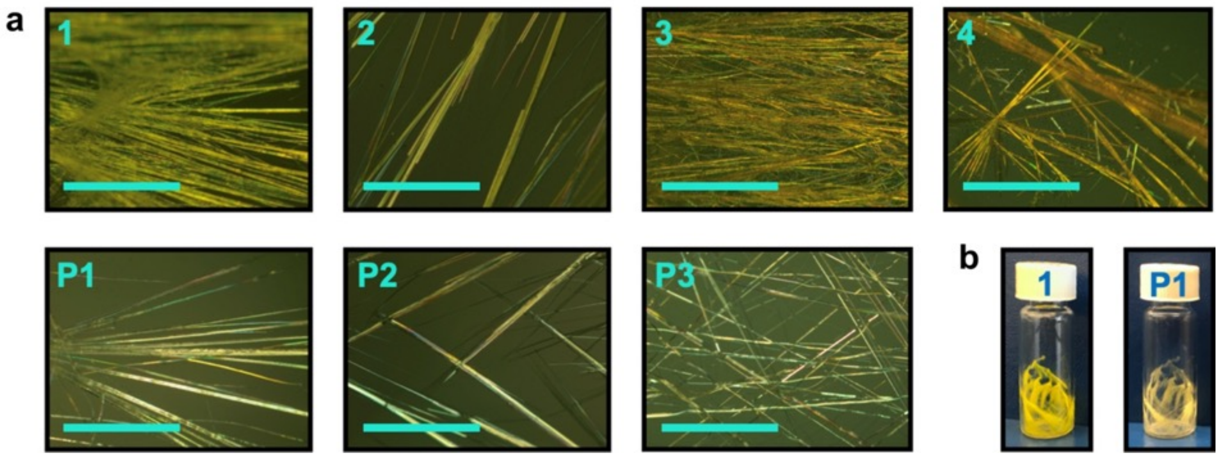

Supplementary Figure 3. (**a**) Optical microscope images under 90° polarization showing the typical morphology of crystals of monomers **1**-**4** (top row) and polymers **P1**-**P3** (bottom row). Scale bar: 1 mm. (**b**) Photographs of vials containing crystals of **1** and **P1**.

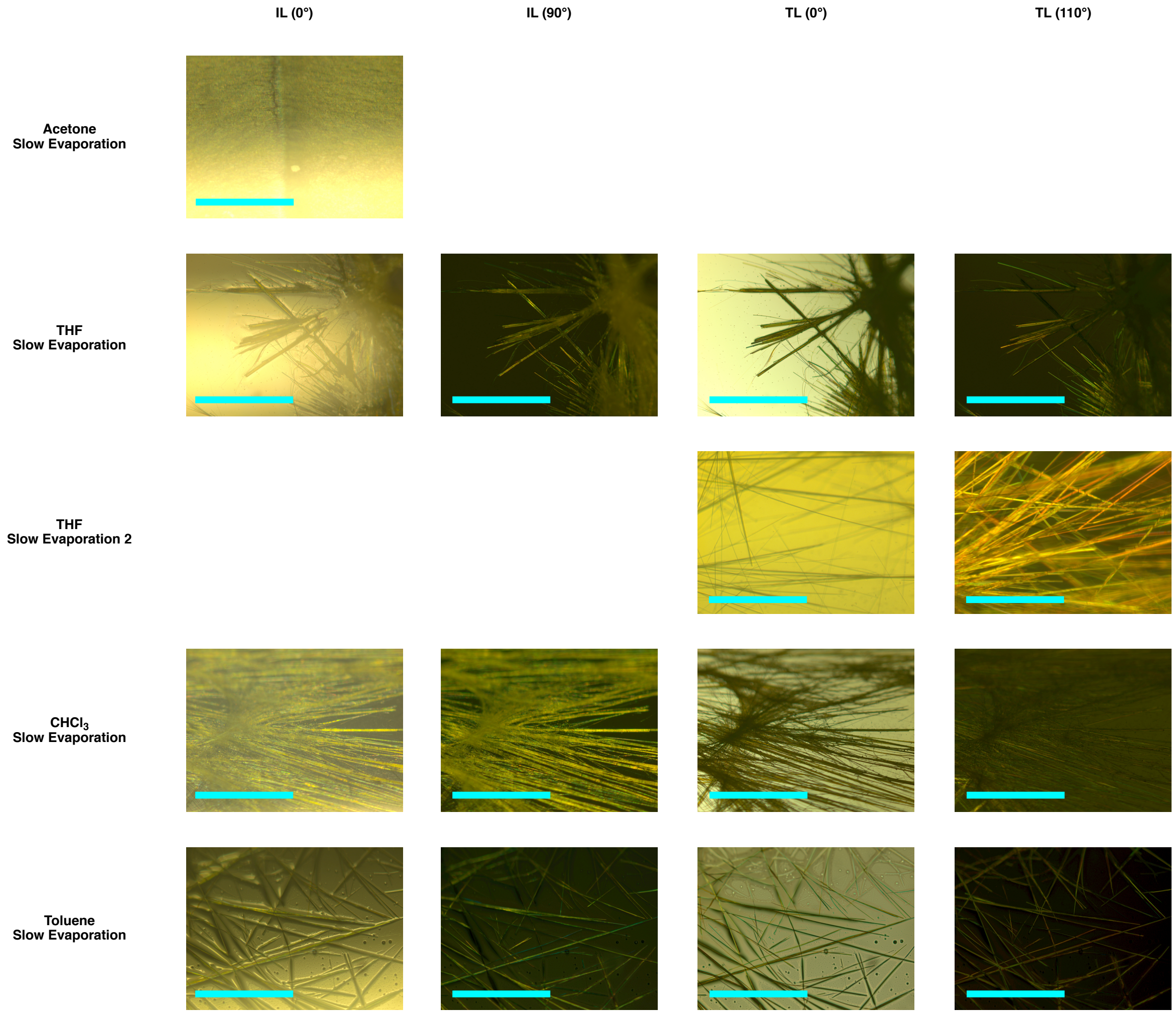

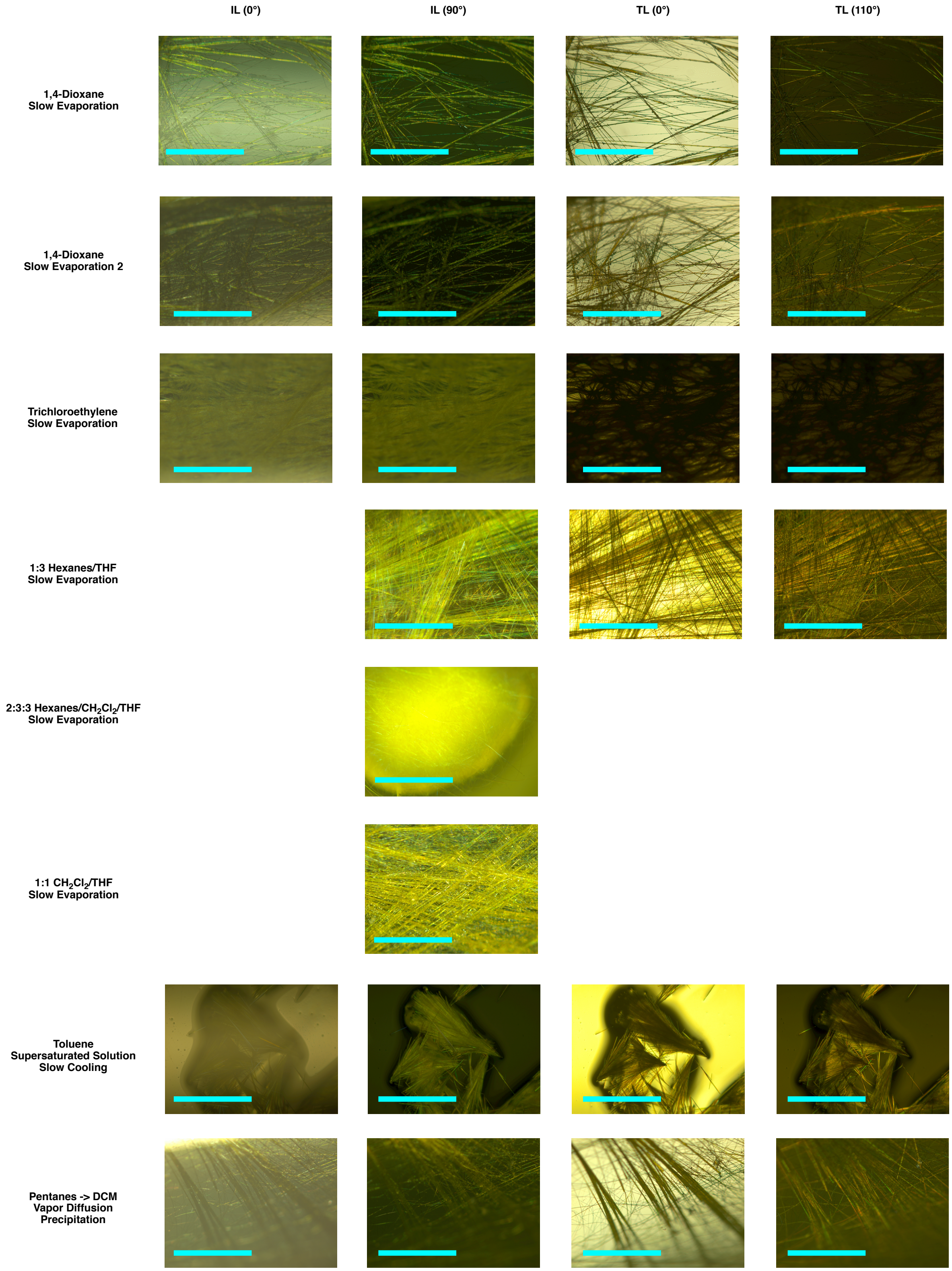

Supplementary Figure 4. Optical microscope images of monomeric crystals of **1** grown under different conditions (left) obtained with either transmitted light (TL) or incident light (IL) of the given polarization. Scale bar: 1 mm

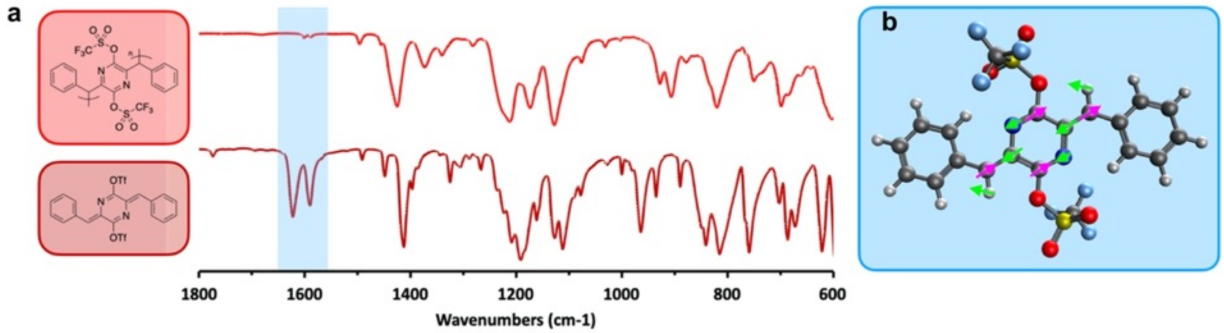

Supplementary Figure 5: Fourier-Transform Infrared Spectroscopy (FTIR) characterization of monomer **1** and polymer **P1**. (**a**) The fingerprint regions of the FTIR traces of **1** and **P1** highlighting the disappearance of the quinoidal ring exocyclic methylene feature upon polymerization. (**b**) Computed vibrations of the quinoidal ring exocyclic methylene corresponding to the feature at 1570–1635 cm^{-1} in **1** (B3LYP/6-311++G**).

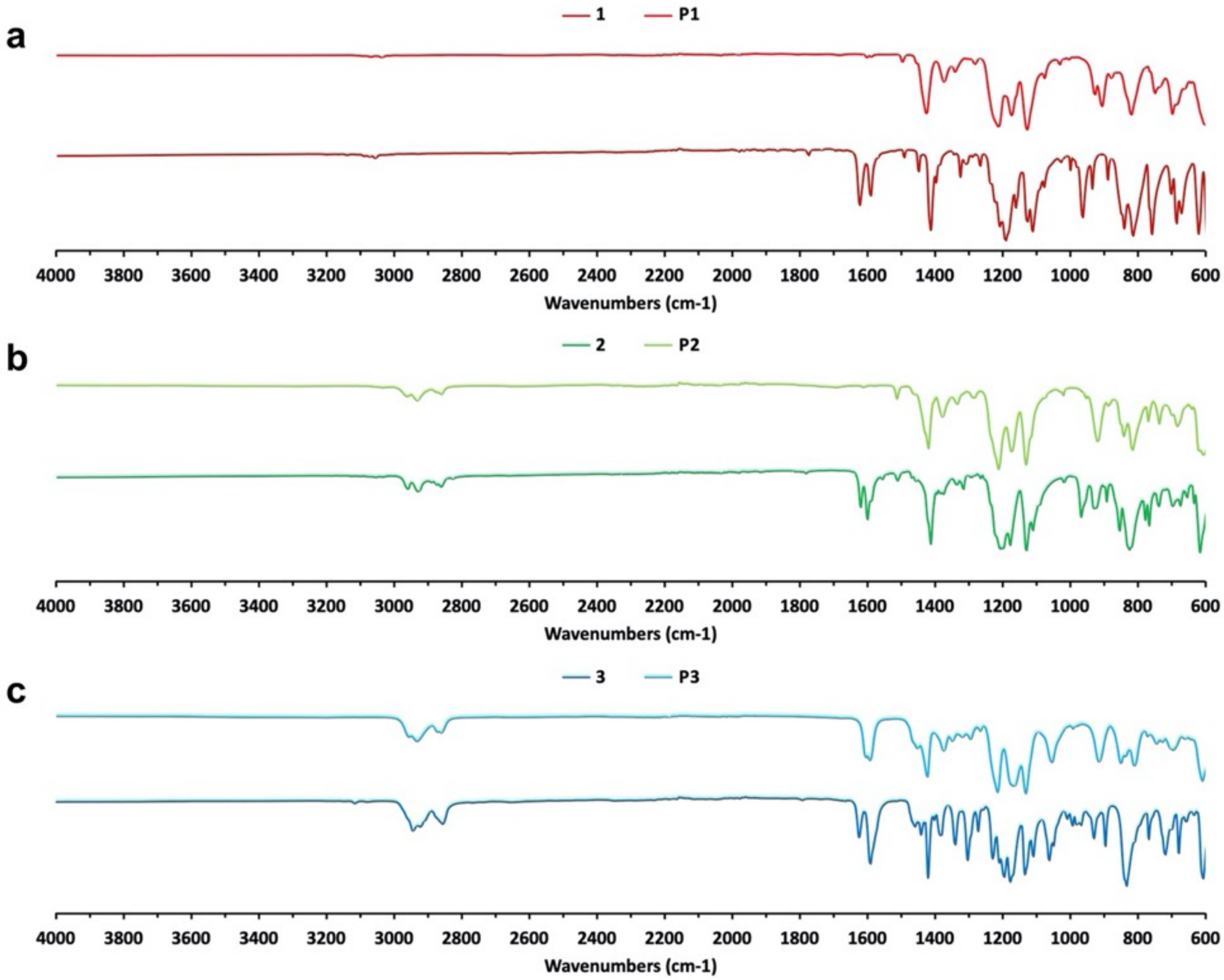

Supplementary Figure 6. FTIR spectra of monomers **1**-**3** and polymers **P1**-**P3**. (**a**) FTIR of **1** vs. **P1**. (**b**) FTIR of **2** vs. **P2**. (**c**) FTIR of **3** vs. **P3**. The peak at 1600 cm^{-1} in **P3** corresponds to the phenolic stretching vibration which overlaps with the exocyclic methylene vibration.

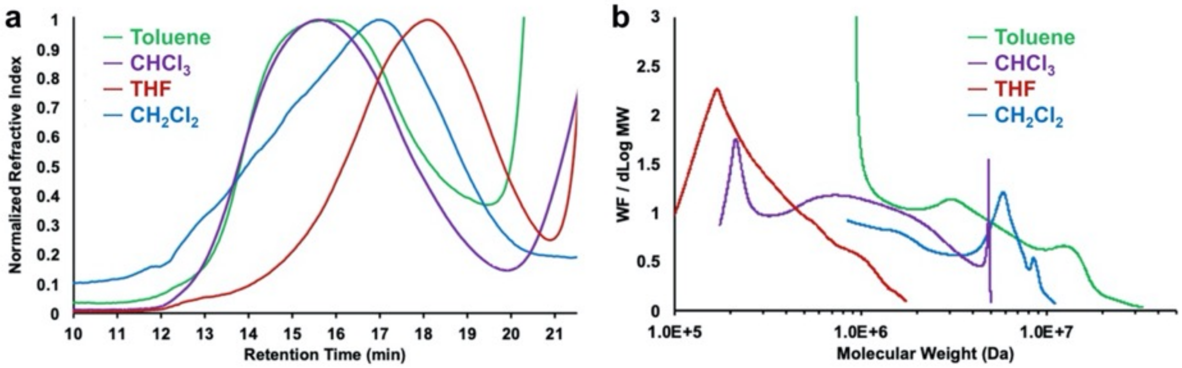

c

Solvent	Mn (10^5)	Mw (10^5)	PDI
Toluene	19.44	38.74	1.99
$CHCl_3$	5.43	11.72	2.16
THF	2.15	3.42	1.59
CH_2Cl_2	7.96	20.78	2.61

Supplementary Figure 7. Molecular weight distributions of **P3** generated via crystallization from different solvents. (**a**) Size-exclusion chromatography (SEC) traces. (**b**) SEC-derived molecular weight distributions displayed on a logarithmic scale. (**c**) Molecular weight data derived from the SEC traces shown in **a**.

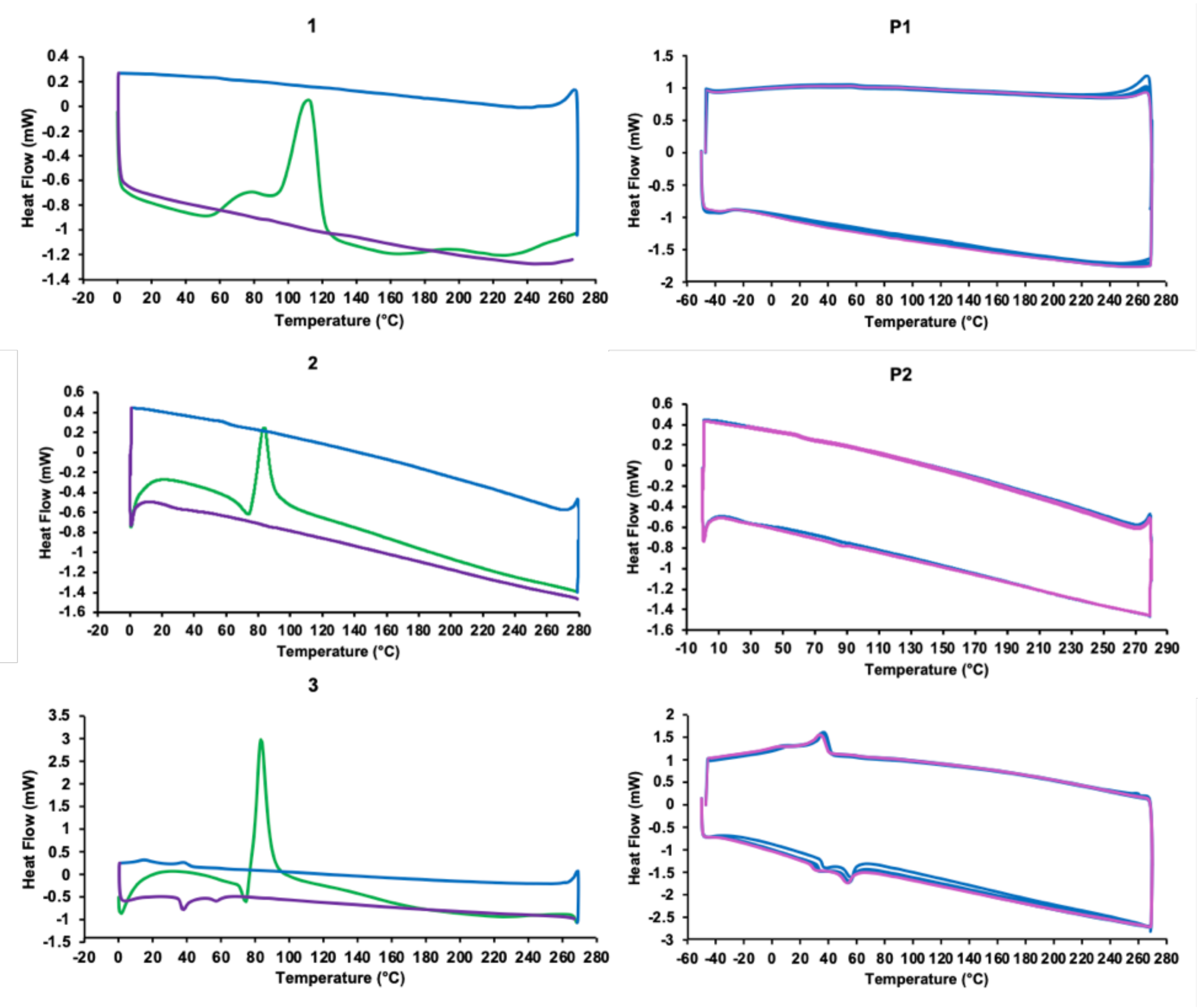

Supplementary Figure 8. Differential Scanning Calorimetry (DSC) traces. The left column shows the first heating (green), first cooling (blue), and second heating (purple) scans of monomers **1**-**3**. The right column shows multiple heating and cooling cycles (blue = first four cycles, pink = final cycle) of **P1**-**P3**.

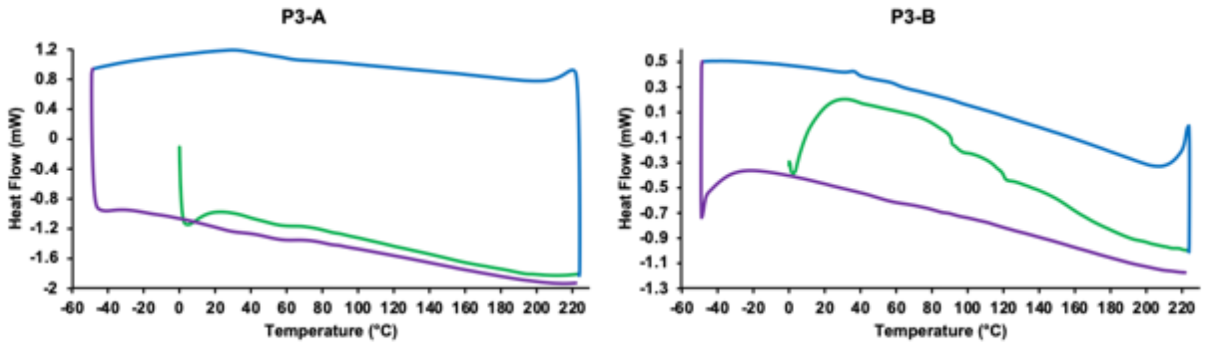

Supplementary Figure 9. Differential Scanning Calorimetry (DSC) traces of post-polymerization functionalization products **P3-A** and **P3-B** showing the first heating (green), first cooling (blue), and second heating (purple) scans.

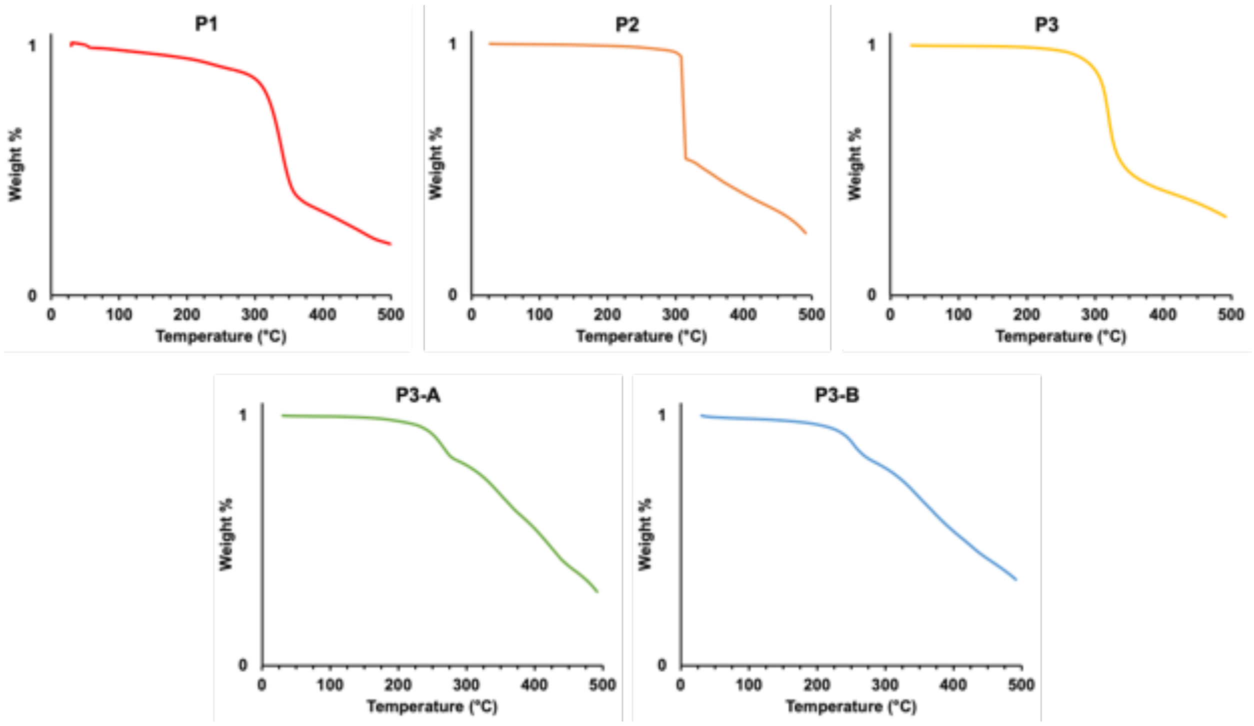

Supplementary Figure 10. Thermogravimetric analysis (TGA) traces of polymers **P1**-**P3** and post-polymerization functionalized **P3-A** and **P3-B**. Note: separate TGA traces were not taken for **1**-**3** as they were expected to have polymerized during the course of temperature ramping.

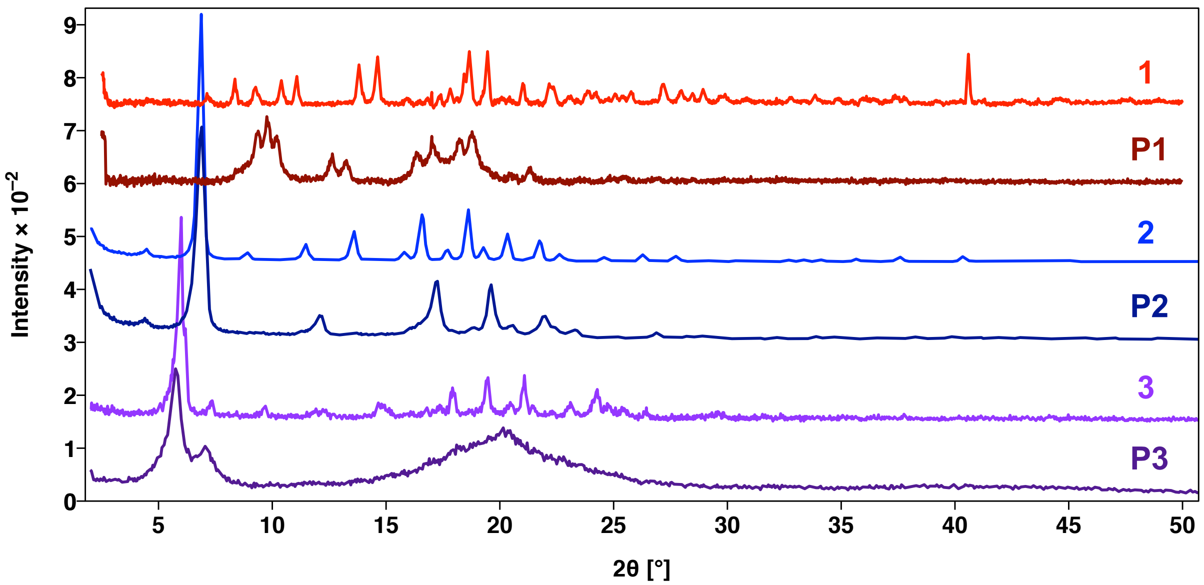

Supplementary Figure 11. Powder X-ray diffraction (PXRD) traces of monomers **1**-**3** and polymers **P1**-**P3**. Intensity axis is scaled for each trace so as to allow for comparison of the data.

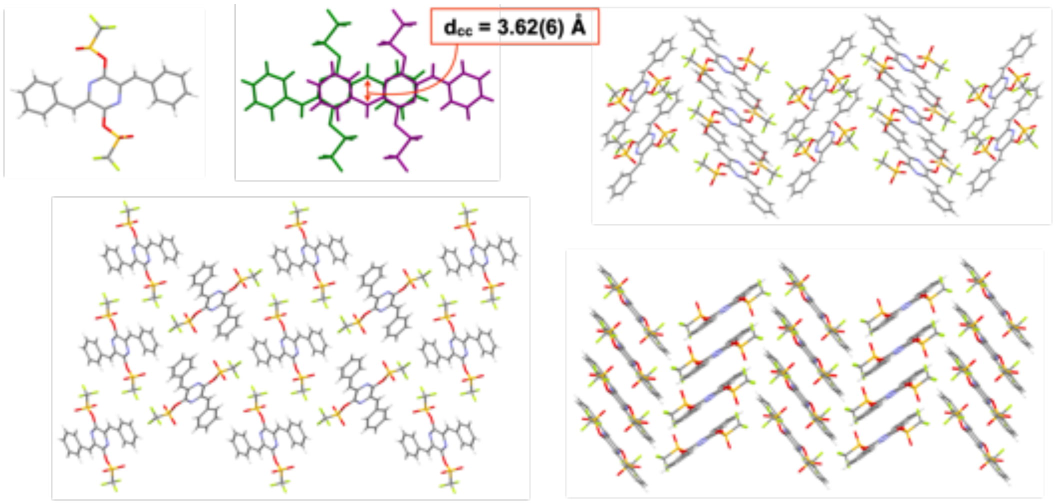

Supplementary Figure 12. Stick representations of X-ray crystallographic structure of monomer **1**, showing molecular structure (top left), d_{CC} distance (top middle), and three different views of molecular packing.

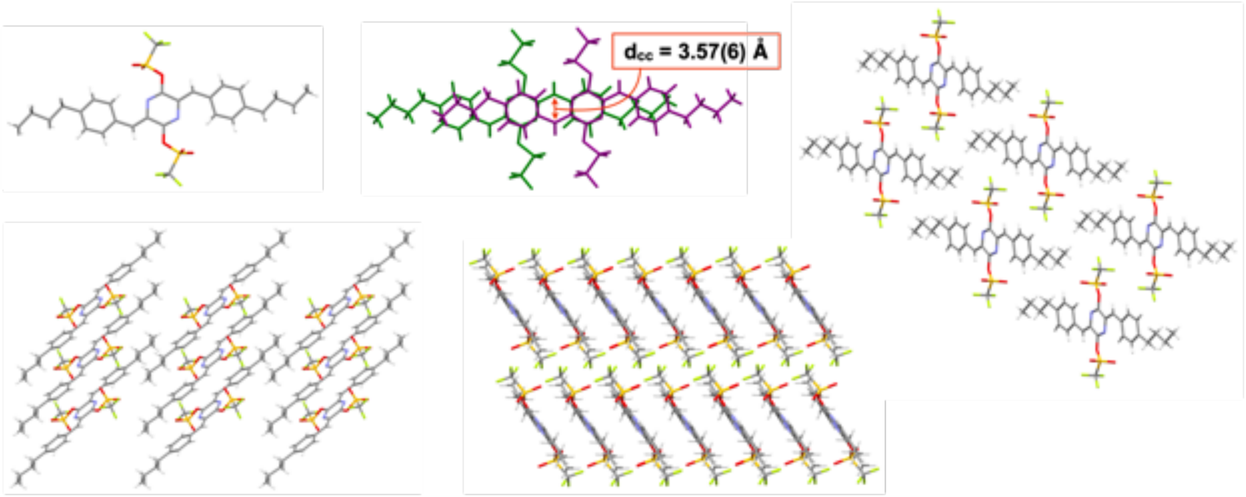

Supplementary Figure 13. Stick representations of X-ray crystallographic structure of monomer **2**, showing molecular structure (top left), d_{CC} distance (top middle), and three different views of molecular packing.

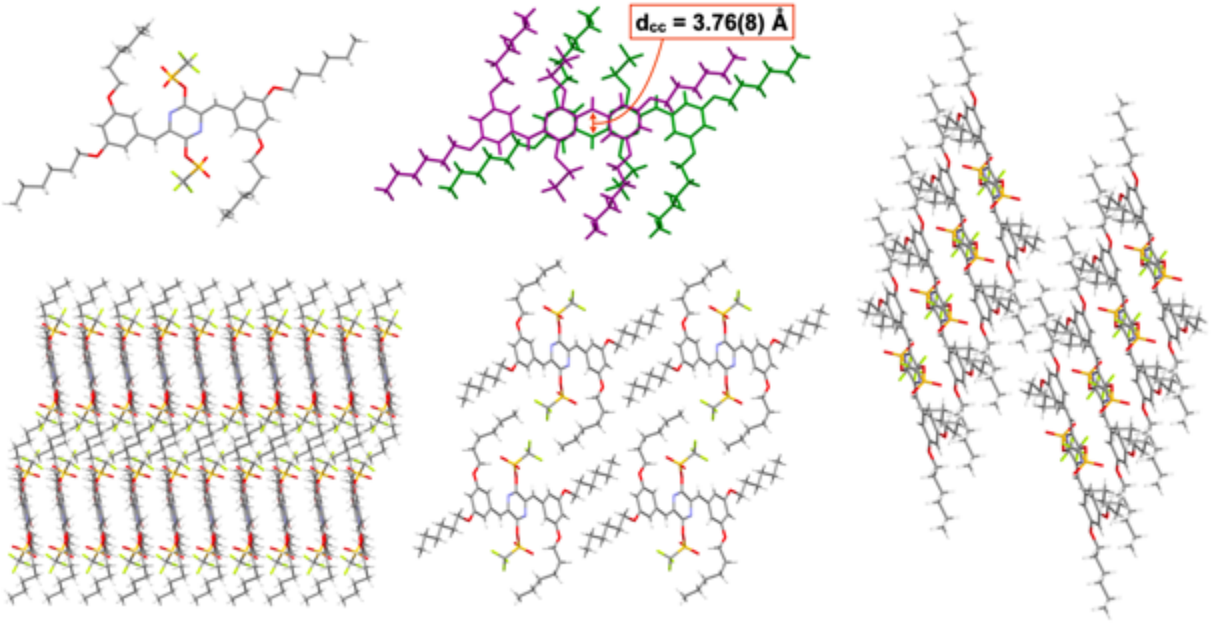

Supplementary Figure 14. Stick representations of X-ray crystallographic structure of monomer **3**, showing molecular structure (top left), d_{CC} distance (top middle), and three different views of molecular packing.

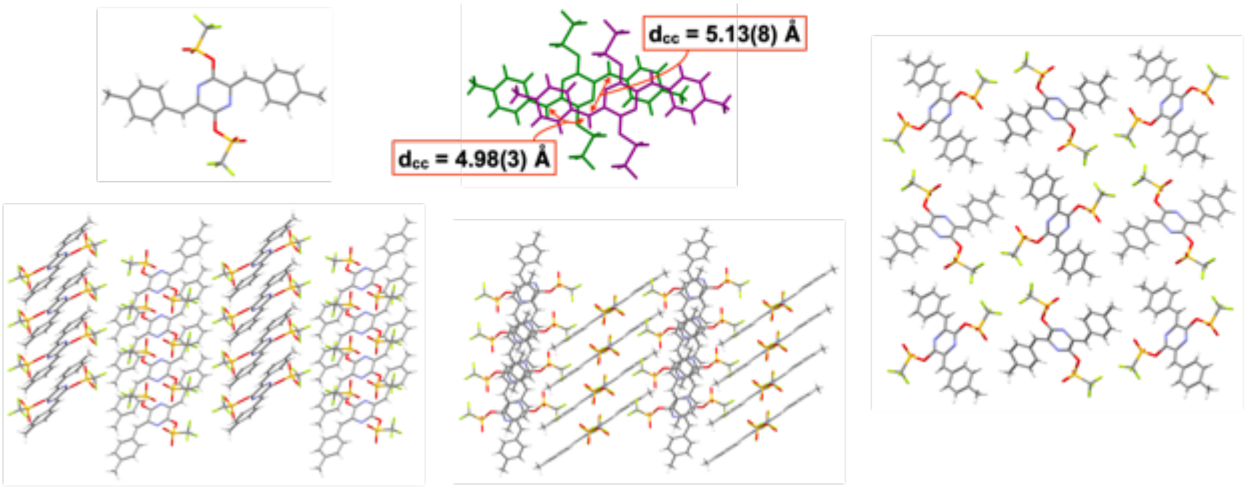

Supplementary Figure 15. Stick representations of X-ray crystallographic structure of monomer **4**, showing molecular structure (top left), two possible d_{CC} distances (top middle), and three different views of molecular packing.

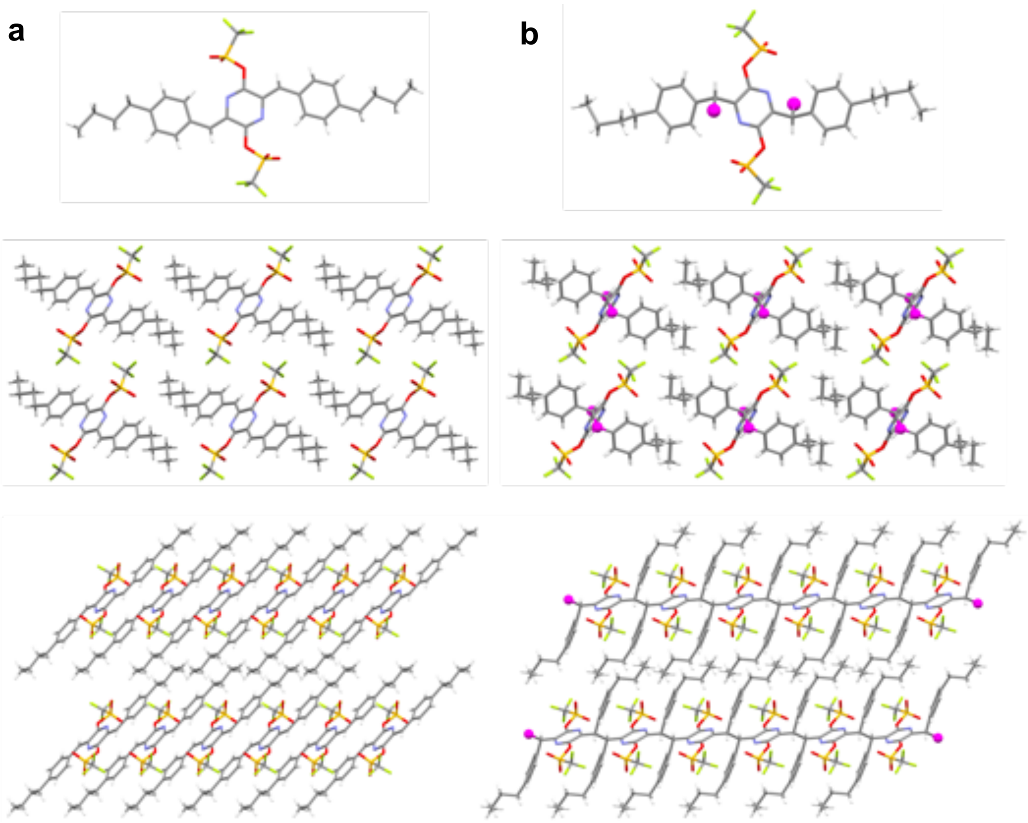

Supplementary Figure 16. Stick representations of crystallographic structure of (**a**) monomer **2** (via X-ray diffraction), and (**b**) polymer **P2** (via cryoEM) showing molecular structure (left) and different views of molecular packing (right).

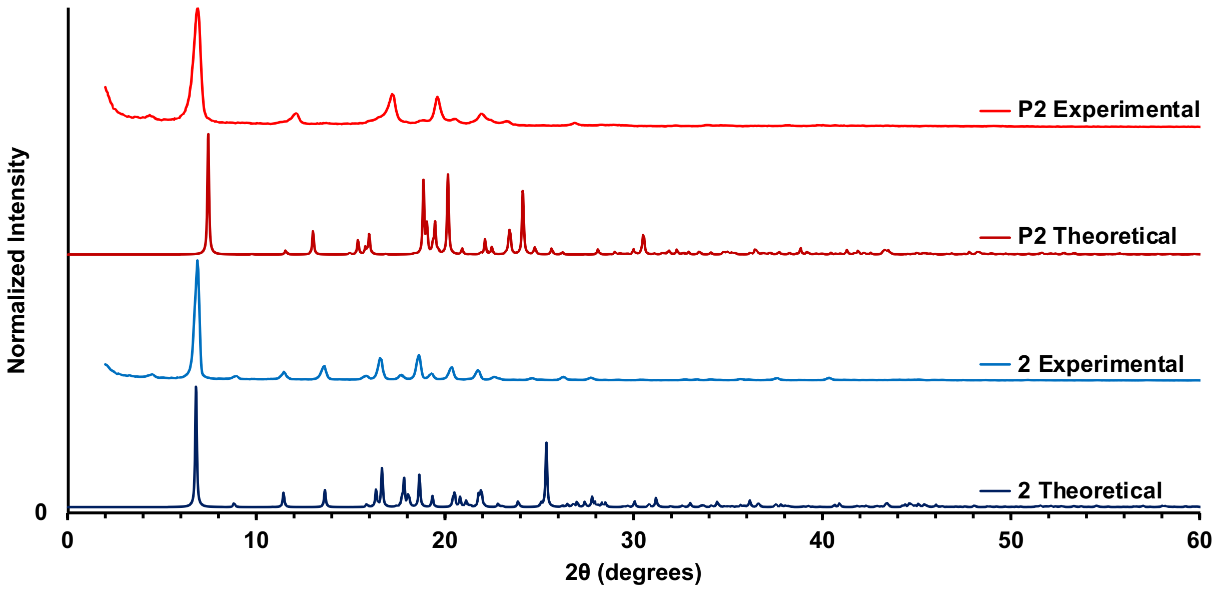

Supplementary Figure 17. Experimental PXRD traces (Experimental) and PXRD traces predicted based on the obtained crystal structure of monomer **2** and cryoEM structure of polymer **P2** (Theoretical). Intensity axis is scaled for each trace so as to allow for comparison of the data. Note: The peak shifts between P2 experimental and P2 theoretical are ascribed to a temperature effect, since P2 experimental was acquired at 25 ºC while the theoretical trace was based on data acquired at –196 ºC. This temperature effect was supported by variable temperature PXRD results shown in Supplementary Figures 18 and 19.

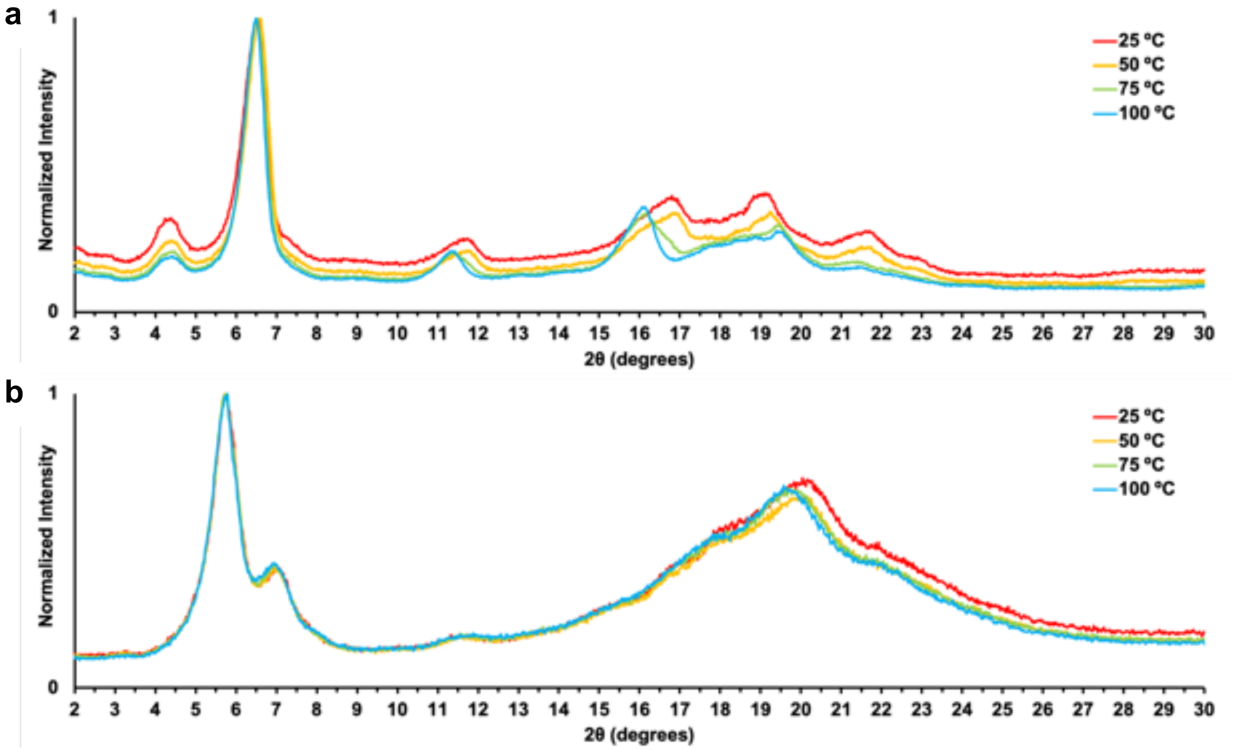

Supplementary Figure 18. Effects of temperature on PXRD traces of polymers (**a**) **P2** and (**b**) **P3** showing the changes in the normalized PXRD traces of each at 25, 50, 75, and 100 ºC.

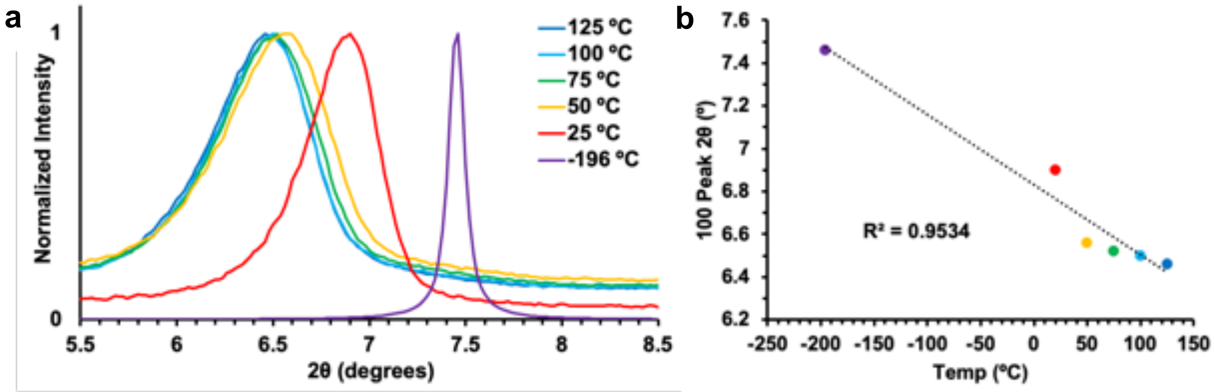

Supplementary Figure 19. Effects of temperature on PXRD traces of polymer **P2**. (**a**) The 100 peak in PXRD traces taken at various different temperatures (traces at 25–125 ºC are experimental, the -196 ºC trace is predicted from the cryoEM structure). (**b**) The correlation between temperature and the 100 peak maximum value, showing a consistent increase of d spacing at higher temperature. A linear line is shown to indicate the trend.

Supplementary Table 1. Numerical summary of X-ray crystallographic and cryoEM results for monomers **1-4** and polymer **P2**

**Compound**	**Space Group**	**Cell Lengths (Å)**	**Cell Angles (°)**	**Cell Volume (Å)**	**d_{CC} (Å)**	**R factor (%)**
**1**	P-1	a: 5.5195(3)<br>b: 10.6780(5)<br>c: 19.1750(9)	α: 101.861(2)<br>β: 94.954(2)<br>γ: 95.245(2)	1094.93(9)	3.62(6)	4.30
**2**	P-1	a: 5.5851(5)<br>b: 10.319(1)<br>c: 13.0045(13)	α: 92.459(4)<br>β: 92.246(4)<br>γ: 103.837(4)	726.12(12)	3.57(6)	6.25
**P2**	P-1	a: 6.080(5)<br>b: 9.450(4)<br>c: 12.390(12)	α: 92.35(7)<br>β: 105.49(16)<br>γ: 105.58(11)	655.9(11)	1.50(2)	18.62
**3**	P-1	a: 5.4708(7)<br>b: 14.4268(18)<br>c: 15.2986(19)	α: 73.767(5)<br>β: 85.910(5)<br>γ: 87.203(5)	1155.8(3)	3.76(8)	6.36
**4**	P 42/n	a: 22.0352(13)<br>b: 22.0352(13)<br>c: 4.8929(4)	α: 90<br>β: 90<br>γ: 90	2375.8(3)	4.89(3)[a]<br>5.13(8)[a]	3.85

[a]Different d_{CC} values can be obtained depending on which methylene carbons are assumed to react with each other

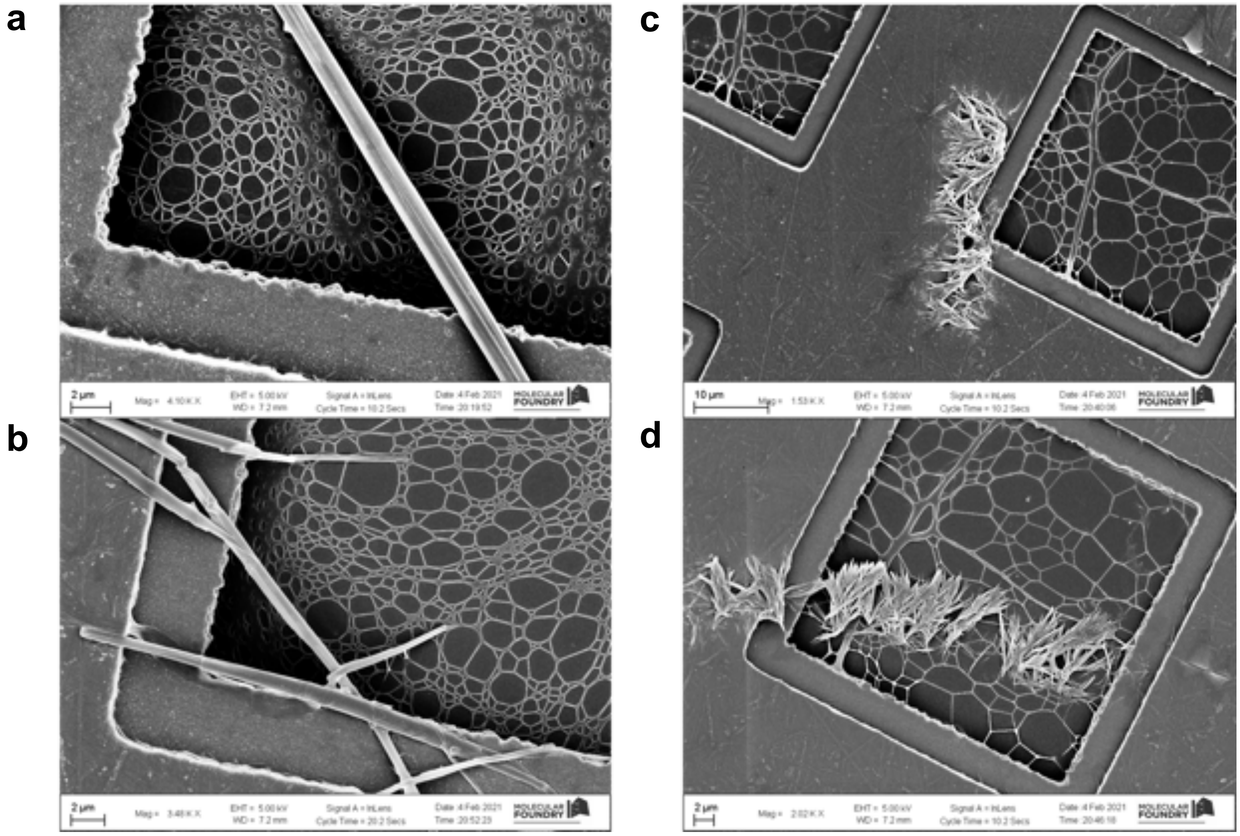

Supplementary Figure 20. Scanning electron microscopy (SEM) images of crystals of **P2**, generated from crystals of monomer **2** deposited on TEM grids via dip-coating from (**a**, **b**) chloroform, and (**c**, **d**) dichloromethane and polymerized on the grids. See Supplementary Figure 1 for microcrystal deposition process.

CH_2Cl_2 **THF** **$CHCl_3$**

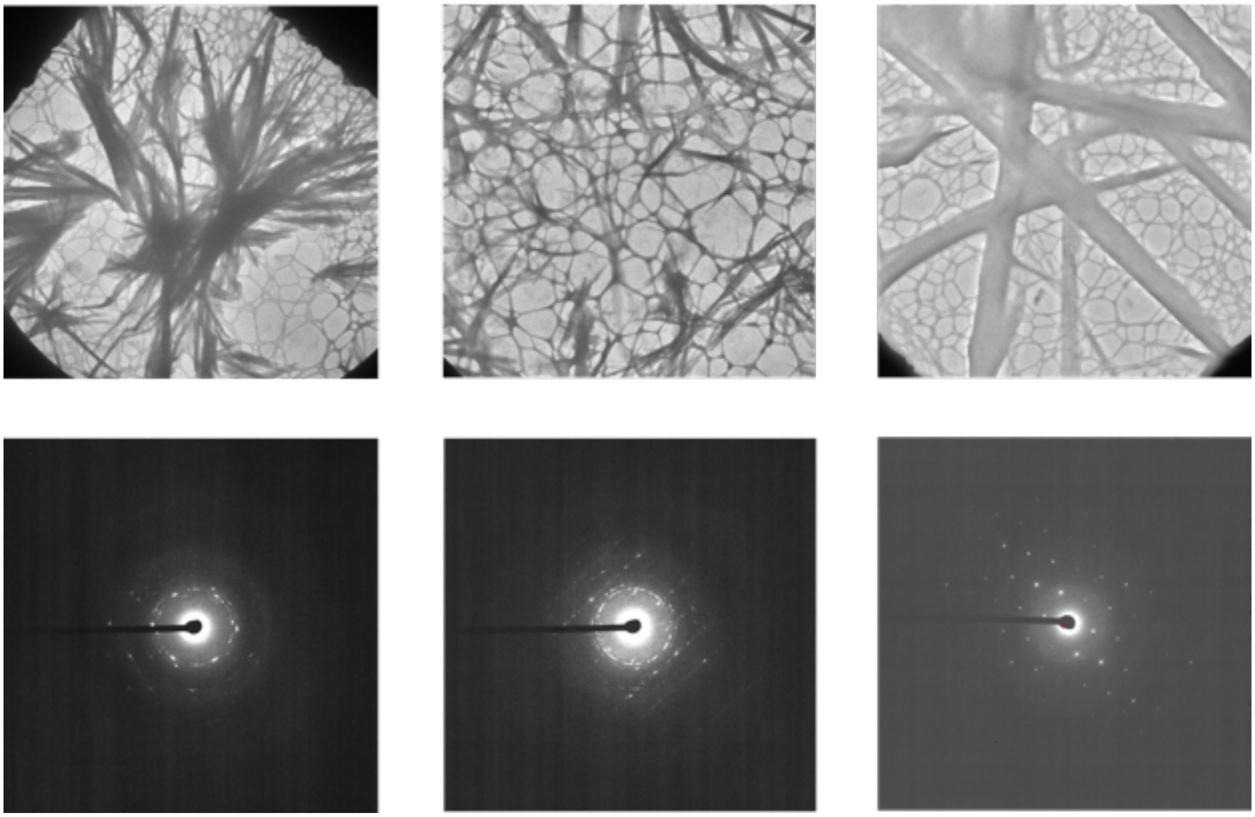

Supplementary Figure 21. Transmission electron microscopy (TEM) images of crystals of **P2** (top) and representative electron diffraction patterns from each (bottom). **P2** crystals were generated from crystals of monomer **2** deposited on TEM grids via dip-coating from dichloromethane (CH_2Cl_2), tetrahydrofuran (THF), and chloroform ($CHCl_3$). See Supplementary Figure 1 for microcrystal deposition process.

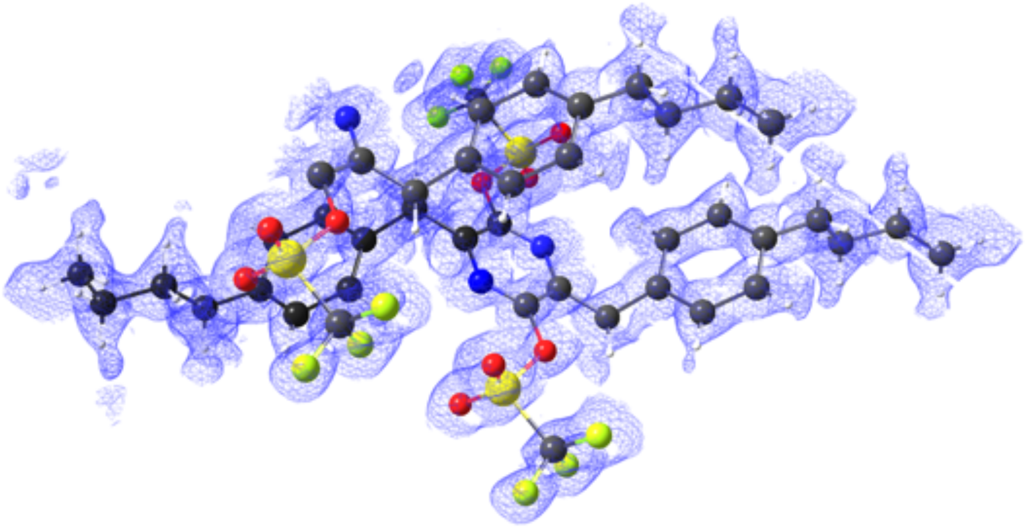

Supplementary Figure 22. CryoEM-derived 3D electron density map with the unit cell of the polymeric **P2** structure obtained therefrom shown within it.

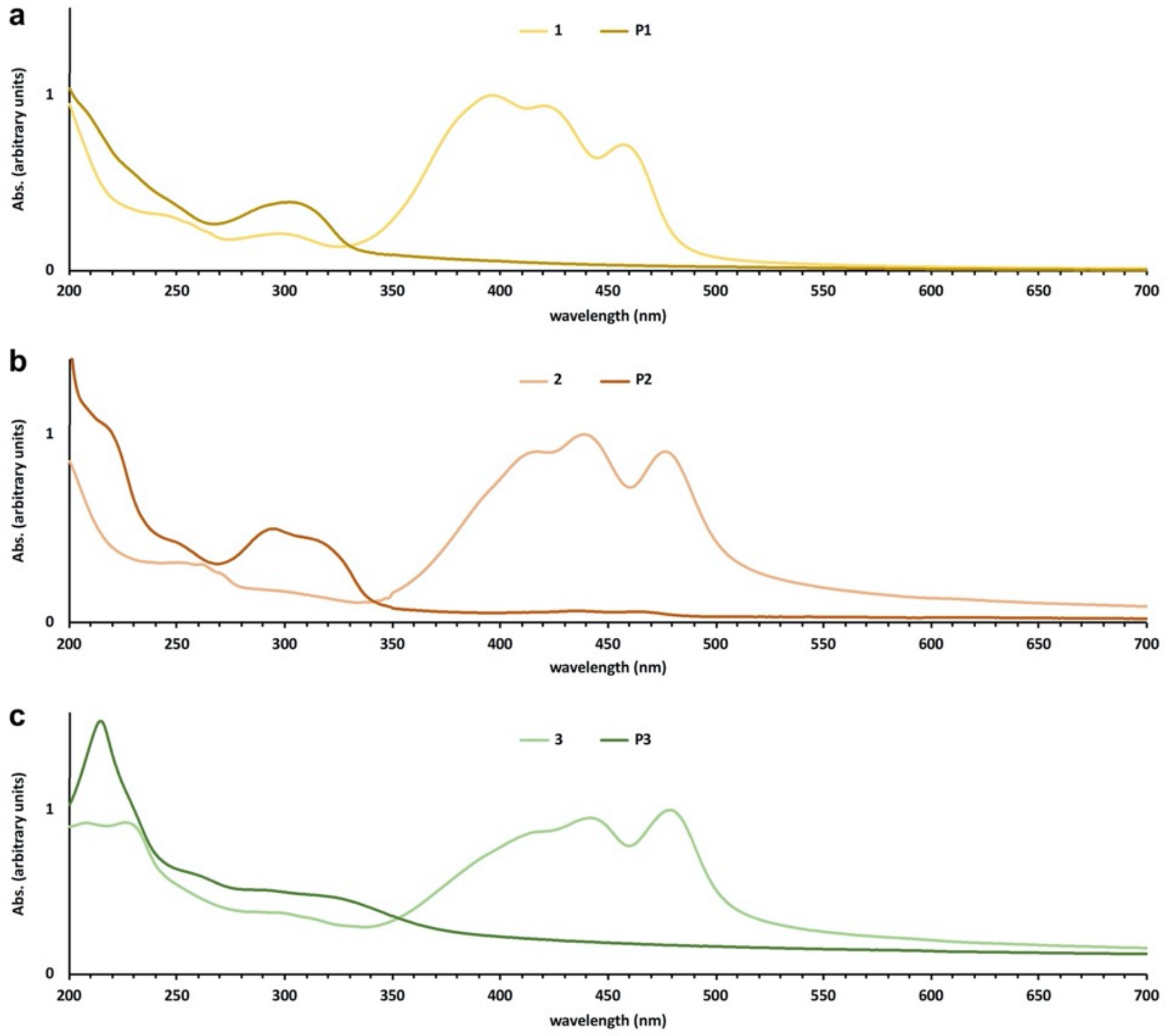

Supplementary Figure 23. UV-Visible absorption spectra of films of (**a**) **1** and **P2**, (**b**) **2** and **P2**, and (**c**) **3** and **P3** on quartz substrates.

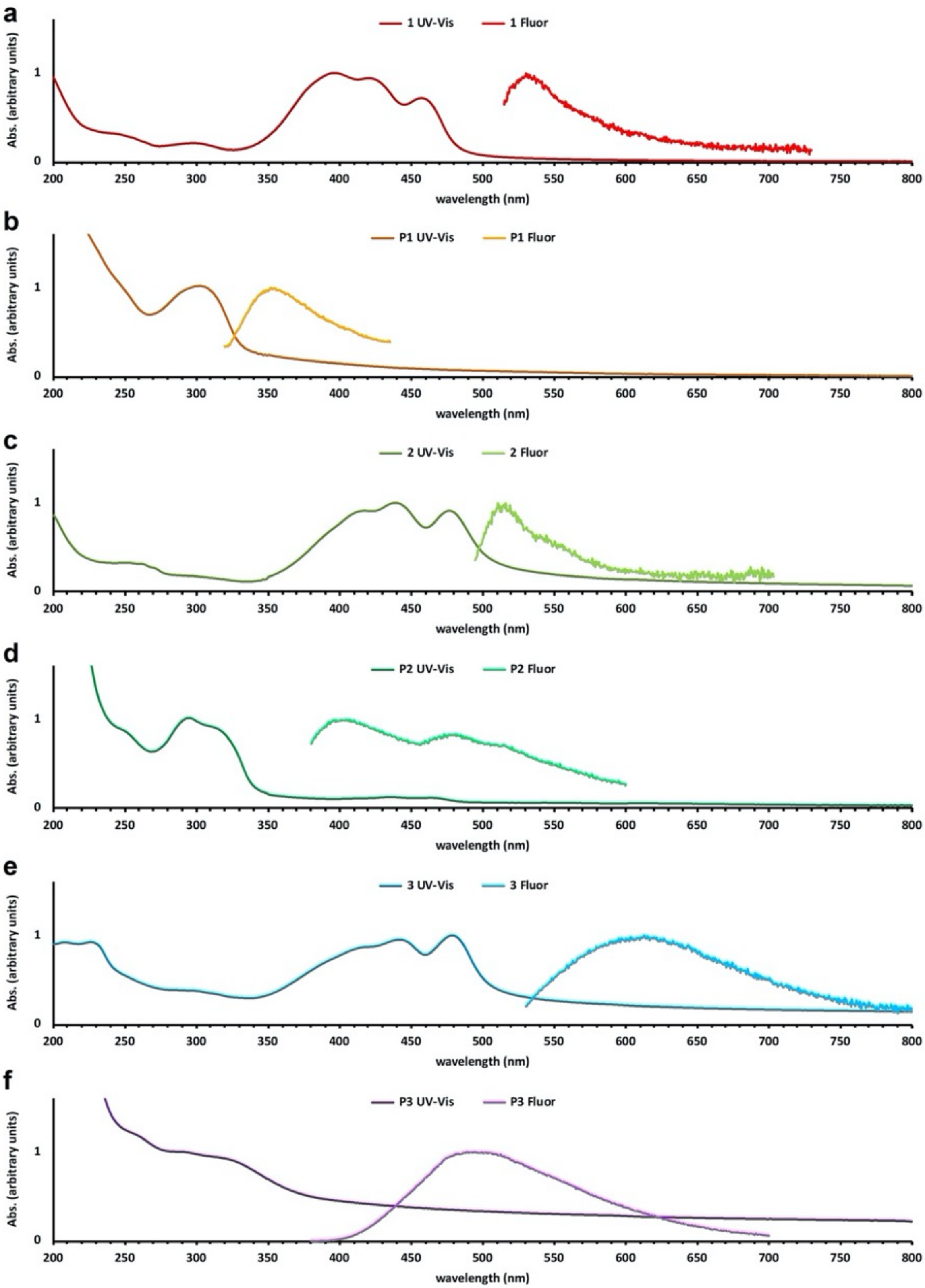

Supplementary Figure 24. UV-Visible absorption and fluorescence spectra of solutions of (**a**) **1**, (**c**) **2**, (**e**) **3** (solvent: CH_2Cl_2) and films of (**b**) **P1**, (**d**) **P2**, and (**f**) **P3**.

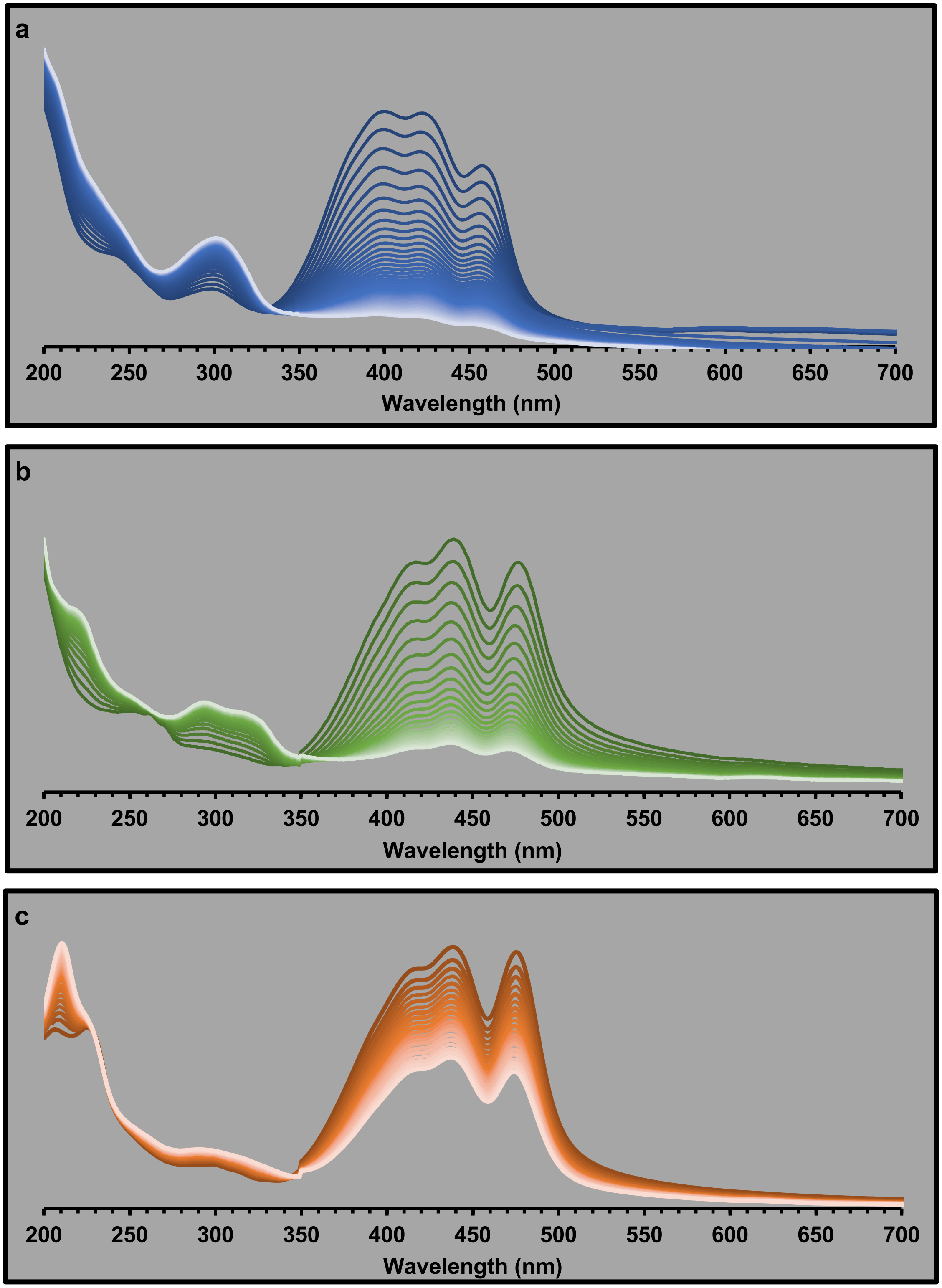

Supplementary Figure 25. UV-Vis scanning kinetics experiment run on films of (**a**) **1**, (**b**) **2**, and (**c**) **3** on quartz substrates showing the (partial) conversion from monomer (blue, green, or orange) to polymer (white) over roughly 8 hours at room temperature and protected from ambient light.

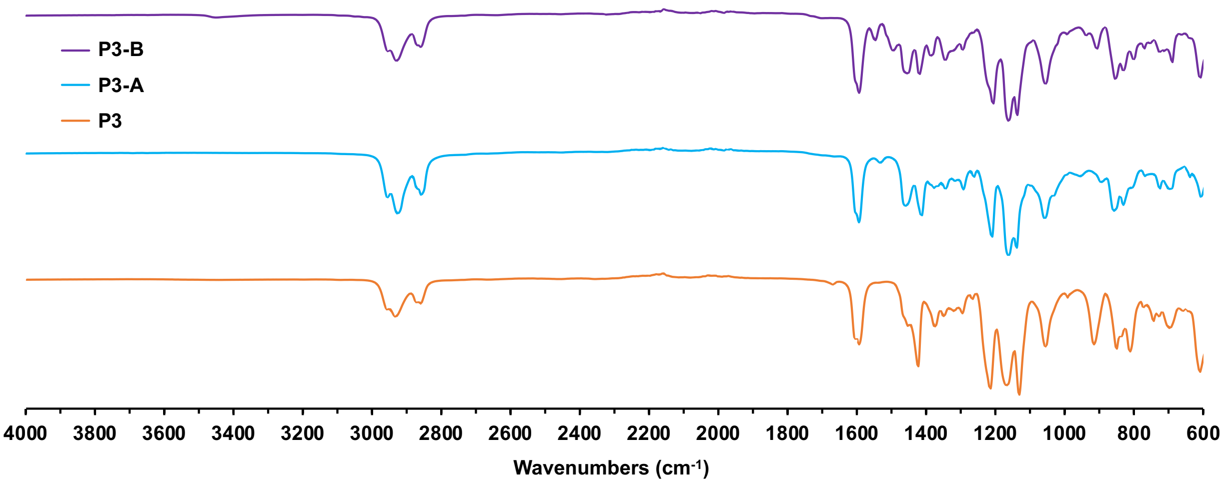

Supplementary Figure 26. FTIR spectra of **P3**, **P3-A**, and **P3-B**.

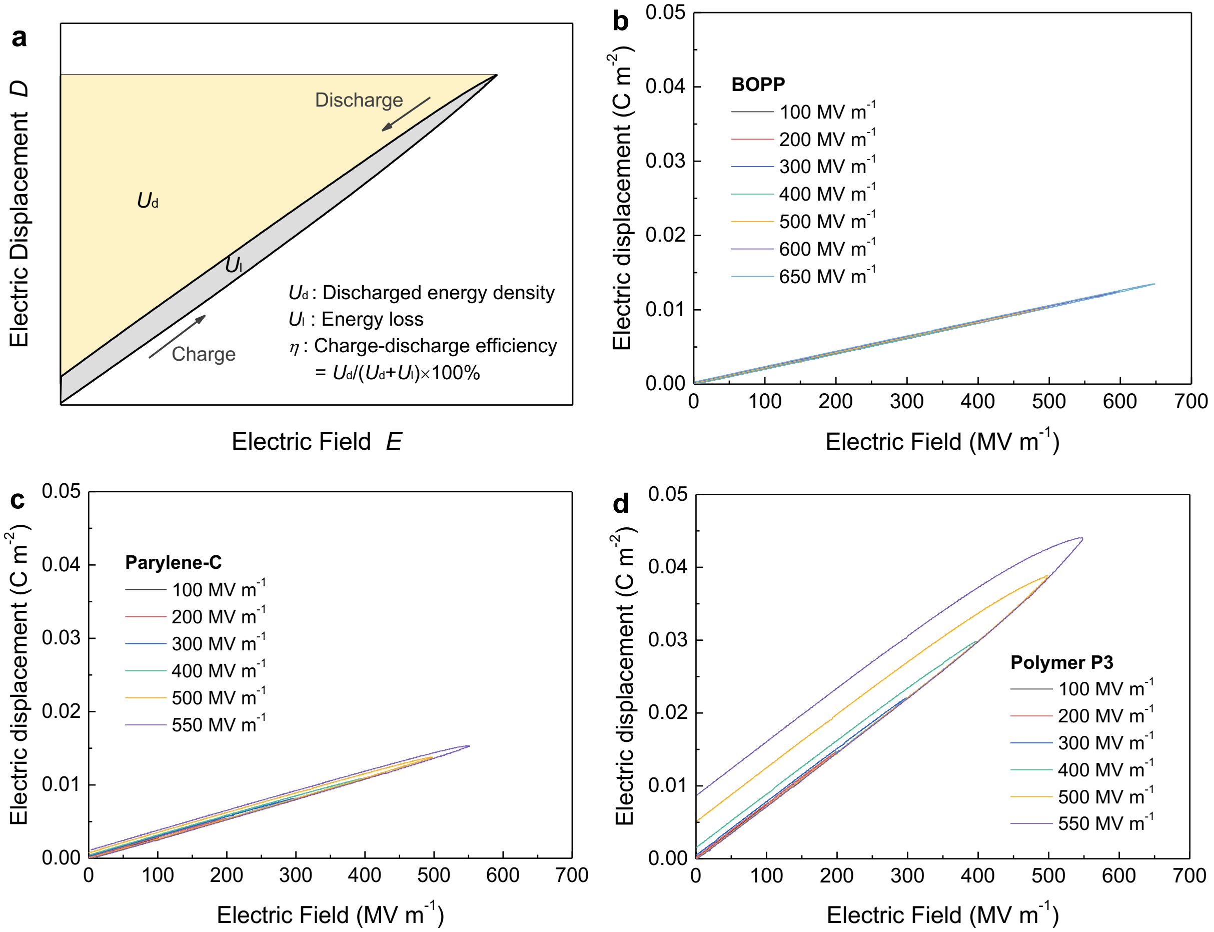

Supplementary Figure 27. Electric displacement–electric field (*D*–*E*) loops of dielectric polymers described herein (**a**) Schematic *D*–*E* loop of a linear dielectric material with the discharged energy density (U_d) represented by the yellow region and energy loss (U_l) indicated by the gray region. The total charged energy density equals U_e plus U_l, and the charge–discharge efficiency (η) can be calculated by $\eta = U_d/(U_d+U_l) \times 100\%$.[14] Unipolar *D*–*E* loops of (**b**) BOPP, (**c**) parylene-C, and (**d**) polymer **P3**. The effective *K* values were determined from the slope in the linear range of the *D*–*E* loops (*i.e.*, at 200 MV m^{-1}), which amounted to 2.29, 2.87 and 8.14 for BOPP, parylene-C and polymer **P3**, respectively.[15]

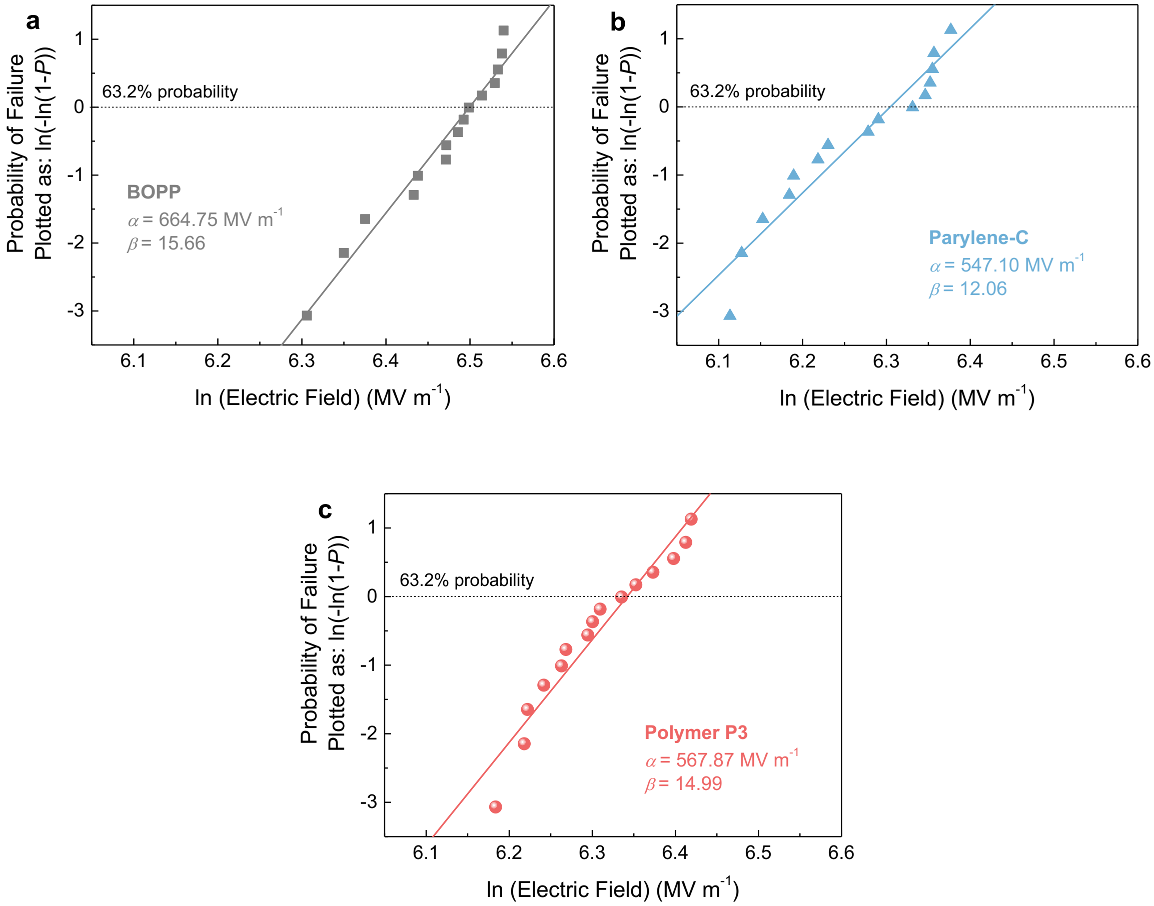

Supplementary Figure 28. Weibull plots of dielectric breakdown strength for (**a**) BOPP, (**b**) parylene-C, and (**c**) polymer **P3**. The fitting model is based on a two-parameter Weibull statistic that can be described as

$$P(E) = 1 - \exp(-(E/\alpha)^{\beta}) \qquad \textbf{(S1)}$$

where $P(E)$ is the cumulative probability of dielectric failure, E is the measured dielectric breakdown field, the scale parameter α is the characteristic breakdown strength which corresponds to a failure probability of 63.2%, the shape parameter β is associated with the scatter of data. A higher β value refers to a narrower data spread. The β values of BOPP, parylene-C, and polymer **P3** range from 12 to 16, signifying the relatively high dielectric reliability of these polymers. At least 15 measurements were performed for each Weibull fitting.

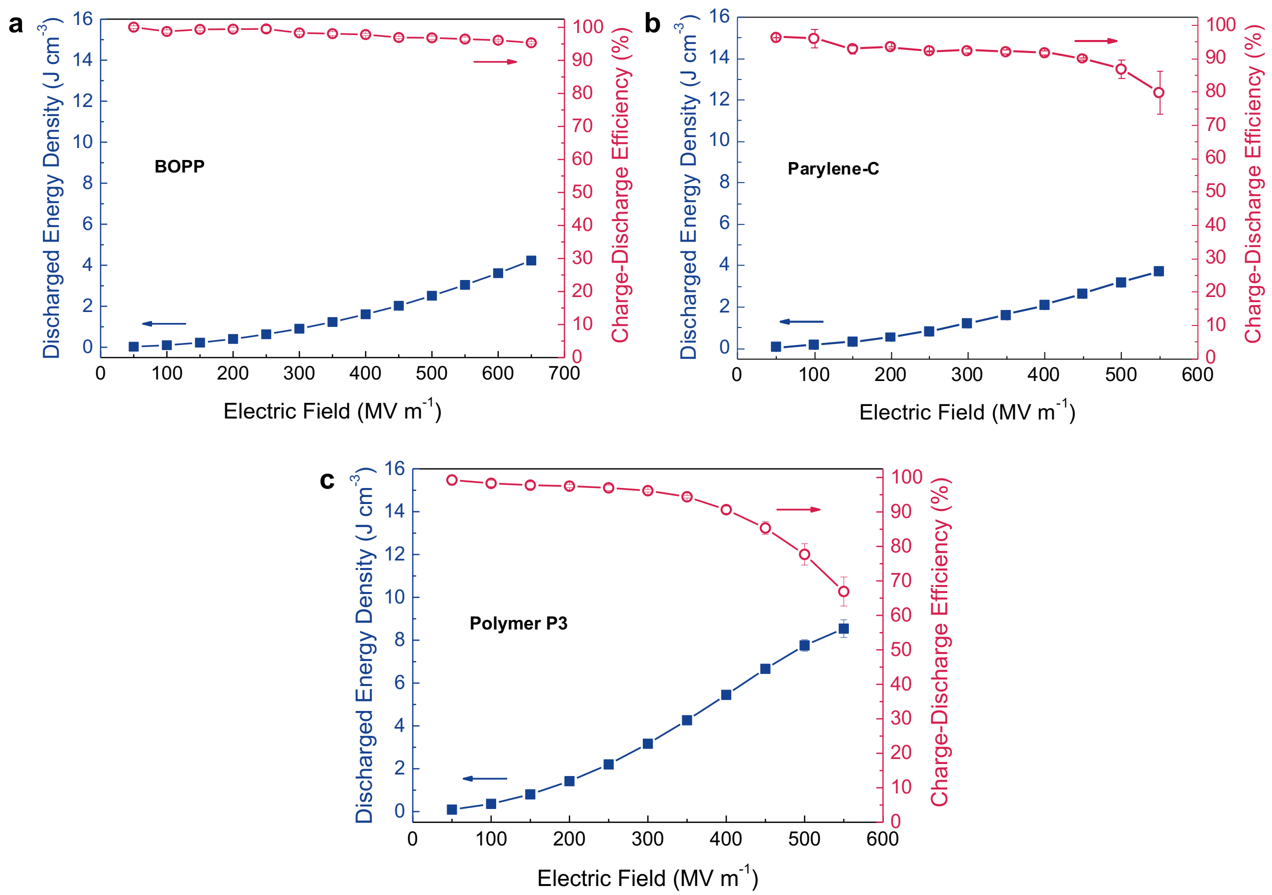

Supplementary Figure 29. Discharged energy density and charge–discharge efficiency plots as a function of electric field for (**a**) BOPP, (**b**) parylene-C, and (**c**) polymer **P3**.

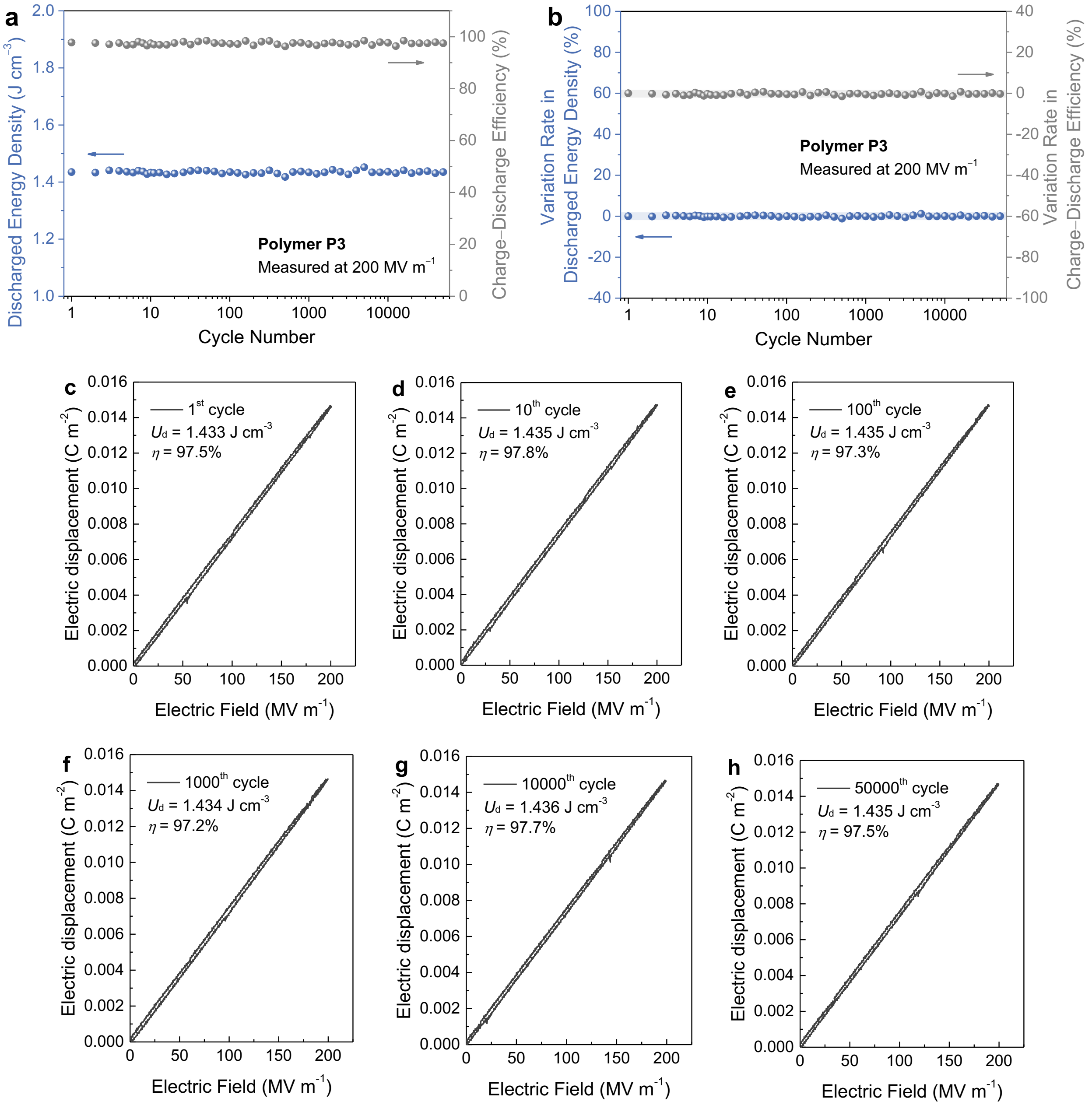

Supplementary Figure 30. Cyclability studies on polymer **P3**-based thin-film capacitors. (**a**) Discharged energy density and charge–discharge efficiency. (**b**) Variation rate in discharged energy density and charge–discharge efficiency of polymer **P3** as a function of cycle numbers obtained from 50,000 consecutive charge–discharge cycles. (**c**-**h**) *D*–*E* loops of polymer **P3** at 200 MV m^{-1} for the 1st, 10th, 100th, 1,000th, 10,000th, and 50,000th cycles, respectively.

The variation rate (R_v) of discharged energy density or charge–discharge efficiency is described as

$$R_v = |(U_{di} - U_{d1}) / U_{d1}| \quad \textbf{(S2)}$$

or

$$R_v = |(\eta_i - \eta_1) / \eta_1| \quad \textbf{(S3)}$$

where U_{d1} is the discharged energy density and η_1 is the charge–discharge efficiency of the 1st cycle, U_{di} is the discharged energy density and η_i is the charge–discharge efficiency of the ith cycle, *i* refers to the number of charge-discharge cycles. The variation rate in discharged energy density is < 1.2% while that in charge–discharge efficiency is < 1.6%, corresponding to outstanding device cyclability under a high electric field.

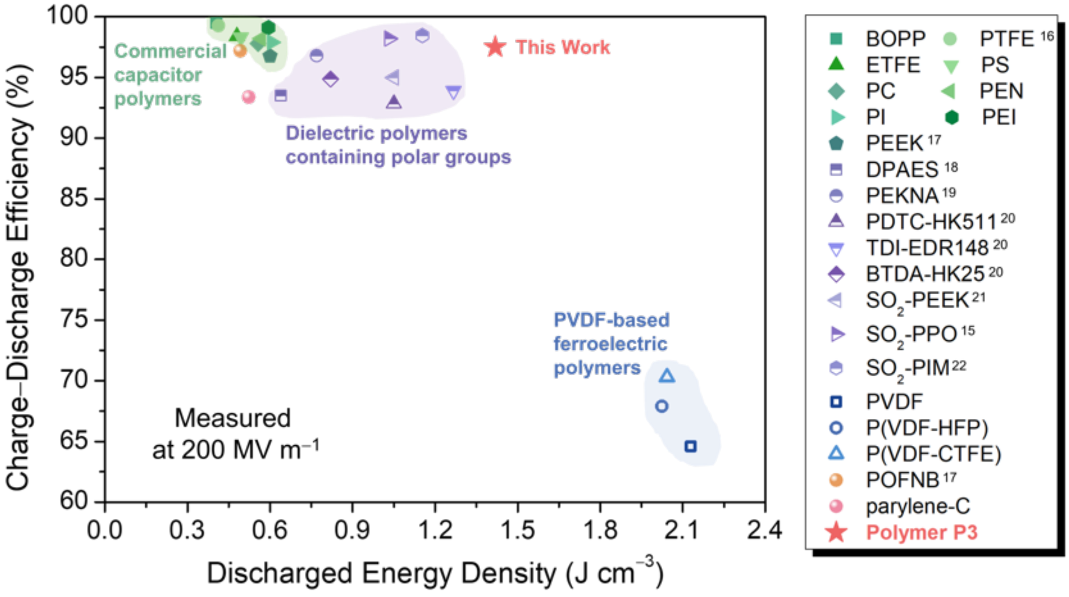

Supplementary Figure 31. Capacitive performance comparison amongst dielectric polymers at 200 MV m^{-1}. Note that all the dielectric polymers are measured at 200 MV m^{-1} with a frequency of 100 Hz except for SO_2-PEEK (10 Hz), SO_2-PPO and SO_2-PIM (1000 Hz).

BOPP: biaxially-oriented polypropylene
PTFE: polytetrafluoroethylene[16]
ETFE: ethylene tetrafluoroethylene
PS: polystyrene
PC: polycarbonate
PEN: poly(ethylene 2,6-naphthalate)
PI: polyimide
PEI: poly(ether imide)
PEEK: polyether ether ketone[17]
DPAES: poly(aryl ether sulfone)[18]
PEKNA: poly(naphthalene ether ketone amide)[19]
PDTC-HK511: polythiourea synthesized from *para*-phenylene diisothiocyanate (PDTC) and Jeffamine HK511[20]
TDI-EDR148: polyurea synthesized from toluene-2,4-diisocyanate (TDI) and Jeffamine EDR148[20]
BTDA-HK25: polyimide synthesized from 3,3',4,4'-benzophenone tetracarboxylic dianhydride (BTDA), Jeffamine HK511 and hexane-1,6-diamine (HDA)[20]
SO_2-PEEK: sulfonylated poly(ether ether ketone)[21]
SO_2-PPO: sulfonylated poly(2,6-dimethyl-1,4-phenylene oxide)[15]
SO_2-PIM: sulfonylated polymers of intrinsic microporosity[22]
PVDF: poly(vinylidene fluoride)
PVDF-HFP: poly(vinylidene fluoride-*co*-hexafluoropropylene)
PVDF-CTFE: poly(vinylidene fluoride-*co*-chlorotrifluoroethylene)
POFNB: polyoxafluoronorbornene[17]

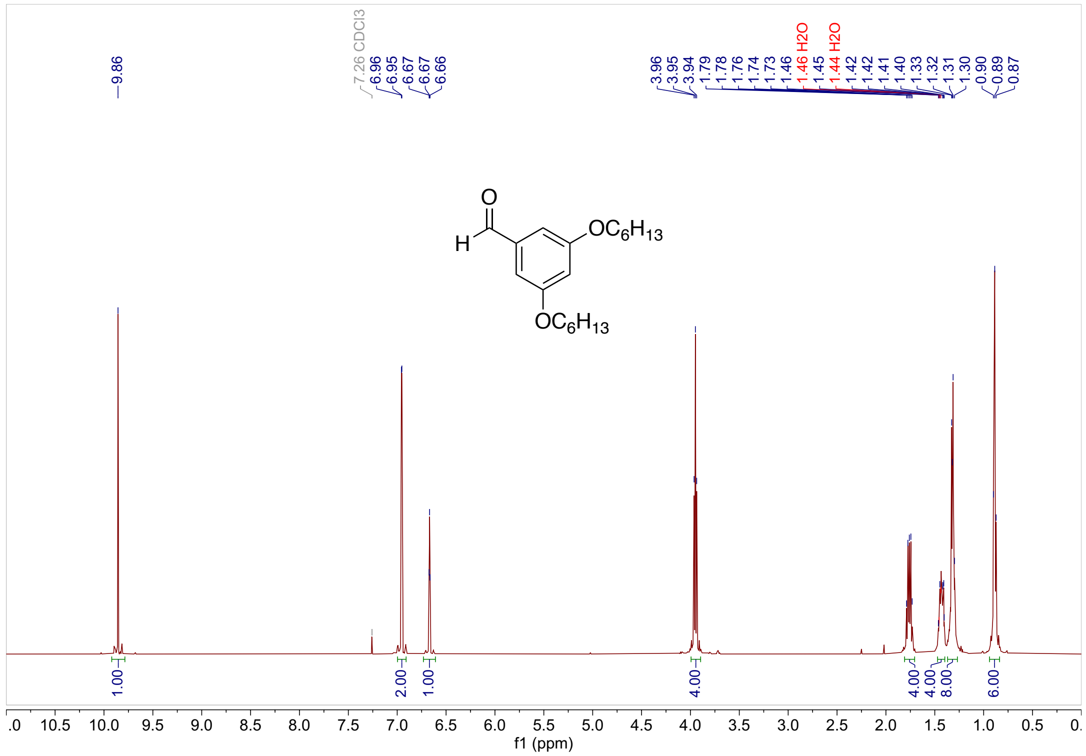

Supplementary Figure 32. ^{1}H NMR Spectrum of 3,5-bis(hexyloxy)benzaldehyde ($CDCl_3$, 298K).

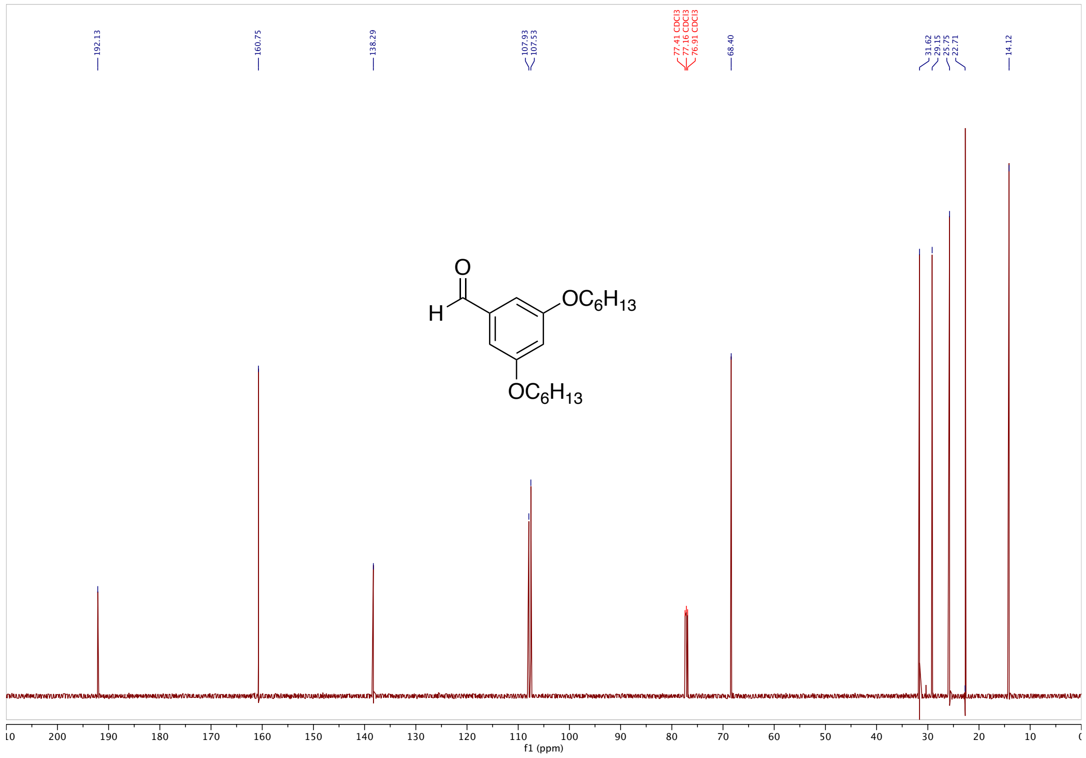

Supplementary Figure 33. ^{13}C NMR Spectrum of 3,5-bis(hexyloxy)benzaldehyde ($CDCl_3$, 298K).

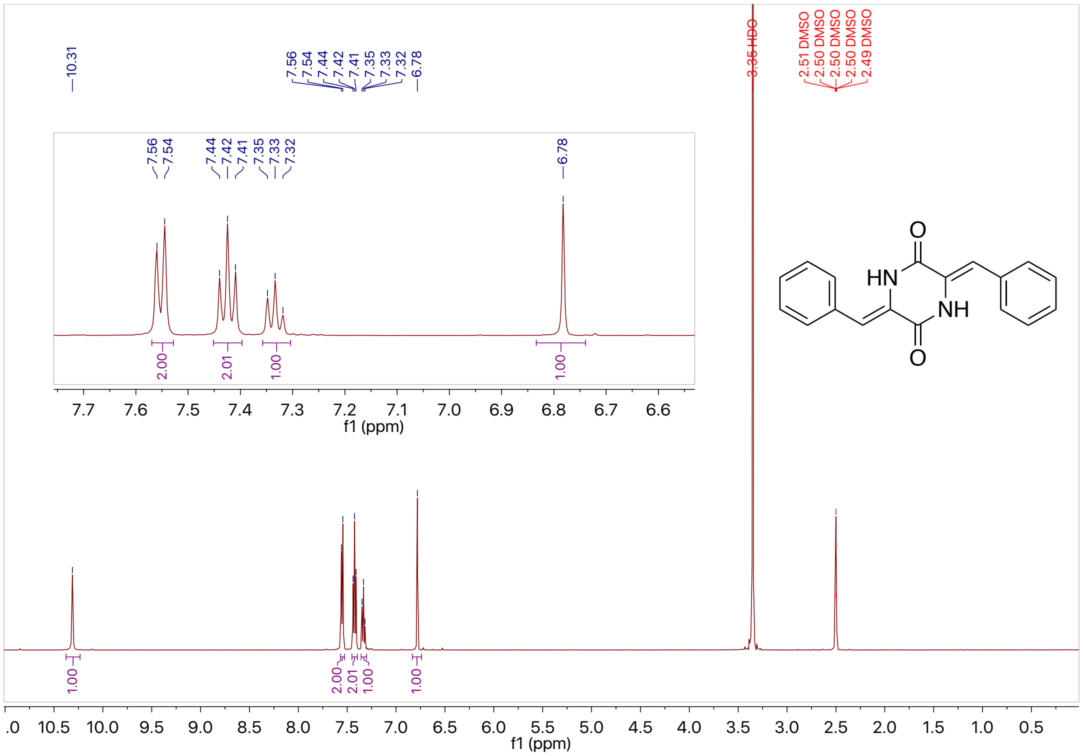

Supplementary Figure 34. ^{1}H NMR Spectrum of compound **6a** (DMSO-d_6, 298K).

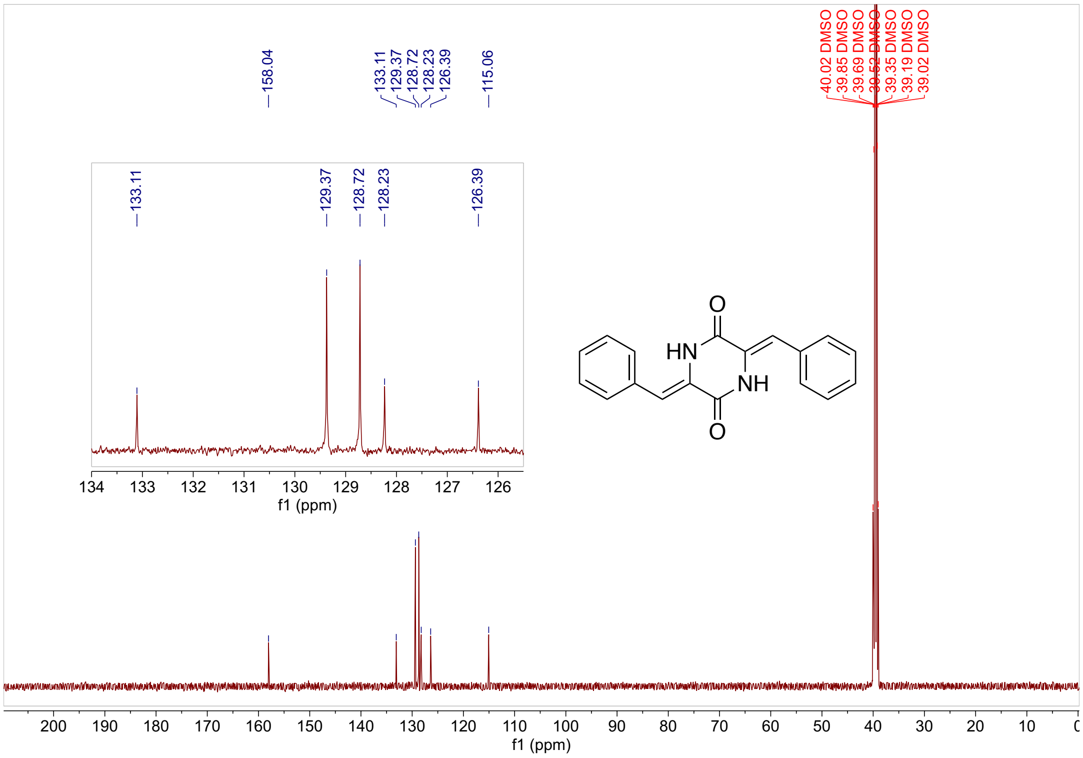

Supplementary Figure 35. ^{13}C NMR Spectrum of compound **6a** (DMSO-d_6, 298K).

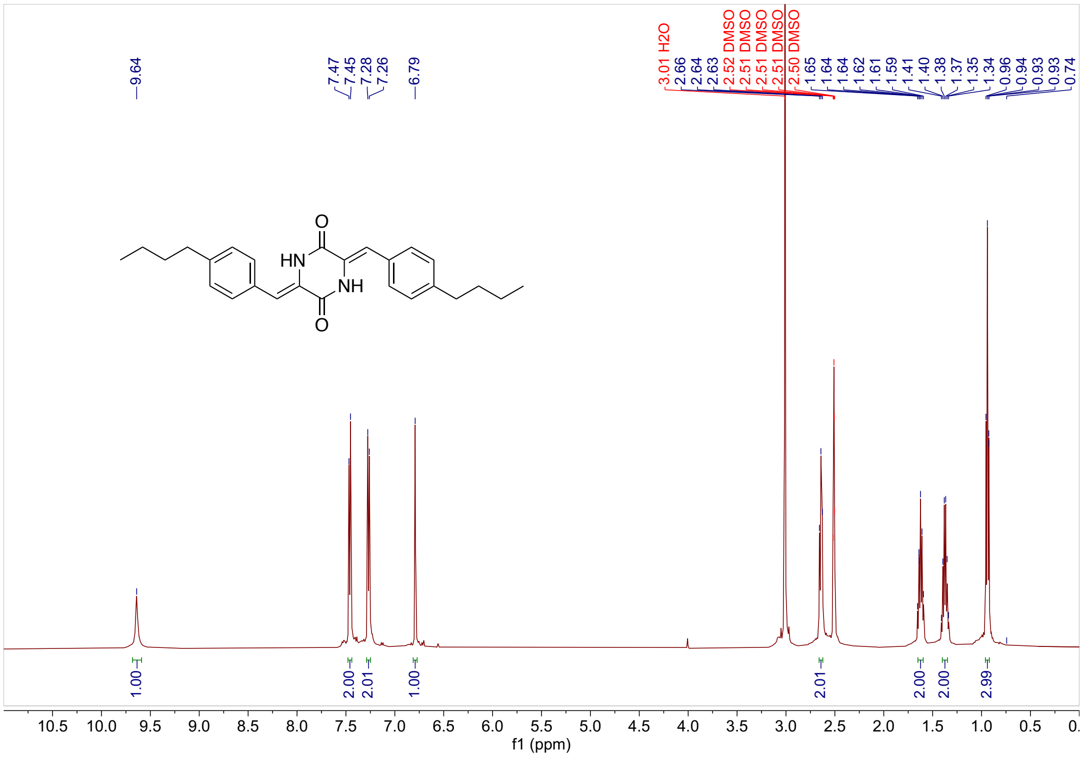

Supplementary Figure 36. ^{1}H NMR Spectrum of compound **6b** (DMSO-d$_6$, 298K).

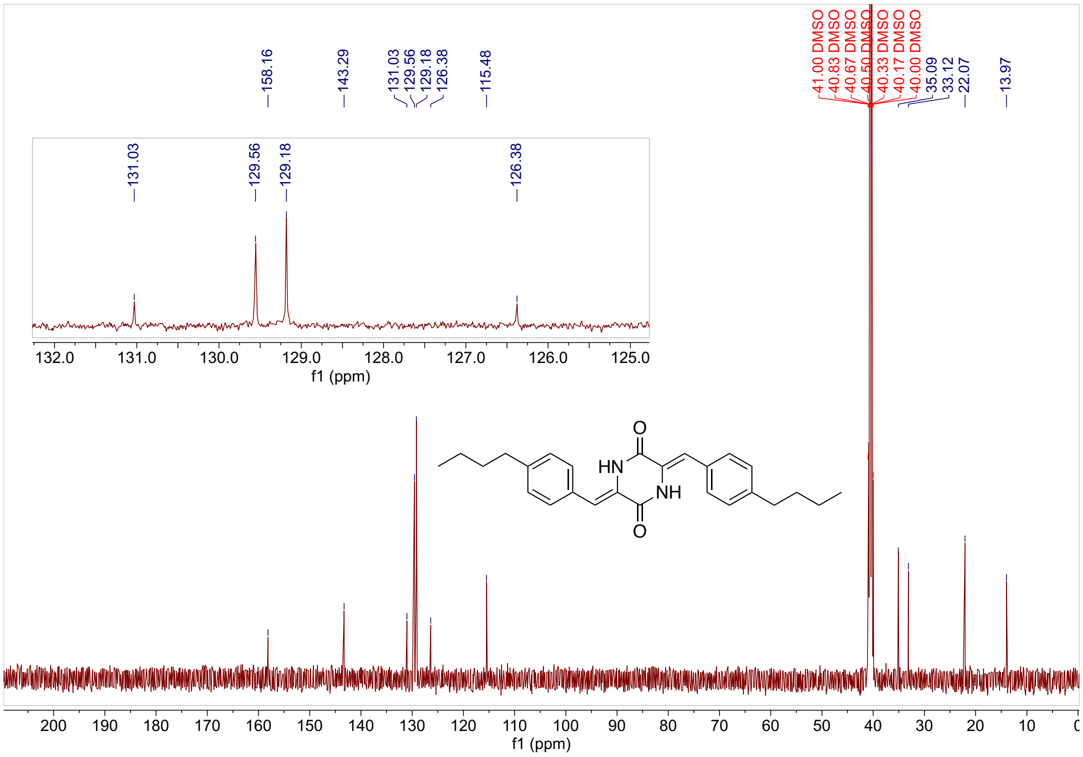

Supplementary Figure 37. ^{13}C NMR Spectrum of compound **6b** (DMSO-d$_6$, 298K).

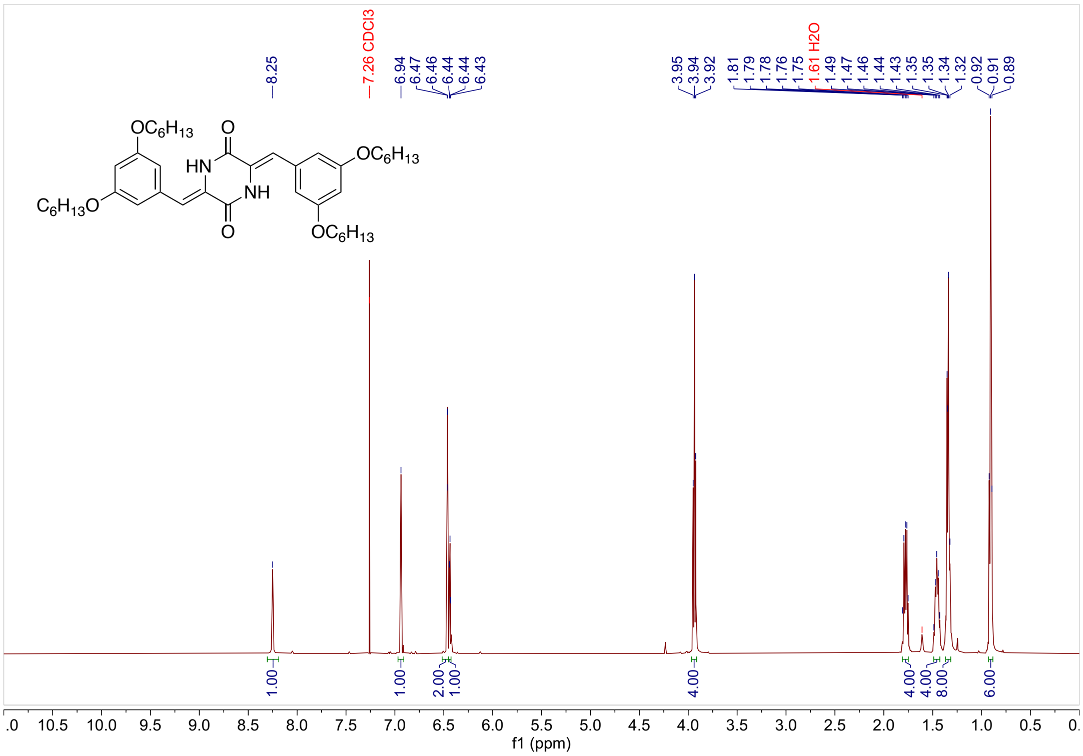

Supplementary Figure 38. ^{1}H NMR Spectrum of compound **6c** ($CDCl_3$, 298K).

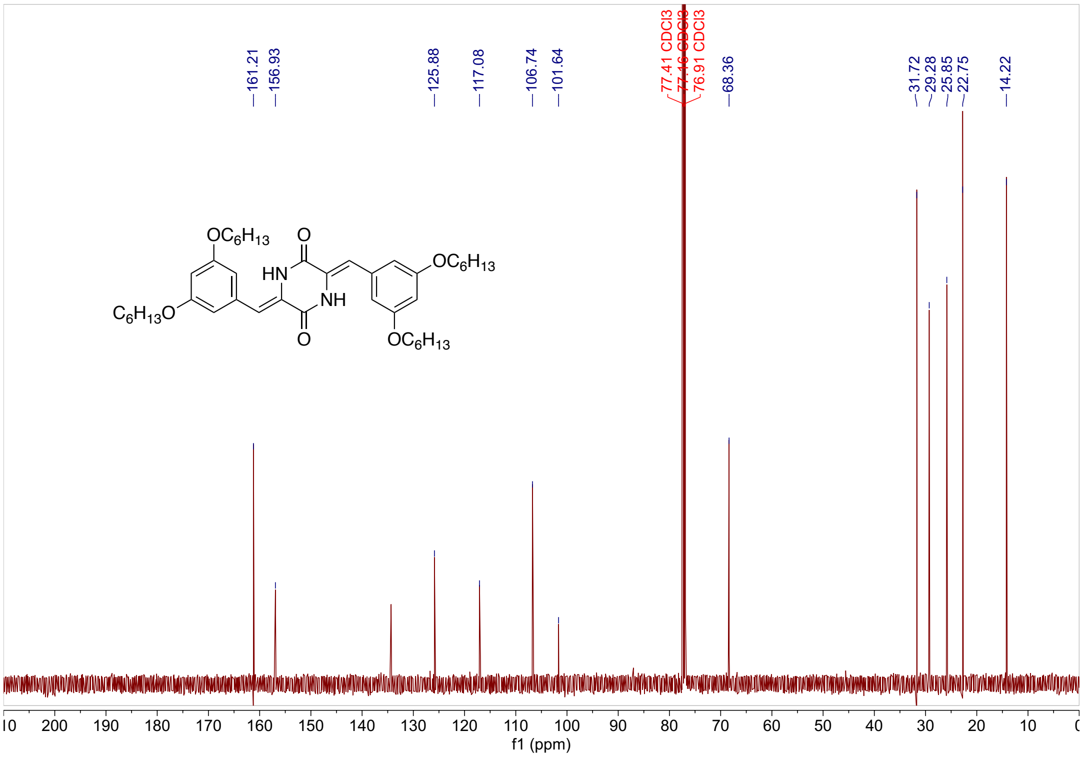

Supplementary Figure 39. ^{13}C NMR Spectrum of compound **6c** ($CDCl_3$, 298K).

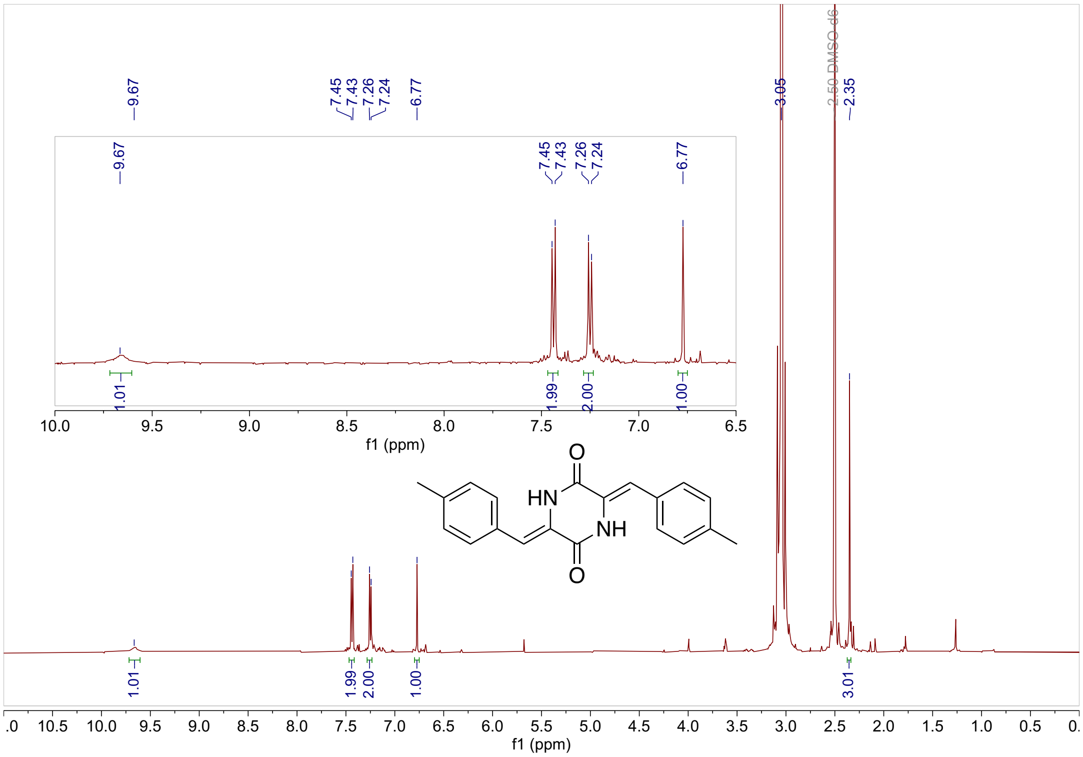

Supplementary Figure 40. ^{1}H NMR Spectrum of compound **6d** (DMSO-d_6, 398K).

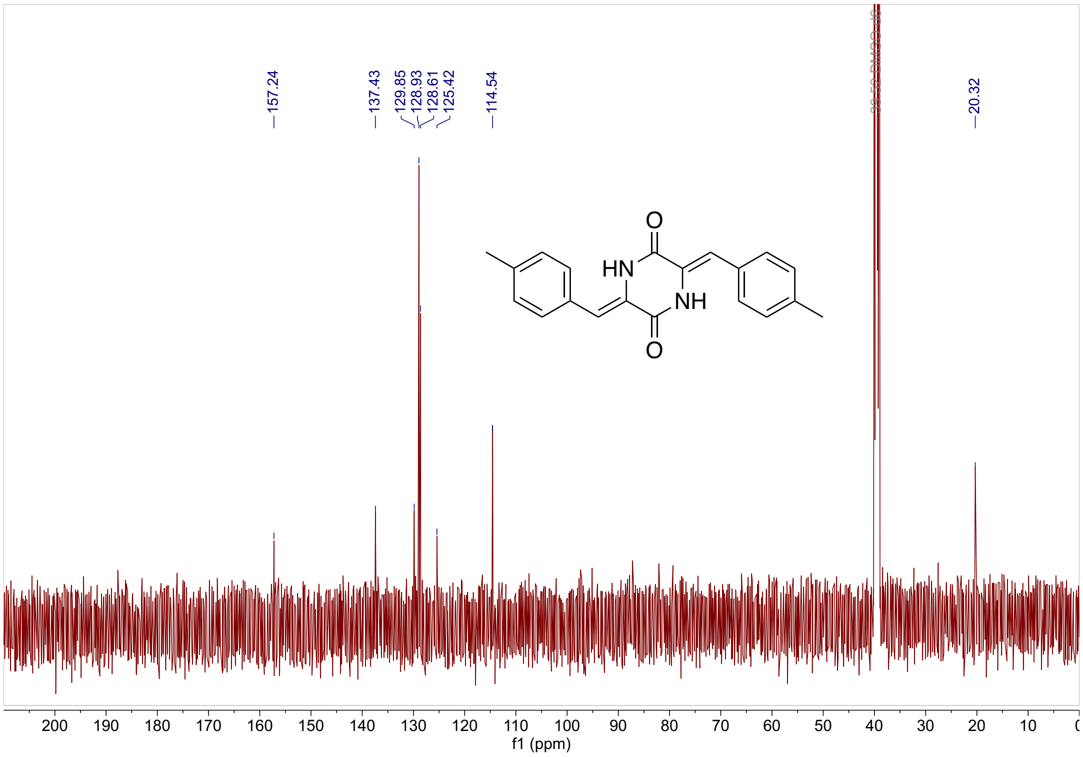

Supplementary Figure 41. ^{13}C NMR Spectrum of compound **6d** (DMSO-d_6, 398K).

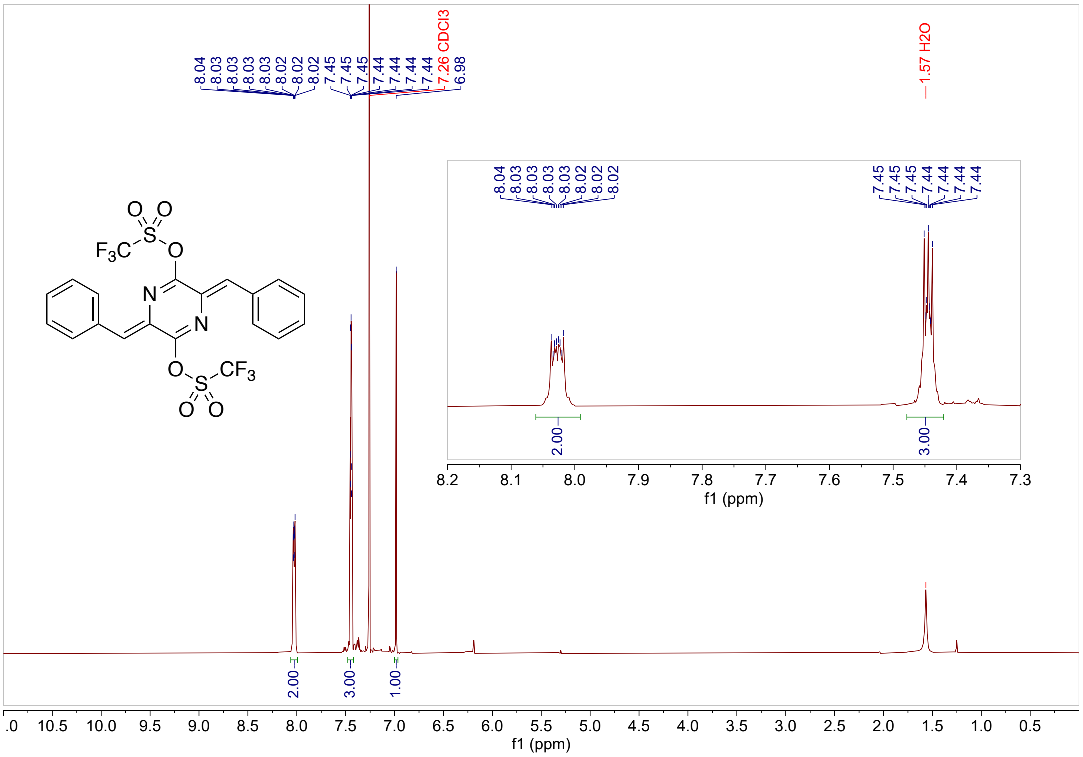

Supplementary Figure 42. ^{1}H NMR Spectrum of monomer **1** ($CDCl_3$, 298K).

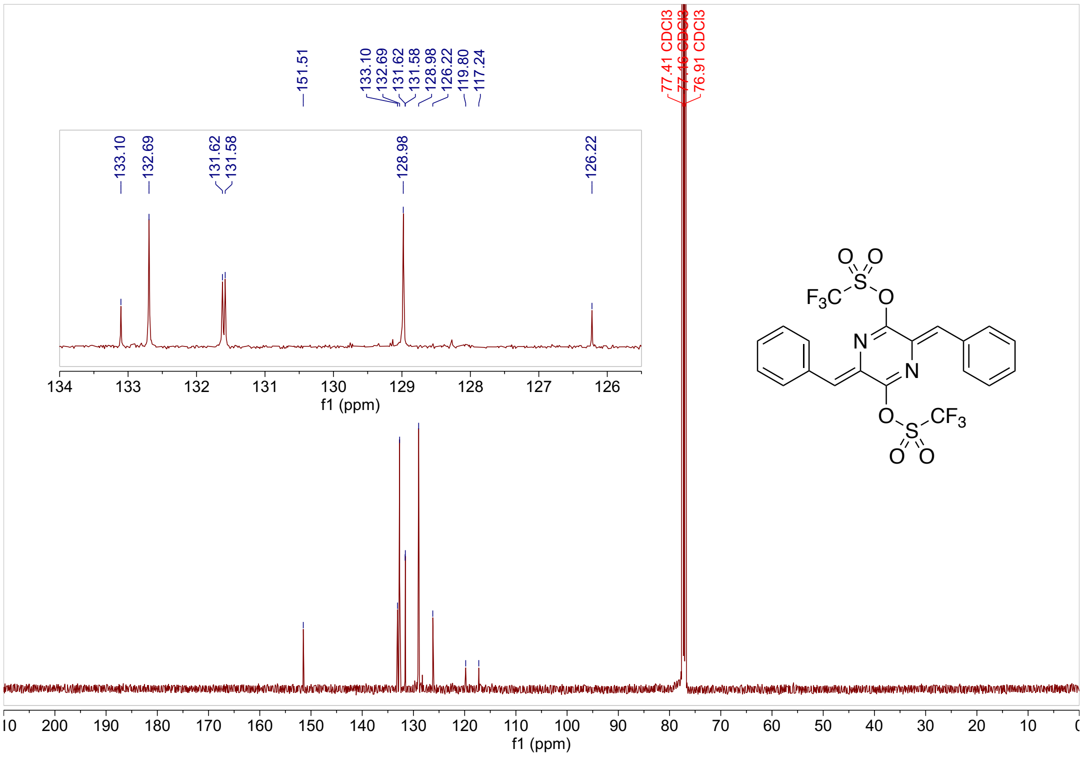

Supplementary Figure 43. ^{13}C NMR Spectrum of monomer **1** ($CDCl_3$, 298K).

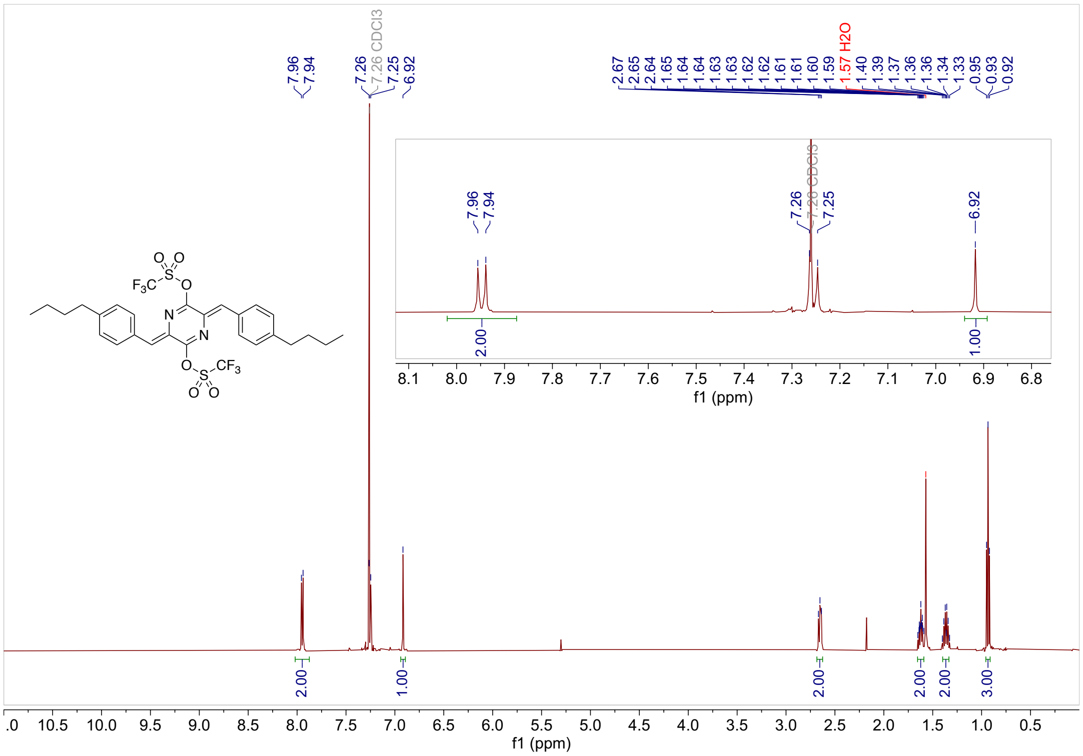

Supplementary Figure 44. ^{1}H NMR Spectrum of monomer **2** ($CDCl_3$, 298K).

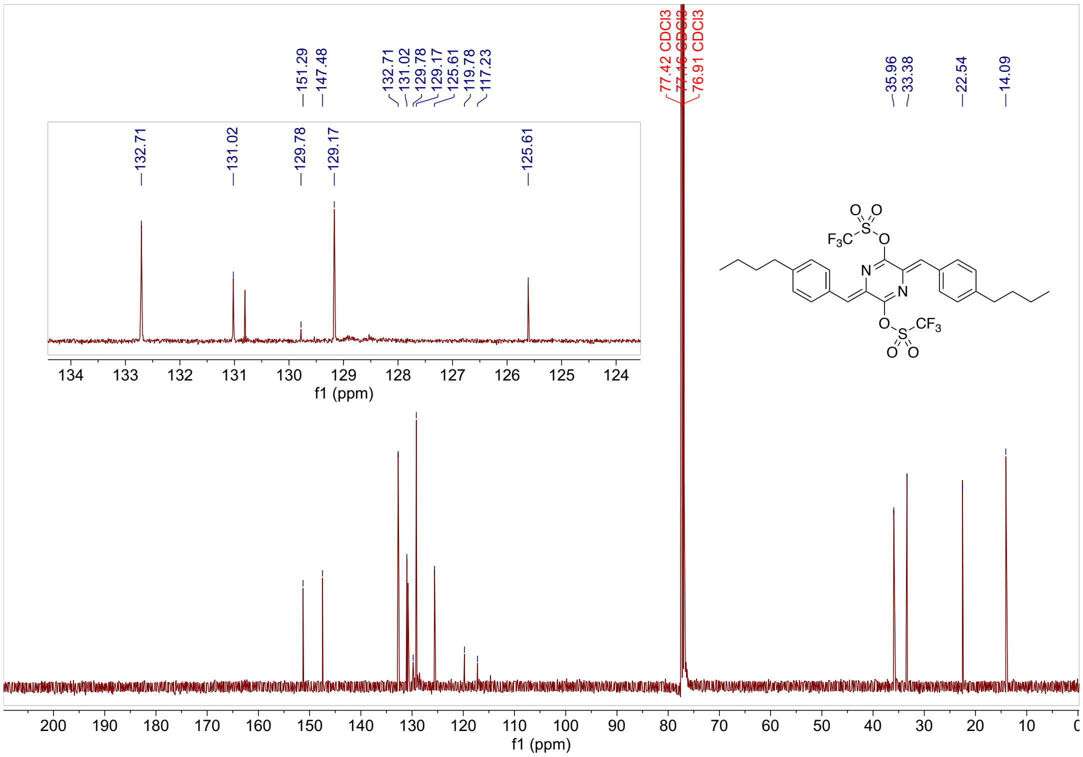

Supplementary Figure 45. ^{13}C NMR Spectrum of monomer **2** ($CDCl_3$, 298K).

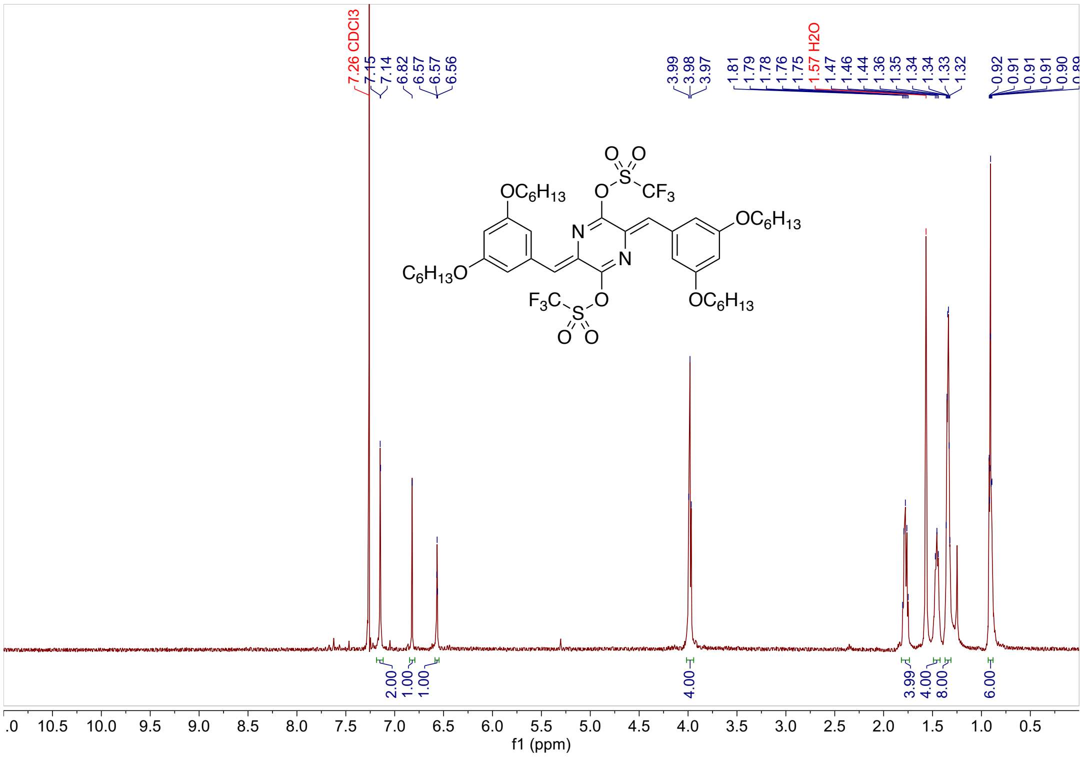

Supplementary Figure 46. ^{1}H NMR Spectrum of monomer **3** ($CDCl_3$, 298K).

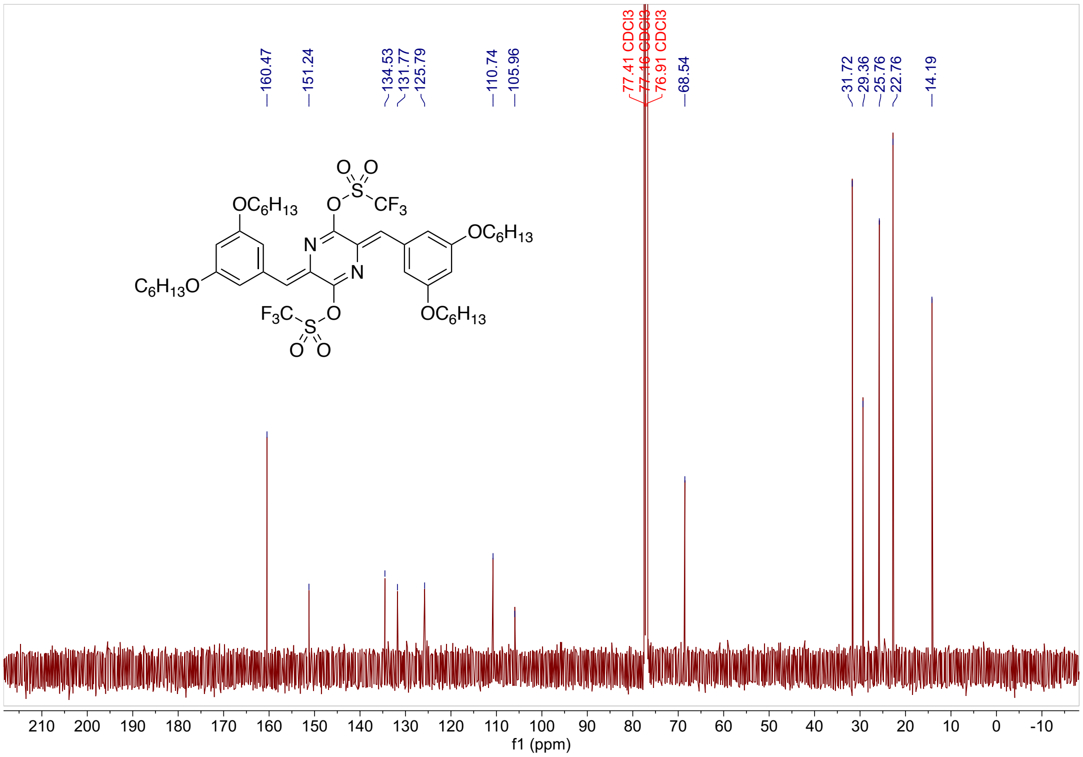

Supplementary Figure 47. ^{13}C NMR Spectrum of monomer **3** ($CDCl_3$, 298K).

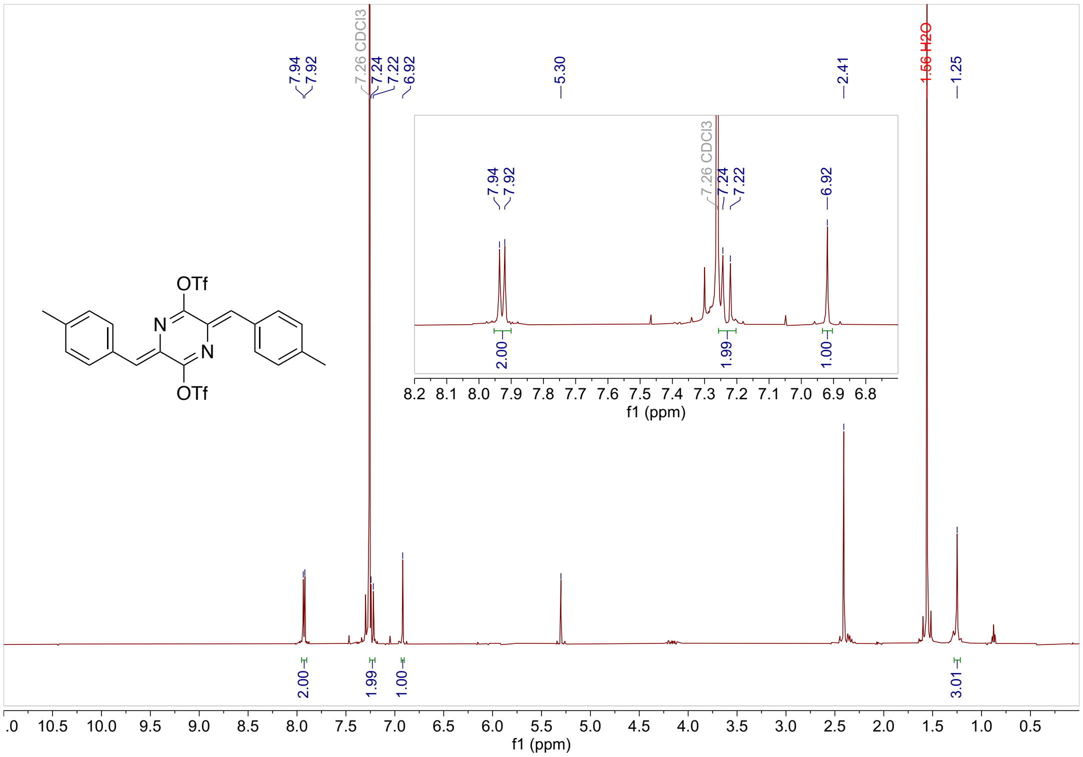

Supplementary Figure 48. ^{1}H NMR Spectrum of monomer **4** ($CDCl_3$, 298K).

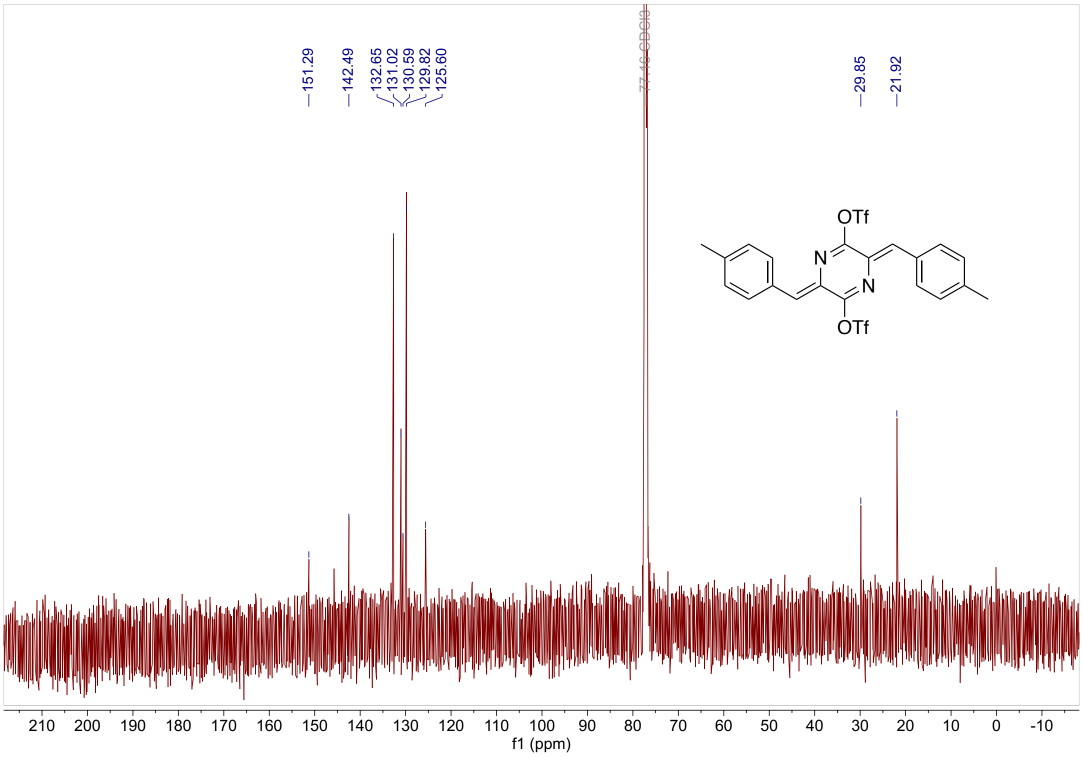

Supplementary Figure 49. ^{13}C NMR Spectrum of monomer **4** ($CDCl_3$, 298K).

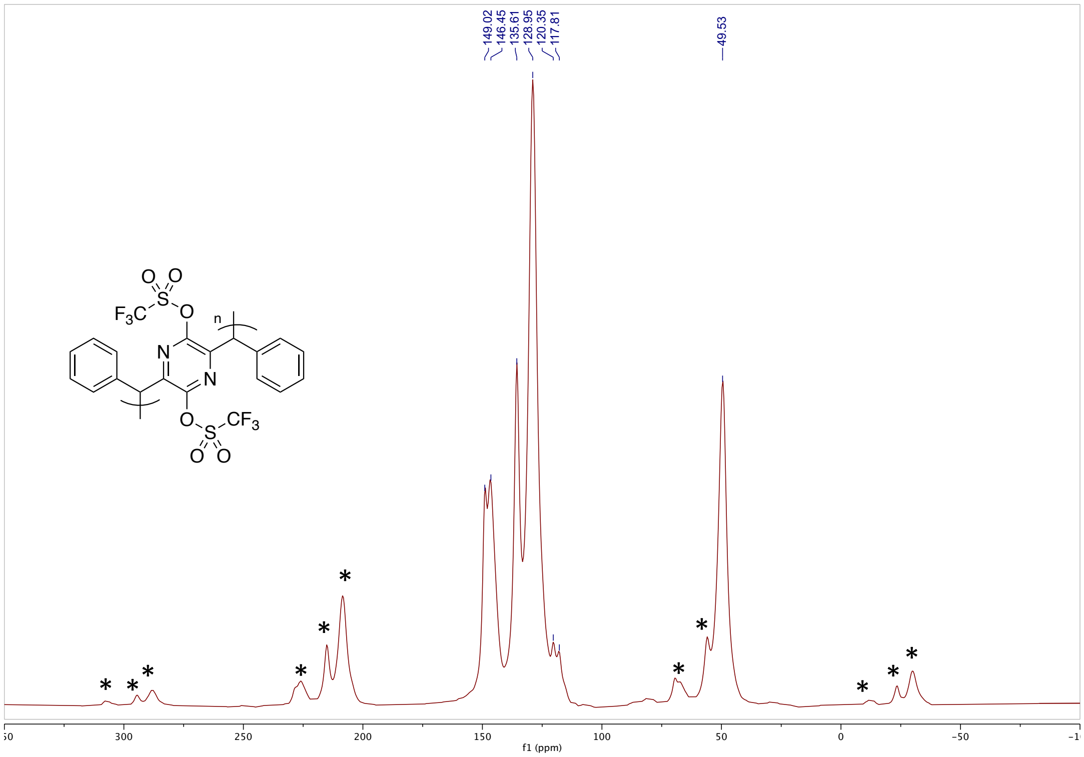

Supplementary Figure 50. Solid-state ^{13}C NMR (CP-MAS) Spectrum of polymer **P1** (298K). Note: Asterisked peaks denote spinning-side bands of the cluster of peaks between 100-175 ppm.

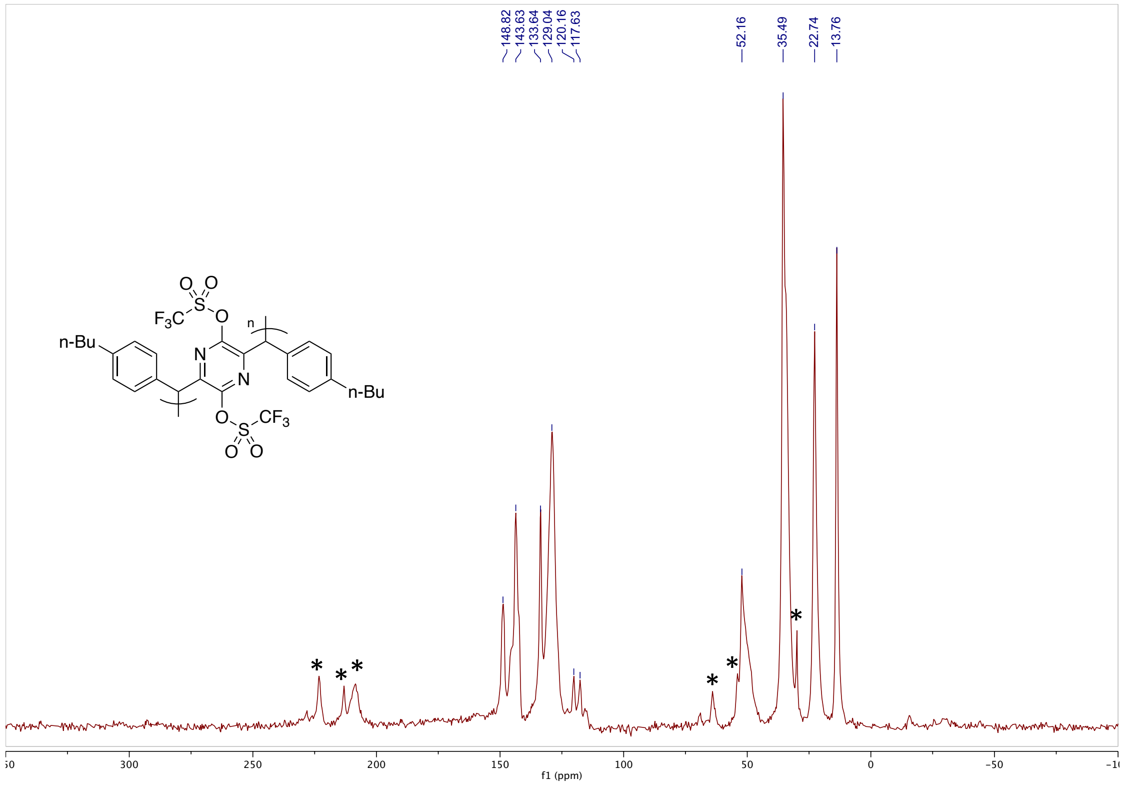

Supplementary Figure 51. Solid-state ^{13}C NMR (CP-MAS) Spectrum of polymer **P2** (298K). Note: Asterisked peaks denote spinning-side bands of the cluster of peaks between 100-175 ppm.

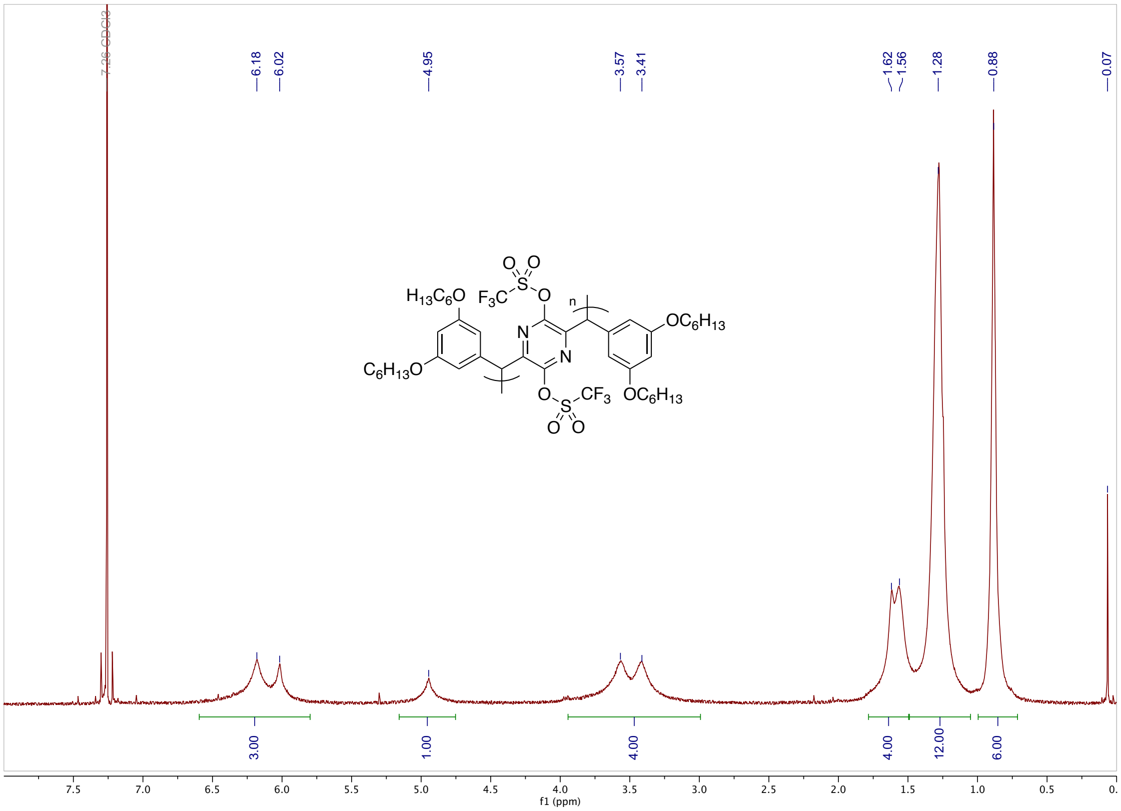

Supplementary Figure 52. ^{1}H NMR Spectrum of polymer **P3** ($CDCl_3$, 298K).

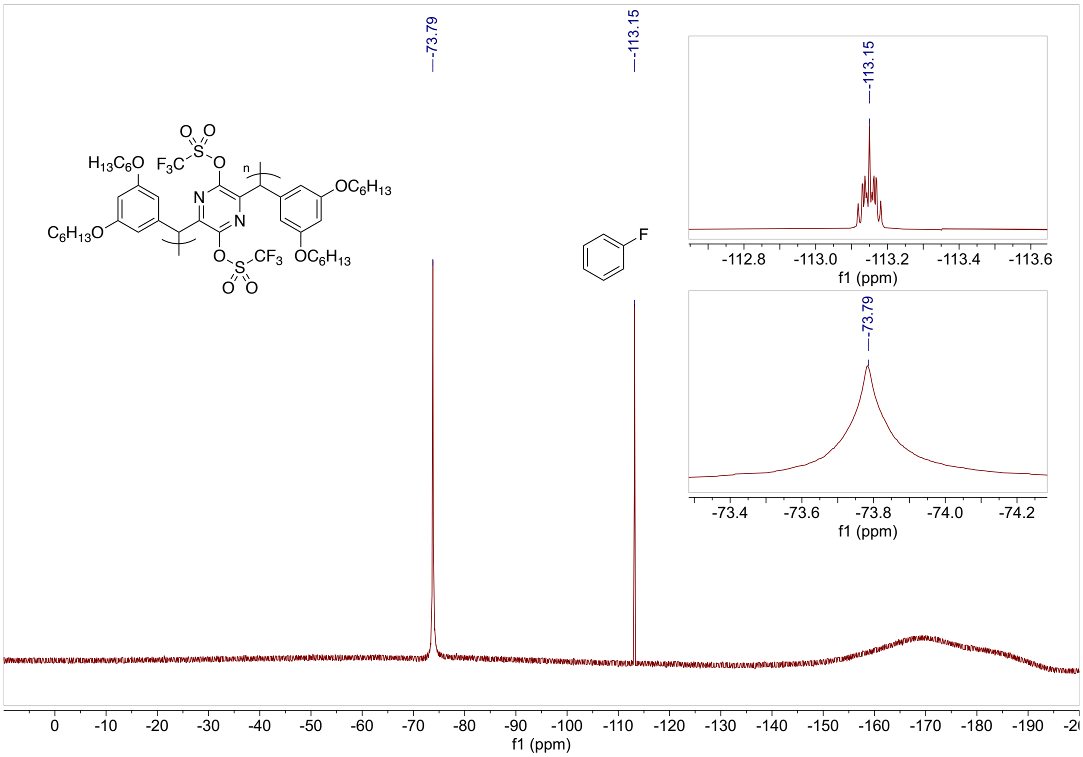

Supplementary Figure 53. ^{19}F NMR Spectrum of polymer **P3** ($CDCl_3$, 298K). Insets: zoomed-in subspectra of each peak to show broadening of the triflate peak. Note: spiked with 30 mM fluorobenzene as an internal standard at -113.15 ppm.

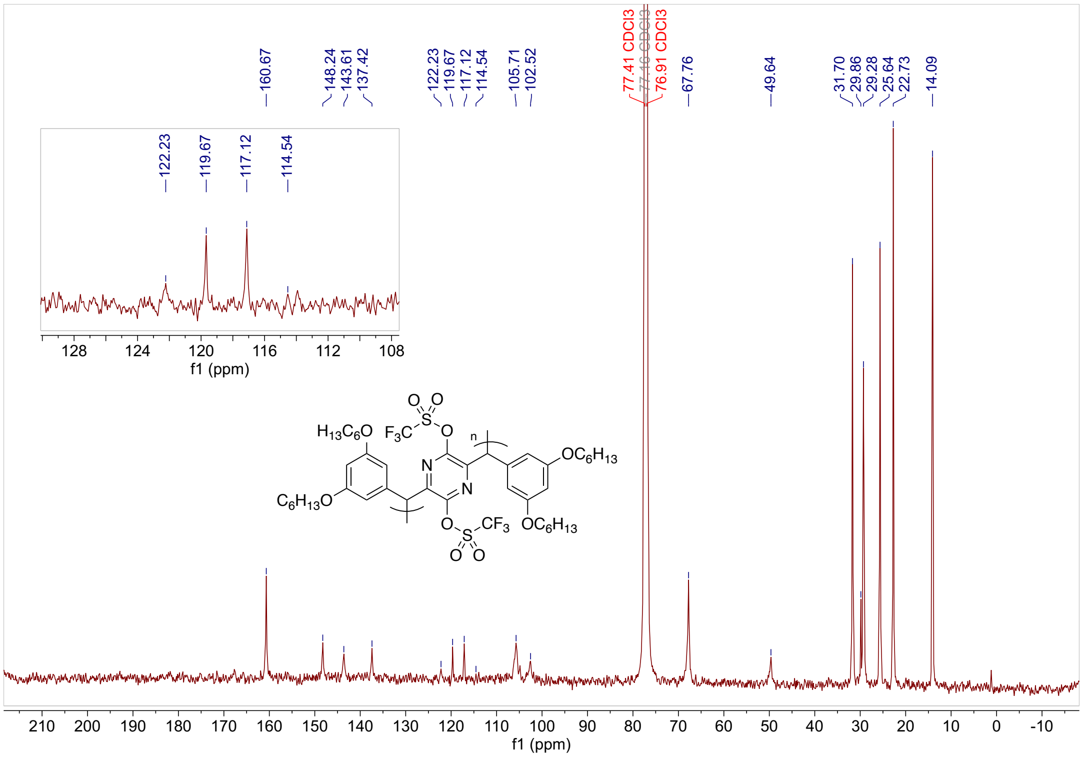

Supplementary Figure 54. ^{13}C NMR Spectrum of polymer **P3** ($CDCl_3$, 298K).

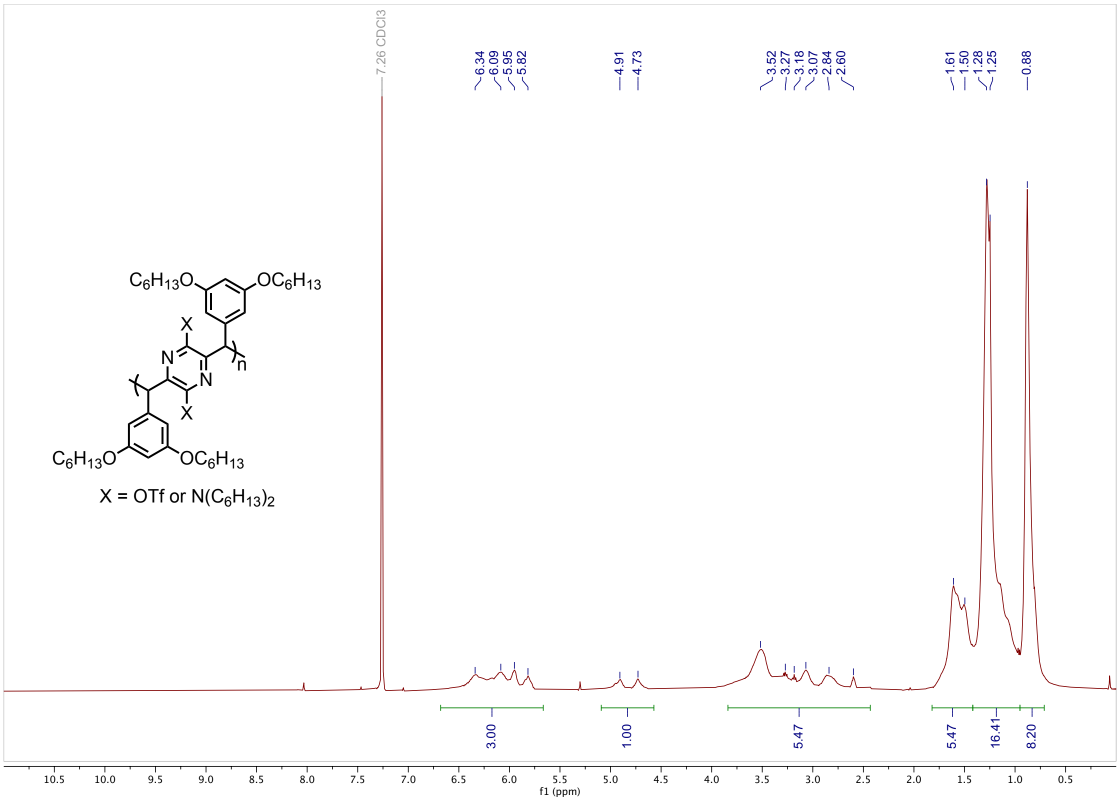

Supplementary Figure 55. ^{1}H NMR Spectrum of polymer **P3-A** ($CDCl_3$, 298K).

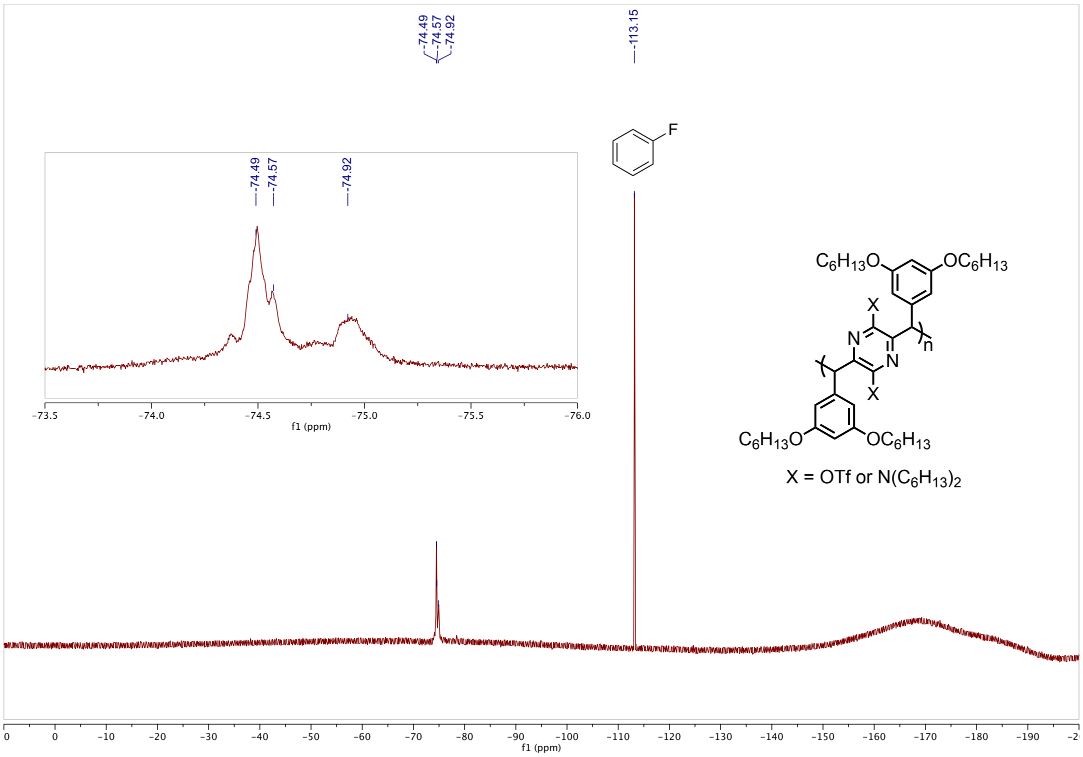

Supplementary Figure 56. ^{19}F NMR Spectrum of polymer **P3-A** ($CDCl_3$, 298K). Spiked with 30 mM fluorobenzene as an internal standard. Fluorobenzene ^{19}F signal set to -113.15 ppm.

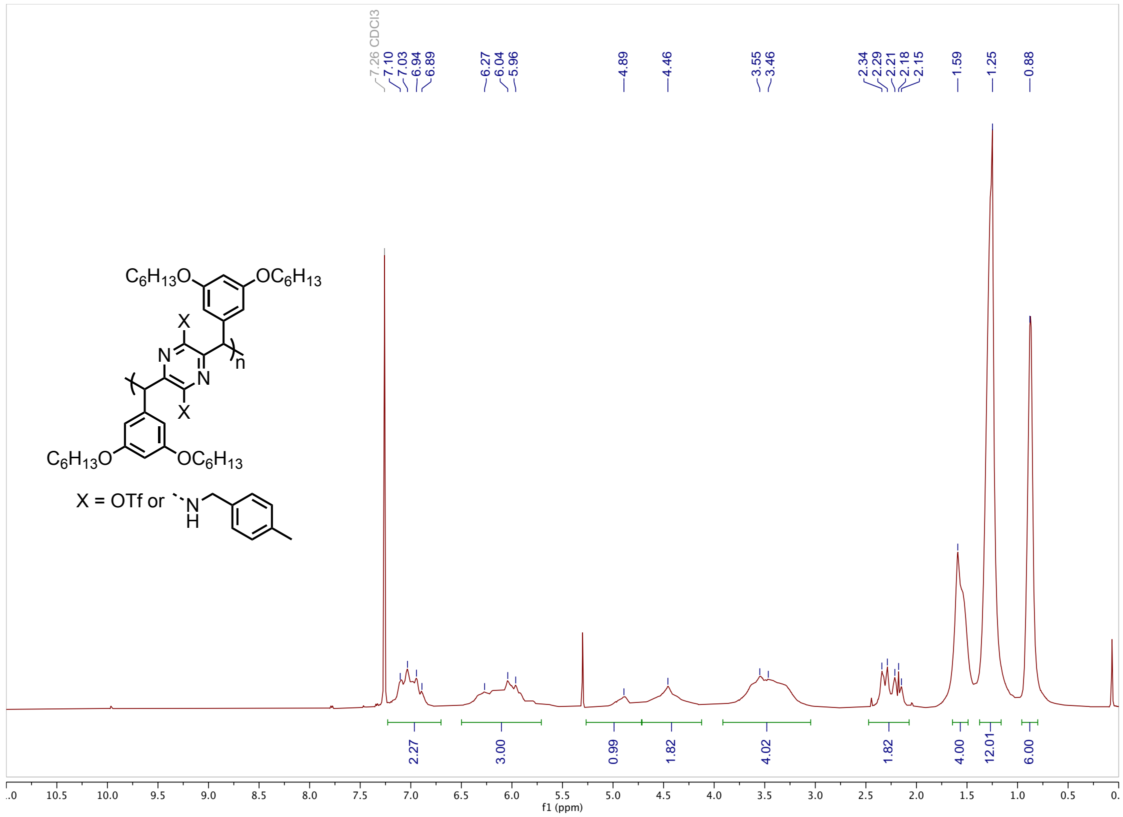

Supplementary Figure 57. ^{1}H NMR Spectrum of polymer **P3-B** ($CDCl_3$, 298K).

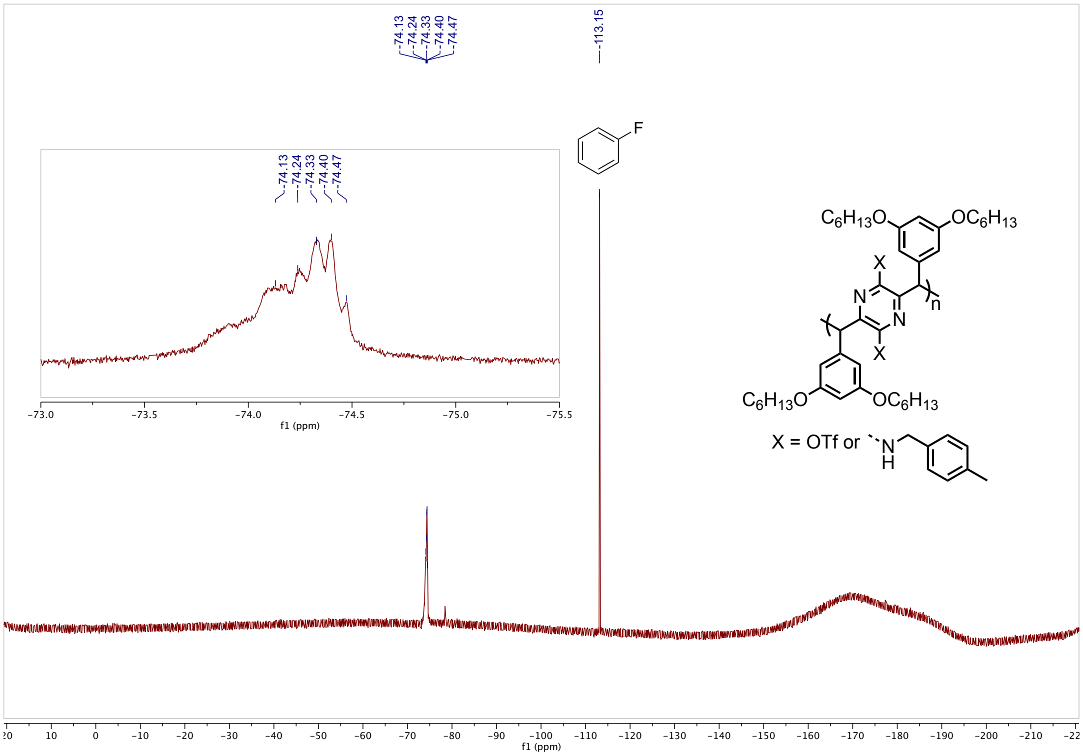

Supplementary Figure 58. ^{19}F NMR Spectrum of polymer **P3-B** ($CDCl_3$, 298K). Spiked with 30 mM fluorobenzene as an internal standard. Fluorobenzene ^{19}F signal set to -113.15 ppm.

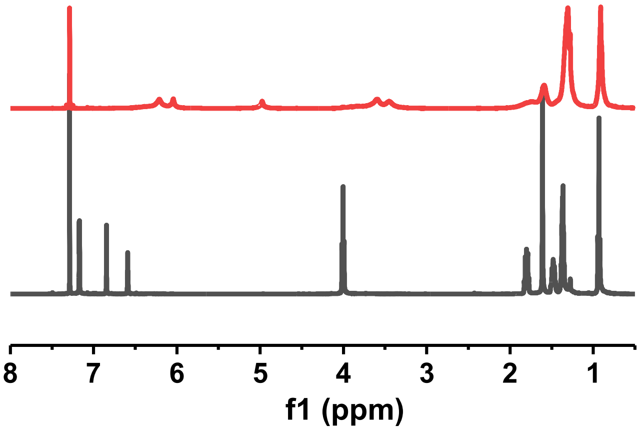

Supplementary Figure 59. Overlay of ^{1}H NMR spectra ($CDCl_3$, 298K) of monomer **3** (bottom) and polymer **P3** (top).

Supplementary References

1 Shao, Y. et al. Advances in molecular quantum chemistry contained in the Q-Chem 4 program package. *Mol. Phys.* **113,** 184–215 (2015).

2 Rappe, A. K., Casewit, C. J., Colwell, K. S., Goddard, W. A. & Skiff, W. M. UFF, a full periodic table force field for molecular mechanics and molecular dynamics simulations. *J. Am. Chem. Soc.* **114,** 10024–10035 (1992).

3 Sheldrick, G. M. SADABS, Bruker Analytical X-ray Systems, Inc., Madison, WI, 2000.

4 Sheldrick, G. M. SHELXT—Integrated space-group and crystal-structure determination. *Acta Crystallogr. A* **71,** 3–8 (2015).

5 Sheldrick, G. M. A short history of SHELX. *Acta Crystallogr. A* **64,** 112–122 (2008).

6 Hattne, J. et al. MicroED data collection and processing. *Acta Crystallogr. A* **71,** 353–360 (2015).

7 Kabsch, W. XDS. *Acta Crystallogr. D* **66,** 125–132 (2010).

8 Sheldrick, G. Crystal structure refinement with SHELXL. *Acta Crystallogr. C* **71,** 3–8 (2015).

9 Hubschle, C. B., Sheldrick, G. M. & Dittrich, B. ShelXle: A Qt graphical user interface for SHELXL. *J. Appl. Crystallogr.* **44,** 1281–1284 (2011).

10 Peng, L. M. Electron atomic scattering factors and scattering potentials of crystals. *Micron* **30,** 625–648 (1999).

11 Zhou, Y. & Wang, Q. Advanced polymer dielectrics for high temperature capacitive energy storage. *J. Appl. Phys.* **127,** 240902 (2020).

12 Montanari, D. et al. Film capacitors for automotive and industrial applications. Vol. 29 (2009).

13 Yang, L.-M., Rong-Yang, W., McPhail, A. T., Yokoi, T. & Lee, K.-H. Neihumicin, a new cytotoxic antibiotic from micromonospora neihuensis II. Structural determination and total synthesis. *J. Antibiot.* **41,** 488–493 (1988).

14 Zhu, L. & Wang, Q. Novel ferroelectric polymers for high energy density and low loss dielectrics. *Macromolecules* **45,** 2937–2954 (2012).

15 Zhang, Z., Wang, D. H., Litt, M. H., Tan, L.-S. & Zhu, L. High-temperature and high-energy-density dipolar glass polymers based on sulfonylated poly(2,6-dimethyl-1,4-phenylene oxide). *Angew. Chem. Int. Ed.* **57,** 1528–1531 (2018).

16 Luo, S. et al. Elaborately fabricated polytetrafluoroethylene film exhibiting superior high-temperature energy storage performance. *Appl. Mater. Today* **21,** 100882 (2020).

17 Wu, C. et al. Flexible temperature-invariant polymer dielectrics with large bandgap. *Adv. Mater.* **32,** 2000499 (2020).

18 Xu, W. et al. Bioinspired polymer nanocomposites exhibit giant energy density and high efficiency at high temperature. *Small* **15,** 1901582 (2019).

19 Xu, D. et al. Rational design of soluble polyaramid for high-efficiency energy storage dielectric materials at elevated temperatures. *Macromol. Mater. Eng.* **305,** 1900820 (2020).

20 Li, Z. et al. High energy density and high efficiency all-organic polymers with enhanced dipolar polarization. *J. Mater. Chem. A* **7,** 15026–15030 (2019).

21 Wei, J. et al. High dielectric constant dipolar glass polymer based on sulfonylated poly(ether ether ketone). *Polymer* **178,** 121688 (2019).

22 Zhang, Z. et al. High-κ polymers of intrinsic microporosity: a new class of high temperature and low loss dielectrics for printed electronics. *Mater. Horiz.* **7,** 592–597 (2020).
