# Supplementary material for: Solution-processable and functionalizable ultra-high molecular weight polymers via topochemical synthesis
Source: Nat Commun. 2021 Nov 24;12:6818. doi: 10.1038/s41467-021-27090-1 (PMC8613210; doi:10.1038/s41467-021-27090-1)
Supplement: Supplementary file 2 — Description of Additional Supplementary Files [file 41467_2021_27090_MOESM2_ESM.pdf]

Description of Additional Supplementary Files

File Name: Supplementary Movie 1
Description: Optical changes of the monomer **1** crystal upon topochemical polymerization
